# Employing epigenetic protein degradation techniques to block CCL5-mediated photodynamic therapy via a programmed delivery platform

**DOI:** 10.1038/s41392-025-02542-y

**Published:** 2026-01-30

**Authors:** Tingting Yang, Yuzhu Hu, Anjie Guo, Xifeng Zhang, Wanyu Wang, Linbin Yi, Rui Zhang, Xinyu Gou, Zhiyong Qian, Bilan Wang, Yongzhong Cheng, Xiang Gao

**Affiliations:** 1 Department of Neurosurgery and Institute of Neurosurgery, State Key Laboratory of Biotherapy and Cancer Center, West China Hospital, West China Medical School, Sichuan University and Collaborative Innovation Center for Biotherapy, Chengdu, China; 2 Department of Pharmacy, West China Second University Hospital of Sichuan University, Chengdu, PR China; 3 Evidence-Based Pharmacy Center, West China Second University Hospital, Sichuan University, Chengdu, PR China; 4 Key Laboratory of Birth Defects and Related Diseases of Women and Children, Ministry of Education, West China Second University Hospital, Sichuan University, Chengdu, PR China; 5 Children’s Medicine Key Laboratory of Sichuan Province, Chengdu, PR China

**Keywords:** Drug development, Drug development

## Abstract

Despite the significant potential of photodynamic therapy (PDT) in cancer treatment, further refinement is needed to address challenges such as poor tumor-specific accumulation of photosensitizers and the development of therapeutic resistance, which may be regulated by epigenetics. Here, a novel tumor microenvironment-responsive delivery platform was developed to co-deliver epigenetic protein degraders and photosensitizers, aiming to block the relevant regulatory mechanisms and enhance the effectiveness of combination therapy. Benefiting from the targeting ability, pH-triggered charge reversal, and intracellular glutathione (GSH)-responsive release, the delivery platform exhibited enhanced tumor accumulation and therapeutic effects. The mechanism of action revealed that the precise accumulation and release of drugs via the tumor-orchestrated delivery system not only regulated cell growth and immune activation, but also inhibited the expression of tumor immune escape molecules (PDL1 and CD47) and M2 macrophage polarization, significantly increasing the anti-breast cancer and anti-melanoma effects of PDT in the presence of an epigenetic modifier. More importantly, we found for the first time that photodynamic therapy can generate therapeutic resistance through the upregulation of CCL5, and confirmed that this resistance can be reduced by the epigenetic degradation of bromodomain-containing protein 4 (BRD4). These findings underscore the potential of integrating PDT with epigenetic protein degraders through a programmed delivery platform, offering a promising strategy for improving cancer treatment outcomes.

## Introduction

Traditional interventions for cancer frequently present numerous disadvantages, such as postoperative recurrence and metastasis, harmful side effects, and tumor resistance.^1–3^ These challenges have prompted the development of alternative therapeutic strategies, among which photodynamic therapy (PDT) has emerged as a promising approach. PDT, a minimally invasive cancer treatment, uses photosensitizers and specific laser wavelengths to generate reactive oxygen species (ROS) that eliminate pathological lesions by inducing cell death, damaging the vasculature, and modulating the immune response, which holds significant potential for the treatment of tumors that respond well to PDT, such as breast cancer and melanoma.^4–6^ Compared with conventional therapies such as radiotherapy and chemotherapy, PDT has several advantages, including minimal trauma, high selectivity, and reduced systemic toxicity.^7^ However, given the complexity of tumors, the efficacy of PDT is strongly contingent upon the tumor-specific accumulation of photosensitizers and is limited by the activation of resistance mechanisms.^8^ Thus, the development of innovative therapeutic strategies to overcome the limitations of PDT efficacy is essential for improving its clinical application in tumor treatment.

Research indicates that resistance to PDT arises from multiple mechanisms, including the activation of antioxidant systems, DNA repair pathways, and pro-survival signaling cascades, as well as the presence of hypoxic and immunosuppressive tumor microenvironments.^9^ To overcome these challenges, combination strategies have been developed to reduce PDT resistance, including PARP inhibitors that block DNA repair,^10^ ACAT-1 inhibitors that increase T-cell function by disrupting cholesterol metabolism,^11^ and Cas9 RNP-mediated knockout of the antioxidant regulator NRF2.^12^ Improving the therapeutic efficacy of PDT from the perspective of treatment mechanisms is a key direction for tumor-targeted PDT. However, the mechanisms underlying PDT resistance remain insufficiently understood and require further investigation. Moreover, targeting a single resistance mechanism may not fully enhance the efficacy of PDT. Therefore, developing innovative strategies that combine multiple enhancement mechanisms while inhibiting resistance is crucial for advancing the clinical application of PDT in cancer therapy. Notably, epigenetic modifications, such as post-translational methylation and acetylation of histones, play crucial regulatory roles in these resistance mechanisms, contributing to tumor immune evasion and resistance to chemotherapy and radiotherapy.^13–15^ Therefore, the application of advanced biotechnology to interfere with key epigenetic molecules and disrupt tolerance mechanisms holds significant promise for enhancing the efficacy of cancer PDT in multiple ways. Proteolysis-targeting chimeras (PROTACs) are designed molecules that induce targeted protein degradation by linking a target protein to an E3 ubiquitin ligase, leading to protein destruction via the ubiquitin‒proteasome system.^16^ PROTACs have been successfully applied in the degradation of epigenetic proteins,^17^ such as bromodomain-containing protein 4 (BRD4), and have shown potential for optimizing PDT efficacy. Among them, ARV-825 is a representative BRD4-targeting PROTAC molecule that induces efficient and sustained BRD4 degradation, offering advantages over traditional inhibitors, such as reduced off-target effects and improved therapeutic outcomes. Additionally, our previous research has revealed that ARV-825 not only enhances chemosensitivity but also suppresses M2 macrophage polarization,^18,19^ thus demonstrating significant potential for enhancing PDT efficacy and reducing resistance.

However, photosensitizers and epigenetic protein degraders face common challenges, such as drug hydrophobicity and limited tumor tissue accumulation.^7,20^ Moreover, the cytotoxic effects of the ROS generated by PDT underscore the need for precise control over photosensitizer release within tumor cells to optimize treatment efficacy. Therefore, optimizing the combination strategy to fully exploit its maximum efficacy is a key issue that needs to be addressed. In recent years, advancements in nanotechnology have offered innovative solutions to these drug delivery limitations through various strategies, such as PEG modification, ligand-receptor-specific recognition, charge-reversal-mediated transcytosis, and tumor microenvironment-responsive controlled release.^21–23^ Notably, polymer carriers, such as polyethylene glycol (PEG), polycaprolactone (PCL), and polyethyleneimine (PEI), stand out for their ability to increase drug solubility, support functionalization, and ensure biodegradability, making them highly promising for combined photodynamic therapy.^24–26^ However, challenges such as off-target effects, limited targeting efficiency, and slow stimulus responsiveness may decrease drug accumulation and release, further hindering efficacy and clinical application.^27,28^ Thus, a multi-synergistic strategy may effectively improve these issues.

In this work, an innovative strategy for treating breast cancer and melanoma was developed by devising a targeted and tumor microenvironment-responsive delivery system capable of simultaneously loading photosensitizers and the BRD4 degrader ARV-825. This delivery system, which is based on the CAPIR (circulation, accumulation, penetration, internalization, and release) cascade,^29^ comprises the redox-responsive MPEG-SS-PCL, targeted PCL-PEG-cRGD, and charge-reversal PCL-PEG-PEI-DM polymers to address the challenges associated with the intravenous administration of nanomedicines and enhance tumor accumulation through a multi-synergistic strategy, ultimately maximizing therapeutic efficacy. Furthermore, the physical characteristics, tumor-targeting efficiency, molecular mechanisms, and immune responses of the treatment system were thoroughly evaluated. Our results demonstrated the therapeutic efficacy of this strategy and revealed multiple biological mechanisms through which the BRD4 degrader enhances PDT effectiveness, including the induction of cell death, activation of immune responses, and reversal of PDT resistance. This comprehensive study provides both theoretical and experimental foundations for the clinical translation of tumor-targeted PDT, advancing the development of more efficient cancer treatments.

## Results

### Synthesis and characterization of ARV/Ce6@RDP

**Fig. 1:**
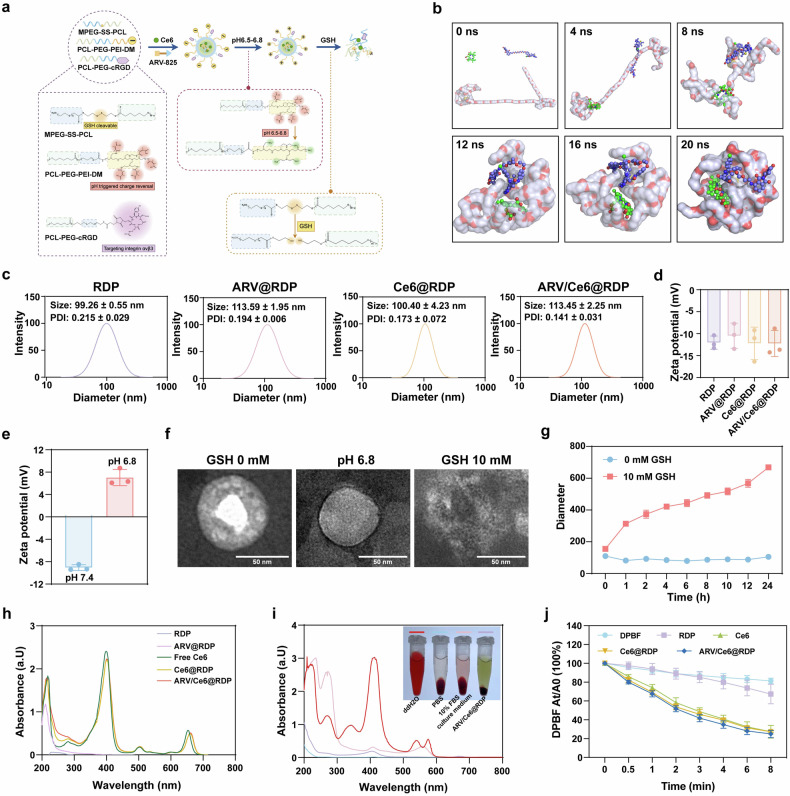
Characterization of ARV/Ce6@RDP. **a** Schematic representation of ARV/Ce6@RDP micelle preparation and the pH/GSH response. The figure was created with Figdraw.com. **b** Computational simulation of the interactions among MPEG-SS-PCL, Ce6, and ARV-825 at different time points in a normal physiological environment (MPEG-SS-PCL: white; Ce6: green; ARV-825: blue). **c** Particle size distributions of the RDP, ARV@RDP, Ce6@RDP, and ARV/Ce6@RDP micelles detected via DLS. **d** Zeta potentials of RDP, ARV@RDP, Ce6@RDP, and ARV/Ce6@RDP micelles detected by DLS (*n* = 3 per group). **e** pH-triggered charge reversal for ARV/Ce6@RDP (*n* = 3 per group). **f** TEM images of ARV/Ce6@RDP under different conditions. Scale bar: 50 nm. **g** GSH-triggered particle size change for ARV/Ce6@RDP (*n* = 3 per group). **h** UV−vis spectra of ARV/Ce6@RDP. **i** Hemolysis evaluation of ARV/Ce6@RDP. **j** Singlet oxygen (^1^O_2_) generation ability as monitored by degradation of DPBF in the presence of Ce6@RDP or ARV/Ce6@RDP under laser irradiation (*n* = 3 per group). All the data in this figure are presented as the means ± SDs

As shown in Fig. 1a, the tumor microenvironment-based ARV/Ce6@RDP micelles were elaborately designed and assembled from biocompatible and easily synthesized polymers (PCL-PEG-PEI-DM, MPEG-SS-PCL, and cRGD-PEG-PCL), the photosensitizer Ce6, and the BRD4 degrader ARV-825. The PCL-PEG-PEI-DM polymer offers the advantage of charge reversal, exhibiting a negative charge under normal physiological conditions to extend the blood circulation time and reduce non-target tissue uptake, while reversing to a positive charge under acidic conditions to enhance cellular uptake and deep tumor penetration. The MPEG-SS-PCL serves as a switch for drug release, which breaks under high glutathione (GSH) conditions within cells, facilitating intratumoral cellular drug release. Moreover, the cRGD peptide on cRGD-PEG-PCL acts as a navigator, targeting integrin α_v_β_3_ on tumor cells to achieve tumor accumulation and enhanced cellular uptake. Additionally, ARV-825 is selected as an enhancer for PDT and co-loaded with a photosensitizer in the hydrophobic core of the micelle system. To construct multifunctional ARV/Ce6@RDP micelles, we first evaluated the potential of the designed materials for the loading of ARV-825 and Ce6 through computer simulation. As shown in Fig. 1b, under normal physiological conditions, MPEG-SS-PCL and Ce6 or ARV-825 initially approached each other within 4 ns and embraced each other within 8–12 ns, followed by the encapsulation of Ce6 and ARV-825 into the hydrophobic core formed by MPEG-SS-PCL at 12 ns, finally assembling a stable drug-loaded spherical micelle within 16–20 ns. Thus, the co-loading of Ce6 and ARV-825 into MPEG-SS-PCL is theoretically feasible. Next, we synthesized MPEG-SS-PCL with reduction responsiveness, cRGD-PEG-PCL with targetability, and PCL-PEG-PEI-DM with charge-reversal ability, followed by identification of characteristic peaks of these polymers via proton nuclear magnetic resonance (^1^H NMR) (Supplementary Figs. 1–3).

The solvent evaporation-driven self-assembly method was used to prepare RDP, ARV@RDP, Ce6@RDP, and ARV/Ce6@RDP micelles, followed by dynamic light scattering (DLS) analysis. The results, depicted in Fig. 1c, d, revealed that the micelles had a particle size of approximately 100 nm and a narrow distribution, as well as a negative surface charge, which suggested the successful construction of micelles. Furthermore, the surface charge-switching capability of ARV/Ce6@RDP micelles was demonstrated by a change in surface charge from −9.1 mV to +7.0 mV after 4 h in solution at a pH of 6.8 (Fig. 1e). Transmission electron microscopy (TEM) images revealed that the ARV/Ce6@RDP micelles were homogeneous spheres under normal conditions or in a solution at pH 6.8, but disintegrated upon exposure to 10 mM GSH (Fig. 1f). Under high-GSH conditions, the cleavage of micelles signifies drug release, which is crucial for the efficacy and safety of drugs. The GSH response of the ARV/Ce6@RDP micelles was further confirmed, with a gradual increase in particle size as the time in a GSH (10 mM) solution (Fig. 1g). To characterize the loading of Ce6 in the micelles, UV/vis spectra of Ce6@RDP and ARV/Ce6@RDP were obtained. The results revealed that Ce6@RDP and ARV/Ce6@RDP had the same characteristic absorption peaks as free Ce6 (specifically, a broad peak at 660 nm), whereas RDP and ARV@RDP did not have any absorption at 660 nm, indicating the successful encapsulation of Ce6 in the RDP micelles and the lack of impact on UV absorption by the co-loading of ARV-825 (Fig. 1h). In addition, to visualize the co-loading capacity of RDP micelles on ARV-825 and Ce6, coumarin 6 (C6, a hydrophobic fluorescent dye used as a substitute for ARV-825) and Ce6 were simultaneously encapsulated into RDP micelles, followed by treatment of the cells. As demonstrated by a high-content imaging (HCI) system, the red fluorescence of Ce6 colocalized with the green fluorescence of C6, suggesting the successful co-delivery of two hydrophobic drugs by RDP micelles into the cells (Supplementary Fig. 4).

ARV/Ce6@RDP revealed a low critical micelle concentration (CMC) (Supplementary Fig. 5), indicating excellent stability following dilution in blood after intravenous administration. Furthermore, the ARV/Ce6@RDP micelles were stabilized in PBS solution and culture medium containing FBS, as evidenced by only a slight change in particle size over a 24-h period at 37 °C (Supplementary Fig. 6a). The ARV/Ce6@RDP micelles also displayed prolonged stability, with negligible changes in the particle size and dispersion coefficient within 50 days at room temperature (Supplementary Fig. 6b). Additionally, the hemolysis rate induced by ARV/Ce6@RDP micelles was measured to be 2.22%, falling below the established safe hemolysis rate threshold of 5% for biomaterials, which suggested the favorable blood compatibility of ARV/Ce6@RDP micelles (Fig. 1i). These findings underscore the suitability of the final constructed micelles for intravenous administration. Next, the light-generated ^1^O_2_ of ARV/Ce6@RDP was verified via the singlet oxygen probe DPBF. The results indicated that both Ce6@RDP and ARV/Ce6@RDP possessed comparable ^1^O_2_ generation capabilities to free Ce6 in vitro (Fig. 1j).

### Intracellular uptake and tumor accumulation of micelles

**Fig. 2:**
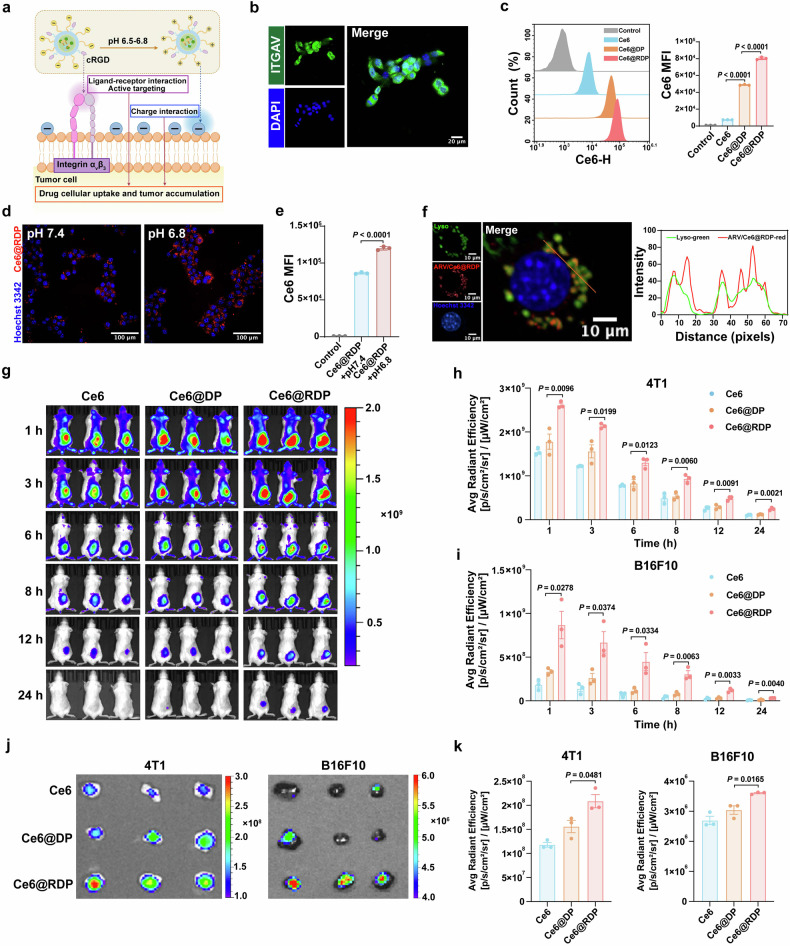
Cellular uptake and tumor accumulation of micelles. **a** Schematic representation of enhanced cellular uptake by RDP micelles. The figure was created with Figdraw.com. **b** Confocal immunofluorescence image of ITGAV proteins in 4T1 cells (blue: nucleus, green: ITGAV proteins). Scale bar: 20 µm. **c** Cellular uptake of free Ce6, Ce6@DP, and Ce6@RDP in 4T1 cells was measured via flow cytometry (*n* = 3 per group, two-tailed unpaired Student’s *t*-test). **d** Qualitative uptake images by HCI in 4T1 cells following incubation with Ce6@RDP for 4 h under different pH conditions (blue: nucleus, red: Ce6@RDP). Scale bar: 100 µm. **e** Quantitative uptake analysis by flow cytometry in 4T1 cells following incubation with Ce6@RDP for 4 h under different pH conditions (*n* = 3 per group, two-tailed unpaired Student’s *t*-test). **f** HCI images of ARV/Ce6@RDP and lysosome colocalization in 4T1 cells (blue: nucleus; green: lysosome; red: ARV/Ce6@RDP). Scale bar: 10 µm. The orange line indicates the region used for line-scan analysis, and the corresponding fluorescence intensity profiles show colocalization of ARV/Ce6@RDP with lysosomes. **g** In vivo fluorescence images of 4T1 tumor-bearing mice at 1, 3, 6, 8, 12, and 24 h post-injection of free Ce6, Ce6@DP, or Ce6@RDP. **h** Quantitative analysis of the real-time fluorescence signal at the 4T1 tumor site (*n* = 3 per group, two-tailed unpaired Student’s *t*-test). **i** Quantitative analysis of the real-time fluorescence signal at the B16F10 tumor site (*n* = 3 per group, two-tailed unpaired Student’s *t*-test). **j** In vitro fluorescence imaging of 4T1 and B16F10 tumor tissues 24 h after injection. **k** In vitro fluorescence quantitative analysis of 4T1 and B16F10 tumor tissues 24 h after injection (*n* = 3 per group, two-tailed unpaired Student’s *t*-test). The data are presented as the means ± SDs for in vitro experiments and means ± SEMs for in vivo experiments

Integrin α_v_β_3_ serves as a potential target for targeted drug delivery to various tumors on the basis of its specific interaction with cRGD peptides. As shown in Fig. 2a, the programmed delivery micelles were designed to employ cRGD peptides to target integrin α_v_β_3_ on tumor cell membranes, coupled with acid-triggered charge flipping to generate positive charges for interaction with negative charges on the cell membrane, resulting in enhanced drug cellular uptake. We first investigated integrin α_v_β_3_ expression in breast cancer cells (4T1 and MDA-MB-231) and melanoma cells (B16F10 and A375) via RT‒qPCR and immunofluorescence staining, and the results revealed that all four types of cells expressed integrin α_v_ and β_3_ (Fig. 2b and Supplementary Fig. 7). To explore the targetability of the prepared micelles, flow cytometry was used to analyze the intracellular uptake of free Ce6, non-targeted Ce6@DP (without cRGD peptide modification), and targeted Ce6@RDP. As shown in Fig. 2c and Supplementary Fig. 8a, the mean fluorescence intensity (MFI) of Ce6@RDP was greater than that of Ce6@DP, and Ce6@DP presented a greater MFI than free Ce6 did, indicating that micelles play an important role in enhancing the intracellular uptake of Ce6 and that the modification of the cRGD peptide further improved the cellular accumulation of drugs to precisely exert therapeutic effects. In addition, a decrease in the mean fluorescence intensity was observed after blocking integrin α_v_β_3_ with the cRGD peptide (Supplementary Fig. 8b), which provided additional evidence that the internalization of Ce6@RDP could be facilitated by the endocytosis pathway mediated by the cRGD peptide ligand. The results of further HCI were consistent with those of flow analysis (Supplementary Fig. 8c). The cellular uptake of Ce6@RDP in pH 7.4 or pH 6.8 solutions was subsequently qualitatively analyzed by HCI, and the results revealed a greater presence of Ce6 with red fluorescence in the culture medium at pH 6.8 (Fig. 2d). The same trend was found in quantitative analysis using flow cytometry (Fig. 2e), revealing that the reversal of charges from negative to positive for Ce6@RDP at pH 6.8 was beneficial for effective cellular uptake and further enhanced drug accumulation. To determine the intracellular localization of ARV/Ce6@RDP, colocalization analysis of lysosomes and micelles was performed via HCI. HCI images revealed that the green fluorescence signal of lysosomes and the red fluorescence signal of Ce6 in ARV/Ce6@RDP colocalized in the cytoplasm (Fig. 2f).

On the basis of the excellent cellular uptake of Ce6@RDP micelles in vitro, we investigated the tumor accumulation of Ce6@DP and Ce6@RDP micelles in 4T1 and B16F10 tumor-bearing mice. Live in vivo imaging revealed that, compared with Ce6@DP, Ce6@RDP exhibited superior tumor accumulation upon intravenous administration (Fig. 2g–i and Supplementary Fig. 9a), indicating that the cRGD peptide modification of micelles facilitated the accumulation of therapeutic agents at the tumor site and exhibited favorable in vivo targeting properties. Twenty-four hours after injection, the main organs and tumors of the mice were collected for the purpose of detecting fluorescence intensity. The results revealed that Ce6@RDP exhibited the strongest tumor region enrichment (Fig. 2j, k), which aligns with the in vivo imaging results. The designed RDP delivery system exhibited satisfactory drug accumulation, indicating its potential to maximize the therapeutic effects of the drug at the tumor site. Furthermore, compared with other organs, the liver achieved a greater degree of fluorescence enrichment (Supplementary Fig. 9b, c), suggesting that Ce6 may be metabolized primarily by the liver.

### Antitumor effect of ARV/Ce6@RDP in vitro

**Fig. 3:**
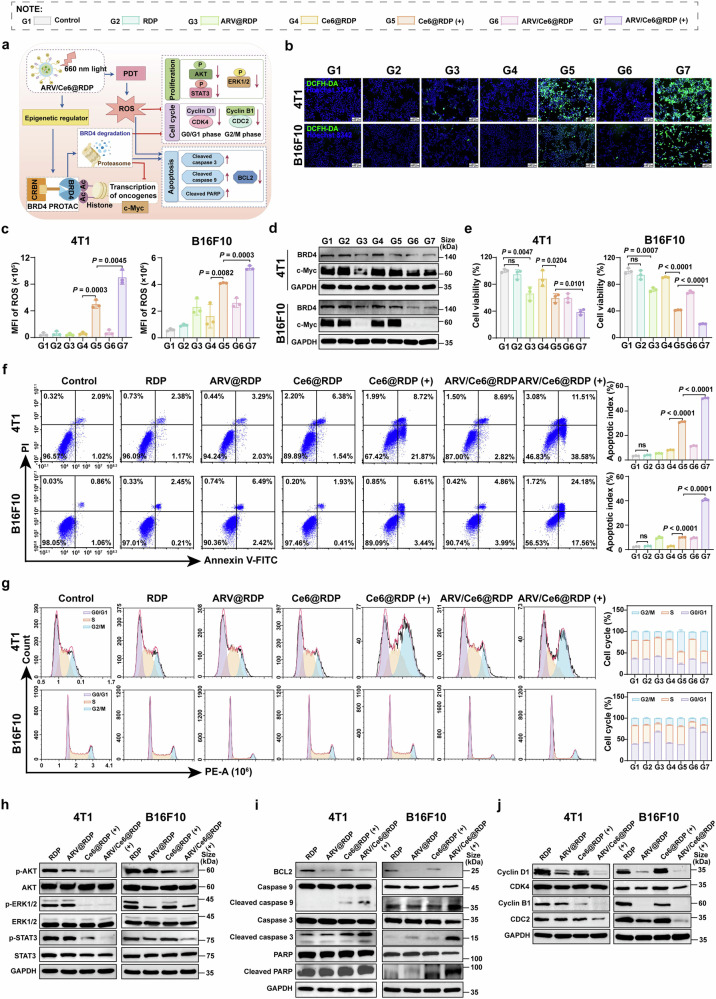
In vitro antitumor effects of ARV/Ce6@RDP on cell proliferation, apoptosis, and the cell cycle. **a** Schematic of the antitumor mechanism in cells via PDT and BRD4 PROTAC mediated by ARV/Ce6@RDP. The figure was created with Figdraw.com. **b** Representative images of ROS production in 4T1 and B16F10 cells subjected to different treatments (blue: nucleus; green: DCFH-DA-labeled ROS). Scale bar: 100 µm. **c** Flow cytometric analyses of ROS production in 4T1 and B16F10 cells after various treatments (*n* = 3 per group, two-tailed unpaired Student’s *t*-test). **d** Western blot analysis of BRD4 and c-Myc protein expression in 4T1 and B16F10 cells after various treatments. **e** Viability of 4T1 and B16F10 cells following different treatments (*n* = 3 per group, two-tailed unpaired Student’s *t*-test). **f** Flow cytometric analysis of apoptosis in 4T1 and B16F10 cells after different treatments by Annexin V-FITC/PI double staining (*n* = 3 per group, two-tailed unpaired Student’s *t*-test). **g** Flow cytometric analysis of the cell cycle in 4T1 and B16F10 cells receiving different treatments by PI staining (*n* = 3 per group). **h** Changes in p-AKT, AKT, p-ERK1/2, ERK1/2, p-STAT3, and STAT3 protein levels in 4T1 and B16F10 cells following various treatments. **i** Changes in the protein levels of genes involved in the apoptosis pathway after various treatments in 4T1 and B16F10 cells. **j** Changes in the protein levels associated with the cell cycle in 4T1 and B16F10 cells following various treatments. All the data in this figure are presented as the means ± SDs

Both epigenetic modifications and ROS play pivotal roles in various biological processes. The mechanism underlying ARV/Ce6@RDP-mediated precision targeting of tumor progression, particularly cell proliferation, the cell cycle, and apoptosis, was further investigated in vitro (Fig. 3a). First, the time of micelle incubation before laser irradiation and the optimal ratio of ARV-825 combined with Ce6 were confirmed by cellular uptake and cell cytotoxicity assays. The results showed that for four cell lines, the MFI of Ce6@RDP reached a maximum value within 8–24 h (Supplementary Fig. 10), and ARV-825 combined with PDT exhibited superior cytotoxicity to ARV-825 or PDT alone at a ratio of 1:5 (Supplementary Fig. 11). Therefore, ARV-825 and Ce6 were co-loaded onto RDP micelles at a ratio of 1:5 to incubate tumor cells for 8 h, followed by laser irradiation. The laser-induced increase in intracellular ROS in ARV/Ce6@RDP was subsequently detected with a DCFH-DA probe. Compared with the groups without laser irradiation, the Ce6@RDP and ARV/Ce6@RDP groups with laser irradiation presented significant increases in intracellular ROS levels (Fig. 3b and Supplementary Fig. 12a). Moreover, flow cytometry analysis revealed that, compared with Ce6@RDP, ARV/Ce6@RDP resulted in greater ROS generation post-laser irradiation in 4T1, B16F10 and A375 cells, suggesting that the co-loading of ARV-825 increased the amount of ROS production induced by Ce6 (Fig. 3c and Supplementary Fig. 12b). This effect may be related to BRD4 inhibition, which induces lipid ROS generation and reduces ROS elimination.^30,31^ Next, with the BRD4 PROTAC ARV-825 as another antitumor agent in ARV/Ce6@RDP micelles, we examined the impact of ARV/Ce6@RDP micelles on BRD4 protein degradation in tumor cells via western blotting. The results revealed lower levels of BRD4 proteins in the ARV@RDP, ARV/Ce6@RDP, and ARV/Ce6@RDP (+) groups, which were consistent with the significant downregulation of the oncogenic protein c-Myc regulated by BRD4 (Fig. 3d and Supplementary Fig. 12c).

The MTT assay revealed that the ARV-825 and Ce6 co-loaded micelles had greater tumor-killing effects than the micelles loaded solely with ARV-825 or Ce6 under 660 nm irradiation (Fig. 3e and Supplementary Fig. 12d). In addition, the degree of apoptosis induced by ARV/Ce6@RDP was determined via Annexin V-FITC/PI staining. As shown in Fig. 3f and Supplementary Fig. 13a, a greater number of apoptotic cells were observed in 4T1 cells treated with ARV/Ce6@RDP (+) than in those treated with Ce6@RDP (+) or ARV@RDP alone, and a consistent trend was found in the other three types of tumor cells, suggesting the advantage of ARV/Ce6@RDP micelles with laser irradiation in inducing apoptosis. To further examine the antitumor effectiveness of ARV/Ce6@RDP micelles, flow cytometry analysis of the cell cycle was performed. The results depicted in Fig. 3g revealed a notable increase in the distribution of G2/M phase cells in 4T1 cells following treatment with Ce6@RDP (+) or ARV/Ce6@RDP (+), whereas a significant increase in G0/G1 phase cells was observed in B16F10 cells treated with ARV@RDP or ARV/Ce6@RDP (+). This phenomenon could be attributed to the ability of ARV/Ce6@RDP (+) to induce unique cell cycle arrest in various cell types. Interestingly, there was also a different cell cycle arrest in human tumor cells, with ARV/Ce6@RDP (+) treatment inducing G2/M phase arrest in MDA-MB-231 cells and G0/G1 phase arrest in A375 cells (Supplementary Fig. 13b). These findings collectively demonstrated the potential efficacy of ARV/Ce6@RDP micelles in combating tumor growth.

Next, the collaborative mechanism of Ce6 and ARV-825 in RDP micelles was explored via western blotting. As shown in Fig. 3h and Supplementary Fig. 14a, ARV/Ce6@RDP (+) effectively suppressed the phosphorylation of multiple signaling pathway proteins (AKT, ERK1/2, and STAT3 proteins), suggesting that the synergistic antitumor effects of Ce6 and ARV-825 might be the result of various biological responses. On the basis of the significant apoptosis induced by ARV/Ce6@RDP (+) according to flow cytometry analysis, further apoptosis protein analysis (Fig. 3i and Supplementary Fig. 14b) demonstrated that ARV/Ce6@RDP (+) triggered apoptosis through the intrinsic pathway, as evidenced by the downregulation of BCL2 and the activation of Caspase 9, Caspase 3, and PARP. In addition, as shown in Fig. 3j and Supplementary Fig. 14c, ARV/Ce6@RDP (+) not only downregulated G0/G1 phase proteins (Cyclin D1 and CDK4) in four types of cells but also downregulated G2/M phase proteins (Cyclin B1 and CDC2), which might be attributed to the combined action of PDT and ARV-825. Moreover, different inhibitory effects on proteins associated with the G0/G1 phase or G2/M phase were observed in different cells, which also explained the different cycle arrest periods displayed in various tumor cells by flow cytometry analysis. This may be attributed to the synergistic effects of PDT and ARV-825 on cell cycle distribution and arrest, and the outcome of this interaction may depend on which agent exerts a stronger influence on the cell cycle in a given cell type.

### Immune activation of ARV/Ce6@RDP in vitro

**Fig. 4:**
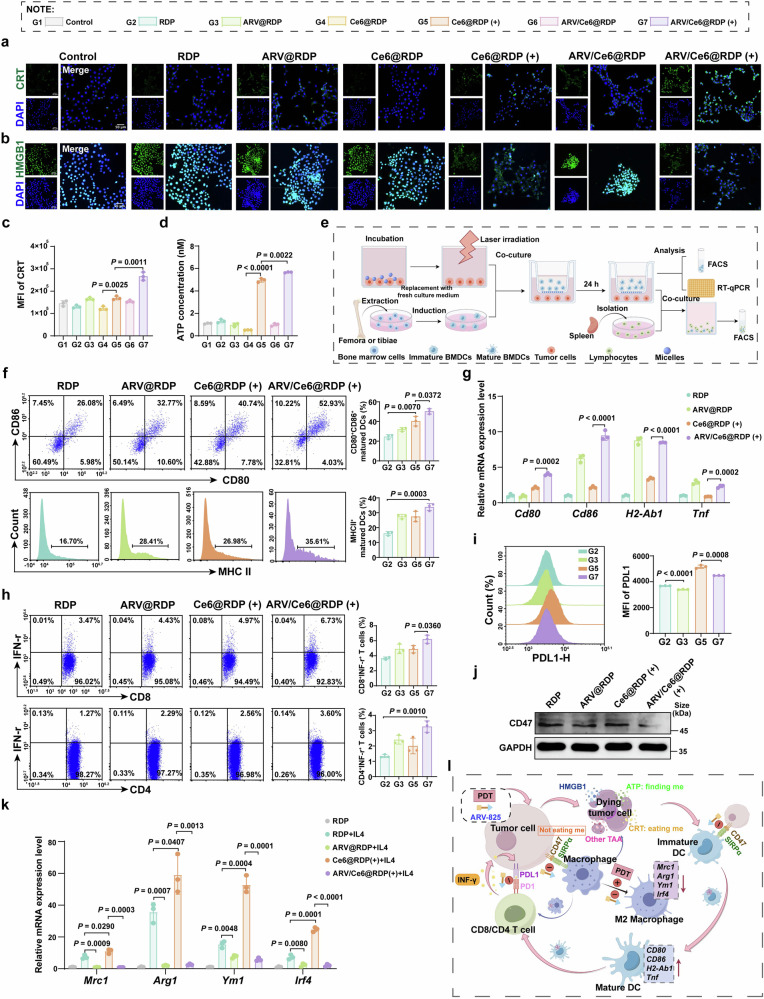
In vitro immune activation induced by ARV/Ce6@RDP micelle treatment. **a** CLSM images of CRT exposure in 4T1 cells subjected to various treatments (blue: nucleus; green: CRT). Scale bar: 50 µm. **b** CLSM imaging of HMGB1 expression in 4T1 cells after different treatments (blue: nucleus, green: HMGB1). Scale bar: 50 µm. **c** Quantitative flow cytometry analysis of CRT exposure in 4T1 cells after different treatments (*n* = 3 per group, two-tailed unpaired Student’s *t*-test). **d** Detection of ATP release from 4T1 cells after different treatments (n = 3 per group, two-tailed unpaired Student’s *t*-test). **e** Schematic illustration of BMDC maturation in a transwell system and in vitro mature DCs promoting T-cell activation. The figure was created with Figdraw.com. **f** Flow cytometric analyses of mature DCs (CD11c^+^CD80^+^CD86^+^ and CD11c^+^MHCII^+^) after incubation with 4T1 cells receiving various treatments (*n* = 3 per group, two-tailed unpaired Student’s *t*-test). **g** *Cd80*, *Cd86*, *H2-Ab1*, and *Tnf* gene expression in BMDCs incubated with 4T1 cells receiving various treatments determined by qPCR (*n* = 3 per group, two-tailed unpaired Student’s *t*-test). **h** Flow cytometric analyses of T-cell activation (CD3^+^CD8^+^IFN-γ^+^ and CD3^+^CD4^+^IFN-γ^+^) following coincubation with mature DCs induced by 4T1 cells receiving various treatments (*n* = 3 per group, two-tailed unpaired Student’s *t*-test). **i** Flow cytometric analyses for PDL1 expression in 4T1 cells treated with RDP, ARV@RDP, Ce6@RDP (+) and ARV/Ce6@RDP (+) (*n* = 3 per group, two-tailed unpaired Student’s *t*-test). **j** Western blot analysis of CD47 expression in 4T1 cells following various treatments. **k** Changes in M2 macrophage-related gene expression in BMDMs from BALB/c mice after different treatments (*n* = 3 per group, two-tailed unpaired Student’s *t*-test). **l** Schematic illustration of the antitumor immune activation of ARV/Ce6@RDP in vitro. The figure was created with Figdraw.com. All the data in this figure are presented as the means ± SDs

To explore the immune response triggered by ARV/Ce6@RDP therapy, the release of damage-associated molecular patterns (DAMPs) triggered by PDT-induced immunogenic cell death (ICD) was first assessed. Confocal images revealed that ARV/Ce6@RDP (+) induced both calreticulin (CRT) exposure and high mobility group box 1 (HMGB1) leakage in 4T1 cells (Fig. 4a, b) and B16F10 cells (Supplementary Fig. 15a, b). Flow cytometry analysis further confirmed that, compared with Ce6@RDP (+) treatment, ARV/Ce6@RDP (+) treatment led to membrane translocation of CRT and increased exposure (Fig. 4c and Supplementary Fig. 15c), indicating that ARV-825 augmented the release of “eating me” signals induced by PDT. Consistent with these results, ARV-825 enhanced ATP release induced by PDT (Fig. 4d and Supplementary Fig. 15d), which suggested a stronger “finding me” signal in cells treated with ARV/Ce6@RDP (+), subsequently resulting in increased recruitment of antigen-presenting cells (APCs). The immune-stimulating effect induced by ICD mainly involves the recruitment and maturation of DCs, as well as the subsequent activation of T cells mediated by antigen presentation.^32^ As illustrated in Fig. 4e, BMDCs were seeded into the upper chamber of the transwell coculture system, followed by coincubation with tumor cells treated with various micelles in the lower chamber. After 24 h, flow cytometry analysis revealed that, compared with Ce6@RDP (+) alone, ARV/Ce6@RDP (+) induced greater DC maturation (CD11c^+^CD80^+^CD86^+^ and CD11c^+^MHCII^+^) (Fig. 4f and Supplementary Fig. 16), which might be attributed to the ARV-825-enhanced ICD effect and release of tumor antigens. Moreover, after coincubation, the BMDCs were collected to detect DC maturation–related gene expression. The RT‒qPCR results (Fig. 4g) revealed that the BMDCs in the ARV/Ce6@RDP (+) groups expressed higher levels of the *Cd80*, *Cd86*, *H2-Ab1*, and *Tnf* genes than those in the Ce6@RDP (+) groups, indicating that ARV-825 in combination with PDT more effectively promoted DC maturation. We subsequently evaluated the activation of CD8^+^ T cells stimulated by mature DCs triggered with nanomedicines. Flow cytometry analysis revealed that mature DCs induced by ARV/Ce6@RDP (+)-treated 4T1 cells increased the numbers of CD3^+^CD8^+^IFN-γ^+^ T cells and CD3^+^CD4^+^IFN-γ^+^ T cells (Fig. 4h). The same trend was observed in B16F10 cells (Supplementary Fig. 17a), suggesting that combination therapy with ARV-825 and PDT was more conducive to the activation of T cells.

IFN-γ can induce PDL1 expression in tumor cells, leading to immune escape through the interaction of PDL1 with programmed cell death receptor 1 (PD1) expressed on T cells.^33^ Further flow cytometry detection revealed that compared with Ce6@RDP (+) treatment, ARV/Ce6@RDP (+) treatment could reduce the number of PDL1-positive 4T1 and B16F10 cells (Fig. 4i and Supplementary Fig. 17b). Moreover, the same trend was observed via western blot analysis (Supplementary Fig. 18), indicating that ARV-825 has the ability to improve immune tolerance induced by PDT. On the basis of the ICD-DC-T-cell activation effects and the downregulation of PDL1 expression induced by the combined treatment of ARV-825 and PDT, together with previous reports showing that PDT and BRD4 inhibitors can upregulate MHC-I expression on tumor cells,^34,35^ we hypothesize that the combination of PDT and ARV-825 may alter the proportion of CD8⁺ T cells within the tumor microenvironment by modulating immune checkpoints and antigen presentation pathways. Consistent with this, in a coculture system mimicking the interaction between tumor cells and lymphocytes within the tumor microenvironment, a slight increase in the proportion of CD8⁺ T cells was observed following coincubation with tumor cells treated with Ce6@RDP (+), and the ARV/Ce6@RDP (+) group presented a greater number of CD8^+^ T cells than the Ce6@RDP (+) group (Supplementary Fig. 19), which implied that ARV-825 might potentiate the immune activation triggered by PDT.

Additionally, our study revealed that ARV-825 could downregulate CD47 protein expression in tumor cells (Fig. 4j and Supplementary Fig. 20). The combination of CD47-SIRPα can inhibit phagocytosis by myeloid cells on tumor cells, thereby hindering the presentation of tumor antigens and the activation of T cells.^36^ Next, the phagocytosis of macrophages and DCs by tumor cells treated with various nanomedicines was investigated. CLSM images revealed that ARV/Ce6@RDP (+) treatment resulted in an increase in the engulfment of green fluorescence-labeled tumor cells by BMDMs labeled with red fluorescence (Supplementary Fig. 21). Consistently, as shown in Supplementary Fig. 22, compared with that in the other treatments, a notably increased CFSE signal in BMDMs (CD11b^+^F4/80^+^) and BMDCs (CD11c^+^) was observed in the ARV/Ce6@RDP (+) group. These findings suggested that tumor cells treated with ARV/Ce6@RDP (+) were more likely to be phagocytized by BMDCs and BMDMs, which might be attributed to increased tumor antigens and CD47 inhibition. Furthermore, flow cytometric analysis of macrophage polarization revealed that ARV-825@RDP inhibited macrophage M2 polarization (Supplementary Fig. 23), which is consistent with our previous findings.^19^ Surprisingly, Ce6@RDP (+) promoted M2 polarization of macrophages, but ARV/Ce6@RDP (+) suppressed this effect. This trend was further validated by RT‒qPCR analyses, which revealed a greater decrease in the expression of genes associated with M2 macrophage polarization in the ARV/Ce6@RDP (+) group than in the Ce6@RDP (+) group (Fig. 4k), suggesting that ARV-825 could enhance PDT by inhibiting M2 macrophage polarization and further reversing the immunosuppressive microenvironment. Based on these findings, for clarity, the immune activation of ARV/Ce6@RDP in vitro is outlined in Fig. 4l.

### Antitumor effects of ARV/Ce6@RDP in breast cancer xenograft and recurrence mouse models

**Fig. 5:**
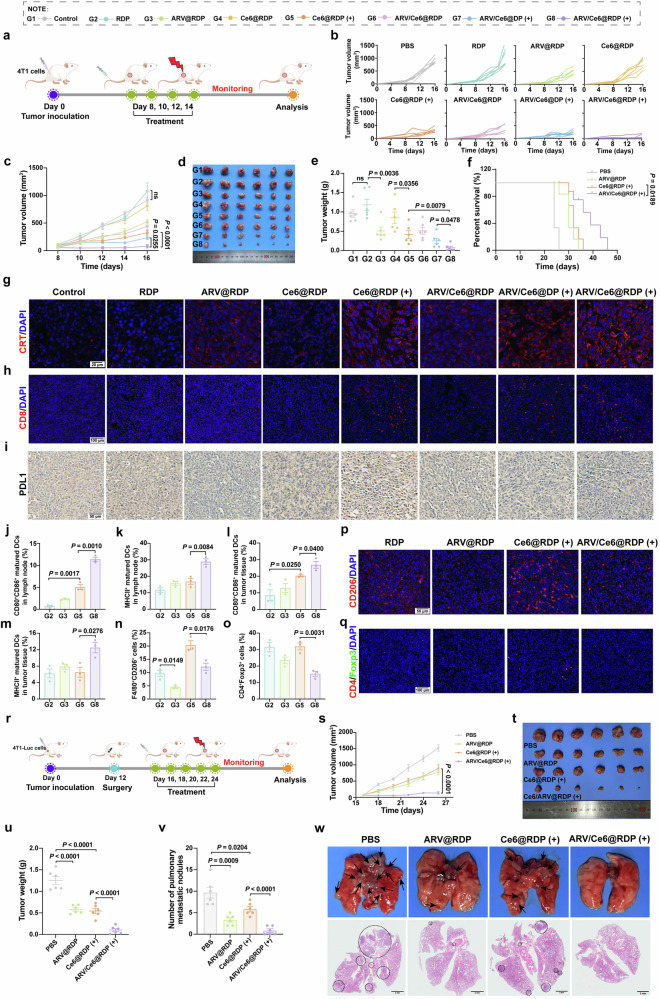
In vivo outcomes of antitumor efficacy, immune activation, and postsurgical recurrence and metastasis by ARV/Ce6@RDP micelles in a 4T1 tumor model in BALB/c mice. **a** Schematic illustration of the experimental procedure used to evaluate the inhibitory effect on subcutaneous 4T1 tumor growth. The figure was created with Figdraw.com. **b** Tumor growth curves of individual mice in different groups. **c** Average tumor growth curves of the different groups. (*n* = 6 per group; two-way ANOVA with Tukey’s multiple comparisons test). **d** Picture of tumors in different groups after treatment. **e** Tumor weight analysis of various groups at the end of the experiment (*n* = 6 per group, two-tailed unpaired Student’s *t*-test). **f** Survival percentages of the mice treated with PBS, ARV@RDP, Ce6@RDP (+), or ARV/Ce6@RDP (+) (*n* = 6 per group, log-rank test). **g** Representative images of CRT protein immunofluorescence staining of tumor tissues. Scale bar: 20 µm. **h** Fluorescence images of tumor sections showing infiltrated CD8^+^ T cells. Scale bar: 100 µm. **i** Representative image of PDL1 expression in tumor sections from different treatment groups. Scale bar: 50 µm. **j**, **k** Flow cytometry quantitative analysis of CD11c^+^CD80^+^CD86^+^ mature DCs (**j**) and CD11c^+^MHCII^+^ mature DCs (**k**) in tumor-draining lymph nodes (*n* = 3 per group; two-tailed unpaired Student’s *t*-test). Flow cytometry quantitative analysis of CD11c^+^CD80^+^CD86^+^ mature DCs (**l**), CD11c^+^MHCII^+^ mature DCs (**m**), CD11b^+^F4/80^+^CD206^+^ macrophages (**n**), and CD3^+^CD4^+^Foxp3^+^ T cells (**o**) gated on L/D^−^CD45^+^ cells in tumor tissues (*n* = 3 per group, two-tailed unpaired Student’s *t*-test). Representative IF staining of CD206^+^ M2 macrophages (**p**, scale bar: 50 µm) and CD4^+^Foxp3^+^ Treg cells (**q**, scale bar: 100 µm) in tumor tissues after various treatments. **r** Schematic illustration of the animal experimental design for postsurgical recurrence and metastasis. The figure was created with Figdraw.com. **s** Recurrence-related tumor volume curves of the different groups. (*n* = 6 per group; two-way ANOVA with Tukey’s multiple comparisons test). Recurrent tumor images (**t**) and quantification of tumor weights (**u**) in different groups after the experiment (*n* = 6 per group, two-tailed unpaired Student’s *t*-test). **v** Quantification of pulmonary metastasis nodules in various groups (*n* = 6 per group, two-tailed unpaired Student’s *t*-test). **w** Representative photographs and H&E staining of lung tissues from mice receiving different treatments. Scale bar: 2 mm. All data in this figure are presented as the means ± SEMs

Motivated by the favorable performance of ARV/Ce6@RDP (+) therapy in vitro, we investigated the in vivo therapeutic efficacy of ARV/Ce6@RDP (+). First, a 4T1 breast cancer xenograft model was established by subcutaneously injecting 4T1 cells into the back right flank of the mice, and the treatment was performed four times, followed by monitoring changes in tumor volume, as shown in the scheme of mice treatment (Fig. 5a). The results showed a notable suppression of tumor growth in the ARV/Ce6@RDP (+) treatment group, with a more pronounced inhibitory effect than that of the Ce6@RDP (+) and ARV/Ce6@DP (+) groups (Fig. 5b–e), demonstrating that, on the basis of the targeted delivery system, ARV-825 significantly enhanced the antitumor effect of PDT. During the treatment period, there was no significant change in the body weight of the mice in each group (Supplementary Fig. 24), indicating the favorable biosafety of ARV/Ce6@RDP micelle treatment. In addition, a survival benefit was observed with the combination treatment in the ARV/Ce6@RDP (+) group (Fig. 5f). These results suggest that ARV-825 enhances the antitumor effect of PDT and the advantages of targeted delivery strategies, which is in agreement with the in vitro effects.

To understand the antitumor mechanism of ARV/Ce6@RDP micelles in vivo, cell proliferation, angiogenesis, and apoptosis in tumor tissue after various treatments were preliminarily explored. Immunohistochemical images displayed that monotherapy with ARV@RDP or Ce6@RDP (+) inhibited tumor cell proliferation and angiogenesis, but the effect was weaker than that of ARV/Ce6@RDP (+) (Supplementary Figs. 25 and 26). TUNEL staining revealed that, compared with Ce6@RDP (+) treatment, ARV/Ce6@RDP (+) treatment significantly increased tumor cell apoptosis (Supplementary Fig. 27). In addition, HE staining of organs showed that there were no discernible pathological alterations among the groups after treatment (Supplementary Fig. 28). Blood biochemical tests revealed that ARV/Ce6@RDP (+) treatment, like other treatments, did not result in significant changes in liver, kidney, blood glucose, blood lipid or myocardial enzyme levels (Supplementary Fig. 29), which further supported the conclusion that ARV/Ce6@RDP exhibited favorable biosafety characteristics.

In addition to the direct killing effect, the therapeutic effect of ARV/Ce6@RDP against tumors may also be attributed to the activation of the immune system induced by ARV/Ce6@RDP. To further investigate the immune responses triggered by ARV/Ce6@RDP in vivo, the tumors and lymph nodes of the mice were collected for analysis of relevant indices. The CRT protein in the tumors was detected via immunofluorescence, and enhanced CRT red fluorescence was observed in the groups subjected to laser irradiation (Fig. 5g), suggesting the activation of ICD in vivo. Furthermore, consistent with these results, the number of CD8^+^ T cells increased after laser exposure (Fig. 5h). PDT stimulates the release of IFN-γ from CD8^+^ T cells to kill tumor cells, whereas IFN-γ can upregulate PDL1 expression, which in turn inhibits the cytotoxic killing ability of T cells. Immunohistochemical staining for the PDL1 protein in tumor tissues also revealed upregulated PDL1 after Ce6@RDP (+) treatment, but this induction was attenuated when PDT was combined with ARV-825 (Fig. 5i). Further flow cytometry analysis revealed that the number of mature DCs in the lymph nodes and tumor tissues significantly increased in the mice that received ARV/Ce6@RDP (+) treatment (Fig. 5j–m and Supplementary Fig. 30a, b). In addition, compared with Ce6@RDP (+) treatment, ARV/Ce6@RDP (+) treatment effectively reduced the populations of M2 macrophages (L/D^−^CD45^+^CD11b^+^F4/80^+^CD206^+^) (Fig. 5n and Supplementary Fig. 30c). Consistently, ARV/Ce6@RDP (+) treatment also resulted in significantly fewer Treg cells (L/D^−^CD45^+^CD3^+^CD4^+^Foxp3^+^) in tumors (Fig. 5o and Supplementary Fig. 30d). These data revealed that ARV/Ce6@RDP (+) treatment inhibited tumor growth by limiting the function of M2 macrophages and Treg cells. To further confirm these results, we further examined M2 macrophages and Treg cells in tumor tissues via immunofluorescence staining. The fluorescence images showed a consistent trend with the above flow analysis (Fig. 5p, q). Taken together, these findings indicate that the antitumor immune response could be effectively activated by ARV/Ce6@RDP (+) in vivo, which could simultaneously improve the immunosuppressive microenvironment. Studies have shown that BRD4 inhibitors can stimulate NK cell activation by downregulating the expression of NK inhibitory receptors.^37^ Moreover, NK cells play an important role in the immune killing of tumors. To investigate whether the prepared ARV/Ce6@RDP micelles can affect NK cells in the tumor microenvironment, the content of activated NK cells in tumor tissues was analyzed. Interestingly, the proportion of activated NK cells (L/D^−^CD45^+^CD3^−^CD49^+^CD107a^+^) in the tumors of mice treated with ARV/Ce6@RDP (+) slightly increased compared with that in the tumors of those treated with Ce6@RDP (+) (Supplementary Fig. 31), suggesting that ARV-825 combined with PDT could drive the activation of NK cells and further support the antitumor immune effect of ARV/Ce6@RDP (+) treatment.

Postsurgical tumor recurrence and metastasis frequently result in the failure of cancer treatment. The notable effectiveness of ARV/Ce6@RDP against tumors in vitro and in vivo prompted us to investigate whether ARV/Ce6@RDP could prevent postoperative tumor recurrence and metastasis. The breast cancer model was established by subcutaneous implantation of 4T1-Luc cells into the back right flank of the mice (Fig. 5r). The tumor was excised via surgical resection when the tumor volume reached approximately 300 mm^3^, and the surgically treated mice were subjected to various treatments on the fourth postoperative day. As shown in Fig. 5s–u, the mice in the PBS group exhibited swift tumor recurrence, and the mice in the ARV@RDP and Ce6@RDP (+) groups presented a moderate level of inhibition of tumor recurrence. In contrast, the combination treatment of ARV/Ce6@RDP (+) significantly inhibited tumor recurrence. Moreover, live imaging of the mice after surgery and treatment strongly inhibited tumor growth in the ARV/Ce6@RDP (+) treatment group (Supplementary Fig. 32). Further analysis of tumor lung metastasis after treatment revealed that ARV/Ce6@RDP (+) induced fewer lung metastasis nodules than the other treatments (Fig. 5v, w), suggesting that ARV/Ce6@RDP (+) treatment effectively suppressed spontaneous lung metastases of 4T1 tumors. In addition, there was no significant difference in the weight of each group of mice (Supplementary Fig. 33), indicating that ARV/Ce6@RDP was also safe for the treatment of mouse tumor recurrence and metastasis.

### In vivo anti-melanoma efficacy and immune activation by ARV/Ce6@RDP treatment

**Fig. 6:**
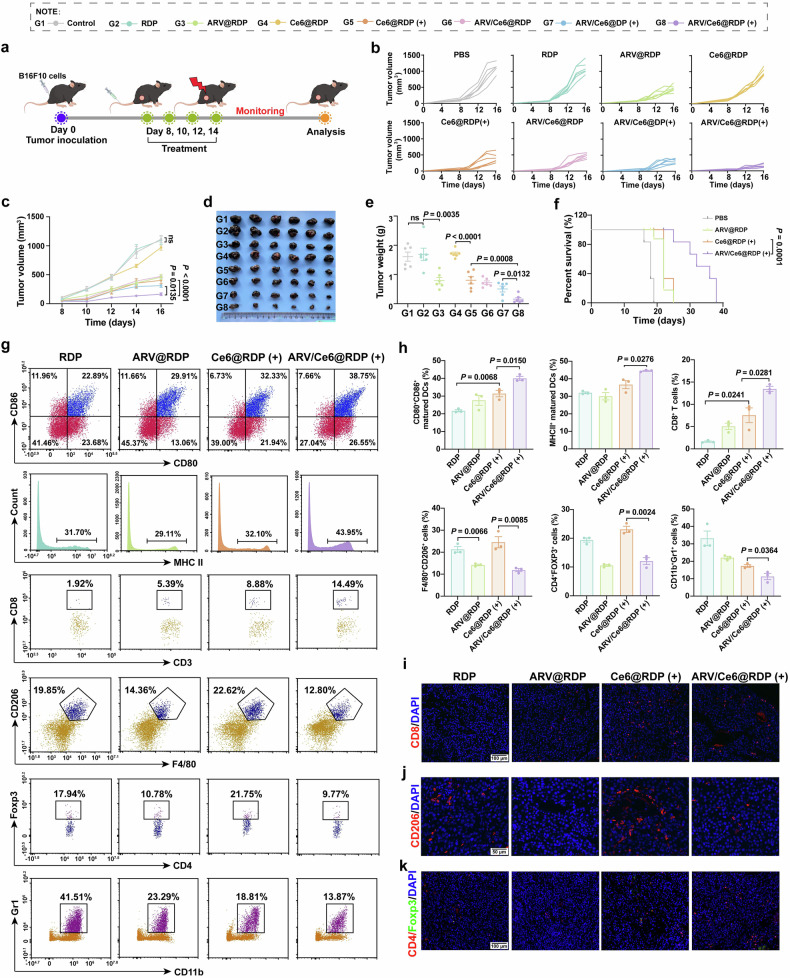
Treatment with ARV/Ce6@RDP led to enhanced therapeutic efficacy and immune response in melanoma. **a** Schematic illustration of the treatment design for the subcutaneous B16F10 tumor mouse model. The figure was created with Figdraw.com. **b** Individual tumor growth curves for B16F10 tumor-bearing mice in each treatment group. **c** Average tumor growth curve of B16F10 tumor-bearing mice in each treatment group (*n* = 6 per group, two-way ANOVA with Tukey’s multiple comparisons test). B16F10 tumor image (**d**) and tumor weight analysis (**e**) of each group at the end of the experiment (*n* = 6 per group, two-tailed unpaired Student’s *t*-test). **f** Survival curves of B16F10 tumor-bearing mice treated with the indicated formulations (*n* = 6 per group, log-rank test). Representative flow cytometry plots (**g**) and quantification analysis (**h**) of CD11c^+^CD80^+^CD86^+^ mature DCs in lymph nodes, CD11c^+^MHCII^+^ mature DCs in lymph nodes, L/D^−^CD45^+^CD3^+^CD8^+^ T cells in tumors, L/D^−^CD45^+^CD11b^+^F4/80^+^CD206^+^ macrophages in tumors, L/D^−^CD45^+^CD3^+^CD4^+^Foxp3^+^ Treg cells in tumors and L/D^−^CD45^+^CD11b^+^Gr1^+^ MDSCs in tumors from B16F10 tumor-bearing mice receiving different treatments (*n* = 3 per group, two-tailed unpaired Student’s *t*-test). **i**–**k** Representative IF images of tumor-infiltrated CD8^+^ T cells (scale bar: 100 µm), CD206^+^ M2 macrophages (scale bar: 50 µm), and CD4^+^Foxp3^+^ Treg cells (scale bar: 100 µm). All data in this figure are presented as the means ± SEMs

After verifying the antitumor activity of ARV/Ce6@RDP in breast cancer, we explored the therapeutic effect of ARV/Ce6@RDP on melanoma. First, using the same method as before, a subcutaneous melanoma model was established, followed by different treatments (Fig. 6a). The ability of ARV/Ce6@RDP to inhibit tumor growth was evaluated by monitoring the tumor volume and body weight every two days after the first treatment. As shown in Fig. 6b, c, co-delivery of Ce6 and ARV-825 via RDP micelles after laser irradiation led to more obvious inhibition of tumor growth than Ce6@RDP (+) treatment alone, and all the treatments were well tolerated, as evidenced by similar changes in body weight over time compared with those in the PBS group (Supplementary Fig. 34). A similar result was also demonstrated by the analysis of tumor weight after treatment. As depicted in Fig. 6d, e, the tumor weights of the mice in the targeted ARV/Ce6@RDP (+) group were lower than those in both the untargeted ARV/Ce6@DP (+) group and the single-treatment Ce6@RDP (+) group. Moreover, an obvious increase in survival was observed in the ARV/Ce6@RDP (+) treatment group (Fig. 6f). These results revealed that the constructed RDP micelle system for targeted delivery of Ce6 and ARV-825 in B16F10 melanoma treatment had significant antitumor effects, which is consistent with the results of 4T1 treatment. Compared with Ce6@RDP (+), ARV/Ce6@RDP (+) had a greater in vivo impact on cell proliferation inhibition, angiogenesis suppression, and apoptosis induction, as shown by Ki67, CD31, and TUNEL staining, respectively (Supplementary Figs. 35–37). In addition, H&E staining of organs and blood biochemical indices revealed no significant abnormalities among the groups (Supplementary Fig. 38 and 39), suggesting the satisfactory histocompatibility and systemic biosafety of the ARV/Ce6@RDP micelles.

To demonstrate the contributions of ARV-825 and PDT-triggered immunotherapy to the antitumor effects of ARV/Ce6@RDP in vivo, immune-related indicators were further analyzed after treatment. Like in breast cancer, ARV/Ce6@RDP (+) also significantly increased the fluorescence intensity of CRT on the cell surface in melanoma (Supplementary Fig. 40). Further flow cytometric analysis of immune cells (Fig. 6g, h and Supplementary Fig. 41) revealed that, compared with Ce6@RDP (+) treatment, ARV/Ce6@RDP (+) treatment led to an obvious improvement in the antitumor immune response, as evidenced by increases in both the total numbers of mature DCs (CD11c^+^CD80^+^CD86^+^ and CD11c^+^MHCII^+^) in the lymph nodes and the numbers of tumor-infiltrating cytotoxic T cells (L/D^−^CD45^+^CD3^+^CD8^+^) and reductions in the population of immunosuppressive cells, including M2 macrophages (L/D^−^CD45^+^CD11b^+^F4/80^+^CD206^+^), Treg cells (L/D^−^CD45^+^CD3^+^CD4^+^Foxp3^+^) and MDSCs (L/D^−^CD45^+^CD11b^+^Gr1^+^). This conclusion was further supported by the consistent results of immunofluorescence staining for tumor-infiltrated CD8^+^ T cells, CD206^+^ M2 macrophages, and CD4^+^Foxp3^+^ Treg cells in the different treatment groups (Fig. 6i-k), suggesting that ARV/Ce6@RDP (+) successfully reshaped the tumor microenvironment by inducing antitumor immune cells and reducing the number of tumor-infiltrating immunosuppressive cells.

### ARV-825 enhanced PDT by inhibiting *Ccl5* transcription

**Fig. 7:**
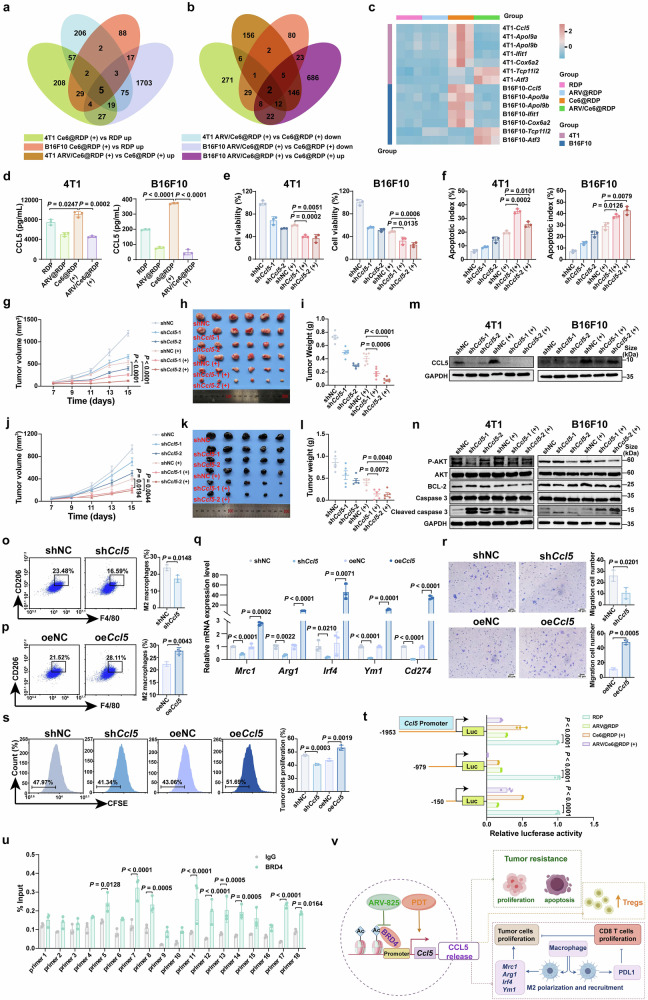
Mechanistic study of the antitumor effects of ARV/Ce6@RDP micelles. **a** Venn diagrams of the intersection between upregulated genes in Ce6@RDP (+) vs RDP and downregulated genes in ARV/Ce6@RDP (+) vs Ce6@RDP (+) for 4T1 and B16F10 cells based on RNA-seq analysis. **b** Venn diagrams of the intersection between upregulated genes in Ce6@RDP (+) vs RDP and upregulated genes in ARV/Ce6@RDP (+) vs Ce6@RDP (+) for 4T1 and B16F10 cells. **c** Heatmap of the intersection genes from (**a**) and (**b**) expressed in 4T1 and B16F10 cells receiving different treatments. **d** Quantification of the CCL5 level in the supernatants of treated 4T1 and B16F10 cells (*n* = 3 per group, two-tailed unpaired Student’s *t*-test). Cell viability (**e**) and apoptosis (**f**) analysis of *Ccl5*-knockdown 4T1 and B16F10 cells with and without treatment with PDT (*n* = 3 per group, two-tailed unpaired Student’s *t*-test). **g**–**n** Effects of *Ccl5* knockdown on PDT efficacy in vivo. Tumor growth curves (**g**, *n* = 6 per group, two-way ANOVA with Tukey’s multiple comparisons test), post-treatment tumor photographs (**h**), and tumor weight analysis (**i**, *n* = 6 per group, two-tailed unpaired Student’s *t*-test) of *Ccl5*-knockdown 4T1 cells and control cell xenografts treated with or without PDT; Tumor growth curves (**j**, *n* = 5 per group, two-way ANOVA with Tukey’s multiple comparisons test), post-treatment tumor photographs (**k**) and tumor weight analysis (**l**, *n* = 5 per group, two-tailed unpaired Student’s *t*-test) of *Ccl5*-knockdown B16F10 cells and control cell xenografts treated with or without PDT; Expression levels of the CCL5 protein (**m**) and proteins (**n**) related to proliferation and apoptosis in tumor tissues collected from *Ccl5*-knockdown 4T1 and B16F10 xenografts after treatment with or without PDT. **o** Flow cytometric analyses of M2 polarization in BMDMs after treatment with culture medium from *Ccl5*-knockdown 4T1 cells or control cells (*n* = 3 per group, two-tailed unpaired Student’s *t*-test). **p** M2 polarization analysis of BMDMs treated with culture medium from *Ccl5*-overexpressing 4T1 cells and control cells (*n* = 3 per group, two-tailed unpaired Student’s *t*-test). **q** *Mrc1*, *Arg1*, *Irf4*, *Ym1*, and *Cd274* gene expression in BMDMs incubated with culture medium from different 4T1 cells determined by qPCR (*n* = 3 per group, two-tailed unpaired Student’s *t*-test). **r** Recruitment of M2 macrophages by *Ccl5*-knockdown or *Ccl5*-overexpressing 4T1 cells (*n* = 3 per group; two-tailed unpaired Student’s *t*-test; scale bar: 50 µm). **s** CFSE staining analysis of 4T1 cell proliferation in BMDMs subjected to different treatments (*n* = 3 per group, two-tailed unpaired Student’s *t*-test). **t** Effects of nanomedicines on *Ccl5* promoter activity detected by dual-luciferase assay (*n* = 3 per group, two-tailed unpaired Student’s *t*-test). **u** ChIP‒qPCR analysis of BRD4 protein binding to the *Ccl5* promoter in B16F10 cells (*n* = 3 per group; two-way ANOVA with Sidak’s multiple comparisons test). **v** Schematic diagram of ARV-825-mediated inhibition of *Ccl5* gene transcription to enhance PDT. The figure was created with Figdraw.com. The data are presented as the means ± SDs for in vitro experiments and means ± SEMs for in vivo experiments

Encouraged by the satisfactory antitumor effect of ARV/Ce6@RDP both in vivo and in vitro, we investigated the mechanism of ARV-825-enhanced PDT. RNA-seq profiling was performed on 4T1 cells and B16F10 cells subjected to different treatments. First, the Ce6@RDP (+) vs RDP and ARV/Ce6@RDP (+) vs Ce6@RDP (+) differentially expressed genes in 4T1 and B16F10 cells are shown in volcano plots (Supplementary Fig. 42a). On the basis of the collection of these differentially expressed genes, we performed intersection analysis on 4T1 cells and B16F10 cells, involving upregulated genes in Ce6@RDP (+) vs RDP, upregulated genes in ARV/Ce6@RDP (+) vs Ce6@RDP (+), downregulated genes in Ce6@RDP (+) vs RDP, and downregulated genes in ARV/Ce6@RDP (+) vs Ce6@RDP (+) (Fig. 7a, b and Supplementary Fig. 42b, c). Seven genes were identified, including the *Ccl5*, *Apol9a*, *Apol9b*, *Ifit1*, *Cox6a2*, *Tcp112*, and *Atf3* genes. The expression of these 7 genes in each group was further characterized via a heatmap (Fig. 7c). Ce6@RDP (+) treatment significantly upregulated *Ccl5*, *Apol9a*, *Apol9b*, *Ifit1*, and *Cox6a2* gene expression, but this upregulation effect was weakened after ARV/Ce6@RDP (+) treatment. In addition, the *Tcp112* and *Atf3* genes were obviously upregulated in the ARV/Ce6@RDP (+) combination group. Furthermore, qPCR analysis of the mRNAs of these 7 genes verified the results of the RNA-sequencing analysis (Supplementary Fig. 43, 44). Among these genes, the *Ccl5* gene, which is related to tumor progression and immune escape, attracted our attention. Moreover, KEGG enrichment indicated that in the 4T1 and B16F10 cells for Ce6@RDP (+) vs RDP, *Ccl5* was included in the 10 genes with the greatest diversity among the 9 most significantly enriched pathways (Supplementary Fig. 45). *Ccl5* has been reported to promote tumor migration, invasion, metastasis and recurrence and to recruit immunosuppressive cells (TAMs and Treg cells) to promote immune escape.^38–40^ Furthermore, UALCAN database analysis revealed that *Ccl5* expression was significantly greater in BRCA and metastatic SKCM (Supplementary Fig. 46a, b). In addition, through the TIMER 2.0 database, *Ccl5* expression was positively correlated with M2 macrophage and Treg immune infiltration in BRCA and SKCM (Supplementary Fig. 46c, d). Therefore, we speculated that *Ccl5* upregulation by PDT limited the therapeutic effect of PDT on breast cancer and melanoma, while the combination of ARV-825 and PDT inhibited the upregulation of *Ccl5*, thereby breaking the tolerance of PDT.

To validate our speculation, western blotting was first performed to detect the protein expression of CCL5 following various treatments. As shown in Supplementary Fig. 47, Ce6@RDP (+) treatment induced the expression of CCL5 in 4T1 and B16F10 cells, whereas this induction was inhibited by ARV/Ce6@RDP (+) treatment, consistent with the increase in *Ccl5* mRNA expression. Because CCL5 is mainly secreted into the extracellular space to play a biological role by binding with the CCR1, CCR3, CCR4 and CCR5 receptors, the secretion of CCL5 was examined, which revealed significantly reduced secretion of CCL5 in the groups supplemented with ARV-825 and obviously increased secretion of CCL5 in the Ce6@RDP (+) group (Fig. 7d). Given that CCL5 downregulation was found in ARV/Ce6@RDP (+)-treated cells compared with Ce6@RDP (+)-treated cells, we next explored whether CCL5 inhibition resulted in the sensitization of tumor cells to PDT. Lentivirus-based knockdown technology was employed to generate *Ccl5* gene-knockdown (sh*Ccl5*-1 and sh*Ccl5*-2) 4T1 cells and B16F10 cells. Quantitative PCR, western blot and ELISA confirmed the successful establishment of *Ccl5*-knockdown cells (Supplementary Fig. 48). The MTT assay demonstrated reduced resistance to Ce6@RDP (+) treatment upon the suppression of *Ccl5*, which was reflected in the decreased viability of *Ccl5*-knockdown cells treated with Ce6@RDP (+) (Fig. 7e). Furthermore, the *Ccl5*-knockdown cells presented a greater apoptotic rate following Ce6@RDP (+) (Fig. 7f), which was accompanied by the inhibition of the P-AKT and BCL2 proteins and the increase in the level of cleaved caspase 3 proteins (Supplementary Fig. 49). Additionally, a similar result was observed in human breast cancer MDA-MB-231 cells and melanoma A375 cells, where Ce6@RDP (+) upregulated CCL5 protein expression and *CCL5* suppression enhanced the effectiveness of Ce6@RDP (+) treatment (Supplementary Fig. 50). Moreover, in the in vivo tumorigenicity assay, Ce6@RDP (+) treatment significantly inhibited the growth of *Ccl5*-knockdown tumors (Fig. 7g-l), indicating a sensitization effect of PDT when *Ccl5* was inhibited. Moreover, no loss of body weight was observed in the Ce6@RDP (+) treatment group (Supplementary Figs. 51 and 52). Further western blot analysis of tumor tissue confirmed that PDT upregulated the expression of CCL5 in vivo and revealed that the upregulation effect of PDT was attenuated in tumors with reduced CCL5 expression (Fig. 7m). Consistent findings were observed via immunohistochemical staining of tumor slices for CCL5 proteins (Supplementary Fig. 53), which might explain why Ce6@RDP (+) combined with *Ccl5* downregulation had a synergistic effect. In addition, the robust efficacy of Ce6@RDP (+) treatment on *Ccl5*-knockdown tumors was further explained by the observation that Ce6@RDP (+) triggered significant proliferation inhibition and apoptosis induction, as evidenced by decreases in P-AKT and BCL2 protein levels and increases in cleaved caspase 3 protein levels (Fig. 7n), which is in agreement with the in vitro findings.

On the basis of the above findings, the hypothesis that CCL5 inhibition sensitizes breast cancer and melanoma to PDT was verified, which also suggested that ARV-825 promoted PDT by downregulating CCL5 expression. To gain insight into the role of CCL5 in PDT, we further examined whether upregulation of the *Ccl5* gene limits the therapeutic effect of PDT. The successful generation of *Ccl5*-overexpressing 4T1 and B16F10 cells was confirmed by the increased expression of *Ccl5* mRNA and secretion of extracellular CCL5 proteins (Supplementary Fig. 54a, b). Subsequent MTT and apoptosis analyses revealed that compared with that in control cells, *Ccl5* overexpression in 4T1 and B16F10 cells significantly reduced the Ce6@RDP (+) effect, as demonstrated by rapid cell proliferation and decreased apoptosis (Supplementary Fig. 54c, d). Thus, the increased secretion of CCL5 induced by PDT could hinder its antitumor effect, implying that ARV-825 reversed PDT tolerance by inhibiting CCL5 expression.

In addition to investigating the effects on cell proliferation and apoptosis, we further investigated the immune regulation mediated by CCL5. As shown in Fig. 7o and Supplementary Fig. 55a, fewer M2 polarized macrophages were observed after incubation with conditioned medium (CM) from *Ccl5*-knockdown tumor cells. In contrast, CM from Ccl5-overexpressing cells induced macrophage M2 polarization (Fig. 7p and Supplementary Fig. 55b), indicating that CCL5 secreted by tumor cells plays a regulatory role in the progression of M2 polarization in macrophages. Subsequent qPCR analysis verified this conclusion, revealing that genes associated with M2 macrophages (*Mrc1*, *Arg1*, *Irf4*, and *Ym1*) were downregulated in the *Ccl*5-knockdown cell medium treatment group, whereas these genes were upregulated in the *Ccl5*-overexpressing cell medium treatment group (Fig. 7q). Moreover, the expression of *the Cd274* gene encoding PDL1 in macrophages also exhibited the same trend, which weakened the antitumor function of T cells. In addition, CCL5 treatment promoted macrophage M2 polarization (Supplementary Fig. 55c, d). Since CCL5 is a chemokine, we next examined the migration of M2 macrophages via CCL5 secreted by breast cancer and melanoma cells. As shown in Fig. 7r and Supplementary Fig. 55e, *Ccl5*-overexpressing tumor cells significantly induced M2 macrophage migration, whereas *Ccl5*-knockdown tumor cells induced much less M2 macrophage migration. These results suggested that CCL5 secreted by tumor cells could induce M2 macrophage polarization and recruit M2 macrophages, indicating that PDT resistance might be partially mediated by the increase in M2 macrophages induced by CCL5. Subsequently, CCL5-mediated M2 macrophages promoted tumor cell proliferation and inhibited CD8^+^ T-cell proliferation, whereas macrophages incubated with *Ccl5*-knockdown cell medium exhibited the opposite effects (Fig. 7s and Supplementary Fig. 55f–h). In addition to examining M2 macrophages, we further investigated the regulation of Tregs by CCL5, and the results indicated that *Ccl5*-knockdown tumor cells slightly reduced the number of Tregs, whereas overexpressing cells upregulated the number of Tregs (Supplementary Fig. 55i and j). Consistently, in vivo assays revealed that tumors with low CCL5 expression presented reduced Treg and M2 macrophage infiltration (Supplementary Figs. 56 and 57), demonstrating that CCL5 could regulate Tregs and M2 macrophages in tumors and that targeting CCL5 was beneficial for improving the tumor immune suppressive microenvironment. Collectively, these observations strongly suggest that ARV-825-mediated CCL5 downregulation can mitigate the immunosuppressive microenvironment mediated by PDT-upregulated CCL5.

The significance of the *Ccl5* gene in PDT prompted us to investigate the potential mechanism by which ARV-825 inhibited *Ccl5* upregulation induced by PDT. Considering that both ROS and BRD4 have been reported to be involved in gene transcription and epigenetic regulation, we next explored the transcriptional regulation of the *Ccl5* gene by ARV-825 and PDT. A dual-luciferase reporter assay revealed that ARV@RDP significantly inhibited the transcriptional activity of the *Ccl5* gene promoter (Fig. 7t). Surprisingly, Ce6@RDP (+) treatment also inhibited the transcriptional activity of the *Ccl5* promoter, indicating that the upregulation of *Ccl5* mRNA induced by PDT might not be primarily mediated by the individual promoter region and might be attributed to other regulatory mechanisms. Considering the potential effects of PDT-induced cellular hypoxia and ATP changes on dual-luciferase reporter gene activity, we further constructed *Ccl5* promoter-GFP and CMV promoter-mCherry plasmids as dual-fluorescence reporter systems to detect the regulatory effects of ARV-825 and PDT on *Ccl5* promoter transcriptional activity. As shown in Supplementary Fig. 58, PDT slightly elevated the ratio of green fluorescence (*Ccl5* promoter-transcribed GFP) to red fluorescence (internal reference mCherry protein), but the addition of ARV-825 inhibited this increase. The results suggested that the increase in *Ccl5* transcription following PDT might be slightly attributed to the increase in its promoter’s transcriptional activity and further confirmed that ARV-825 inhibited *Ccl5* transcription and expression by attenuating the activity of the *Ccl5* promoter. Notably, a significant reduction in transcriptional activity was observed in three different lengths of the *Ccl5* promoter constructed following ARV@RDP treatment, which suggested that ARV-825 could act on multiple sites of the *Ccl5* promoter to exert inhibitory effects. To elucidate the specific site at which ARV-825 targeted the *Ccl5* promoter, a chromatin immunoprecipitation quantitative polymerase chain reaction (ChIP‒qPCR) assay was performed to assess the potential binding of BRD4 proteins to the *Ccl5* promoter. As expected, the results revealed significant enrichment of BRD4 at multiple sites within the 2000 bp sequence of the *Ccl5* promoter (Fig. 7u), indicating that ARV-825 could reduce BRD4 protein binding to the promoter of the *Ccl5* gene, further inhibiting *Ccl5* transcription and expression. The above findings are summarized in Fig. 7v, which shows that ARV-825 reversed tumor growth resistance and immune suppression induced by PDT-mediated CCL5 by inhibiting the transcription of *Ccl5*.

## Discussion

Photodynamic therapy (PDT) has been demonstrated to be a superior approach to combat cancers. Nonetheless, unfavorable treatment outcomes are still observed with PDT alone owing to the complexity of tumors. To optimize drug efficacy, we developed a tumor microenvironment-responsive and targeted Ce6/ARV@RDP micelles that co-delivered the photosensitizer Ce6 and the epigenetic agent (BRD4) PROTAC for the synergistic combination of PDT and epigenetic drug therapy. Integrin α_v_β_3_ has been reported as a tumor biomarker associated with cell migration and invasion and is a basis for the development of targeted nanodelivery systems.^41^ With the modification of the integrin α_v_β_3_ ligand cRGD peptide, the RDP micelles exhibited excellent active targeting ability both in vitro and in vivo, which is consistent with previous reports.^42^ Compared with normal cells, the aberrant metabolism of tumor cells results in acidic microenvironments and high intracellular GSH levels in tumors, which in turn tend to favor the formation of immunosuppressive microenvironments and the attenuation of ROS-based treatment, respectively.^43,44^ The Ce6/ARV@RDP micelles successfully presented acid-triggered charge reversal and GSH-triggered drug release, which might temporarily alleviate acidic and high-GSH environments and further enhance PDT, in addition to realizing tumor accumulation and the release of drugs. This precise release of drugs in tumor cells, enabled by the targeting and tumor microenvironment responsiveness of RDP micelles, is essential for improving efficacy and reducing toxicity, as evidenced by the favorable therapeutic effects in vitro and in vivo and the absence of signs of toxicity post-treatment. Thus, targeted pH/GSH-responsive micelles may hold great promise as a universally applicable delivery platform for combination therapy with PDT and chemotherapeutic drugs.

The antitumor efficacy of PDT is attributed to the direct killing of tumors and the antitumor immune responses elicited by ICD.^45^ However, PDT also plays a role in immunosuppression, which leads to suboptimal therapeutic outcomes.^46,47^ We observed that ARV-825 significantly enhanced PDT, as evidenced by the inhibition of tumor cell proliferation and angiogenesis, the induction of apoptosis both in vitro and in vivo, and the promotion of antitumor immune responses and alleviation of the immunosuppressive TME. This sensitizing effect may be attributed to the degradation of BRD4 proteins involved in the regulation of diverse tumor biological processes by ARV-825. ATP, a distinct marker of ICD, acts as a “find me” signal to DCs and macrophages, thereby promoting the recruitment of myeloid cells.^48^ The cell surface-exposed CRT, another sign of ICD occurrence, serves as the “eat me” signal to APCs by binding to low-density lipoprotein receptor-related protein 1 (LRP1 or CD91), further facilitating the phagocytosis of DCs and macrophages.^49^ ATP release and CRT exposure stimulate the presentation of tumor-associated antigens from APCs to cytotoxic T cells, leading to the initiation of an immune response. Interestingly, ARV/Ce6@RDP performed better than Ce6@RDP in terms of ATP release, CRT exposure, DC maturation, and the activation of cytotoxic T cells, which may be explained by previous observations that epigenetic modifiers have the ability to induce ICD.^50,51^ However, the specific mechanism underlying the enhanced ICD resulting from BRD4 inhibition remains uncertain and is worthy of investigation in future work. The interaction between the ligand molecule PDL1 and its receptor PD1 suppresses the cytotoxic activity of T cells against cancer cells, thereby facilitating immune evasion.^52^ Our data demonstrated that ARV/Ce6@RDP effectively reduced the upregulation of PDL1 induced by PDT and exhibited outstanding immune response performance in vivo, suggesting that ARV-825 could enhance PDT by downregulating PDL1 expression. Notably, we found that ARV-825 downregulated the protein expression of CD47. An important role of CD47 is to act as a “do not eat me” signaling molecule that inhibits the phagocytosis of macrophages and DCs by binding to glycoprotein signal regulatory protein α (SIRPα) on the surface of macrophages and DCs, thus hindering antigen presentation and safeguarding cells from attack by the immune system.^53^ Further testing demonstrated the superior phagocytosis of macrophages or DCs on tumor cells treated with ARV/Ce6@RDP, echoing previous observations of enhanced antigen presentation and immune activity. These findings indicated that ARV-825 enhanced PDT-mediated antitumor immunity. Additionally, previous studies have reported that BRD4-targeting agents exert regulatory effects on both macrophages and T cells, including the inhibition of macrophage recruitment and polarization,^18,54^ as well as the modulation of T-cell activity.^15,55^ When combined with other immunotherapies, these drugs enhance the antitumor immune response.^56^ These findings suggest that the immune responses induced by PDT in our study may also influence the immunomodulatory effects mediated by ARV-825.

More importantly, we found that PDT elicited *Ccl5* gene transcription and protein expression as well as secretion, whereas ARV-825 had an antagonistic effect on *Ccl5*. Notably, CCL5, a multifunctional chemokine, has dual effects on tumors, depending on the specific tumor type and microenvironment.^57–60^ Our data suggested that CCL5 was not conducive to PDT against breast cancer and melanoma, which was substantiated by observations that knockdown of *Ccl5* potentiated the PDT effect, including cell proliferation inhibition and apoptosis induction in vitro, as well as tumor growth suppression in vivo. Conversely, *Ccl5* upregulation attenuated the effectiveness of PDT. In addition, the overexpression of *Ccl5* has been demonstrated to promote the M2 polarization of macrophages and the recruitment of M2 macrophages and increase the population of Tregs, whereas the knockdown of *Ccl5* elicits opposite effects, which is in line with the literature reports.^61–64^ This finding reveals a novel mechanism of tumor resistance to PDT and indicates that the pronounced sensitization of PDT to ARV-825 is partly due to the suppression of CCL5. The revelation of new resistance mechanisms is crucial for advancing the clinical application and development of PDT in cancer treatment. However, the mechanisms underlying PDT resistance are likely multifactorial and complex, as several alternative resistance pathways, such as DNA damage repair pathways,^65^ protective autophagy,^66^ cellular antioxidant defenses,^67^ and immunosuppressive tumor microenvironments,^9^ have been reported in previous studies. The upregulation of CCL5 may interact with these processes, contributing to the complexity of PDT resistance. It is also possible that regulatory relationships exist among CCL5 and these pathways, which warrants further investigation in future studies. In addition, our findings also offer a new perspective on tumor drug resistance during PDT; that is, the upregulation of CCL5 in tumor cells post-treatment drives multiple biological processes, such as cell proliferation, apoptosis, and the immune response, to trigger resistance to treatment. Therefore, further exploration of the mechanism by which PDT and ARV-825 induce alterations in CCL5 is essential to optimize clinical PDT and develop effective combined therapy strategies. ARV-825 significantly inhibited the transcriptional activity of the *Ccl5* promoter. This phenomenon was subsequently explained by the interference of ARV-825 in the binding of the BRD4 protein to the *Ccl5* promoter, further suppressing the transcription of the *Ccl5* gene. This finding also revealed how resistance caused by PDT-induced CCL5 upregulation can be reversed through gene regulatory mechanisms involving epigenetic modifiers. However, PDT slightly promoted the transcriptional activity of the *Ccl5* promoter, which suggested that the upregulation of *Ccl5* induced by PDT may not be primarily mediated by directly affecting promoter activity to increase *Ccl5* gene transcription. Instead, this may indicate the presence of a more complex regulatory mechanism that warrants thorough investigation in future work. Furthermore, our study suggests the potential for alternative combined antitumor strategies, which involve the combination of CCL5 inhibitors and PDT, thereby overcoming the tolerance of PDT.

**Fig. 8:**
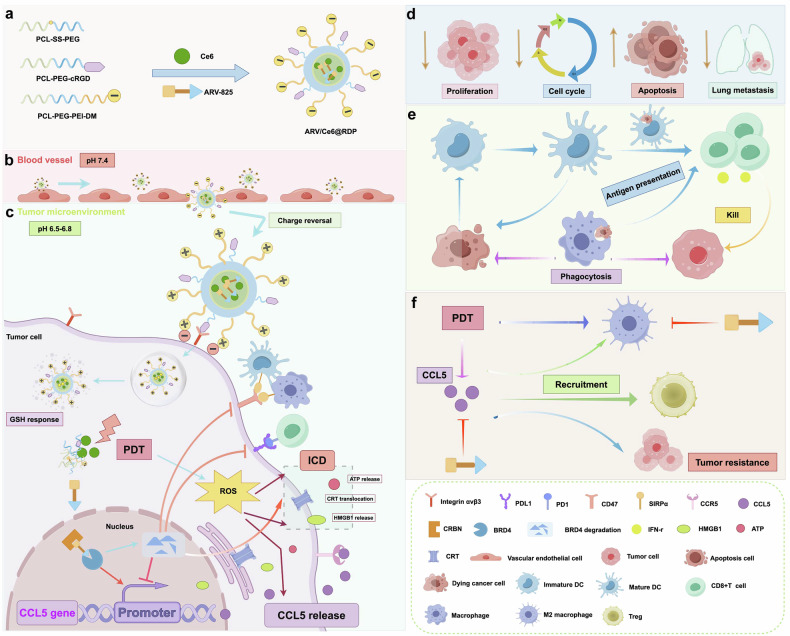
Schematic illustration of the design and antitumor implementation of ARV/Ce6@RDP micelles. **a** Self-assembly of ARV/Ce6@RDP micelles. **b** Transport of the micelle in blood vessels. **c** Tumor microenvironment response of ARV/Ce6@RDP micelles and multiple antitumour effects triggered by ARV-825 and PDT. **d** Inhibition of proliferation, the cell cycle, and lung metastasis, as well as the induction of apoptosis by ARV/Ce6@RDP micelles. **e** Increased antitumor immune response to PDT by ARV-825. **f** Reversal of PDT resistance by ARV-825. The figure was created with Figdraw.com

Taken together, as depicted in Fig. 8, the ARV/Ce6@RDP micelles are more likely to be located in the tumor cells because of the selectivity of the cRGD peptides modified on the carrier toward integrin α_v_β_3_ expressed on the tumor cells. Moreover, the micelle transitioned from the blood (pH 7.4) to the tumor microenvironment (pH 6.5–6.8), reversing its surface charge to enhance cellular uptake. The micelle subsequently released ARV-825 and Ce6 in response to intracellular GSH. The Ce6 released from micelles, as photosensitizers, generates ROS under laser irradiation, further causing cytotoxic effects and ICD. In addition, the released ARV-825 linked BRD4 proteins and the E3 ligase Cereblon (CRBN), which in turn triggered the degradation of BRD4 through the ubiquitin proteasome system. ARV-825 subsequently exhibited a synergistic antitumor effect on PDT, as demonstrated by its ability to inhibit proliferation, arrest the cell cycle, induce apoptosis, and suppress lung metastasis. Furthermore, the combined ICD effect and downregulation of PDL1 and CD47 mediated by ARV-825 cooperatively activated the antitumor immune response, as manifested in increased DC maturation, enhanced presentation of tumor-associated antigens by DCs and macrophages, and activation of CD8^+^ T cells. Mechanistically, ARV-825 reduced the binding of the BRD4 protein to the *Ccl5* promoter and further inhibited *Ccl5* gene transcription, thereby inhibiting the upregulation of *Ccl5* induced by PDT, improving PDT resistance, and remodeling the tumor microenvironment by decreasing the number of immunosuppressed cells (M2 macrophages and Tregs). Overall, our results revealed the potential of PDT combined with BRD4 PROTACs in cancer therapy.

In summary, our work developed a novel programmed delivery platform targeting integrin α_v_β_3_, with the photosensitizers Ce6 and ARV-825 encapsulated in a hydrophobic layer. The delivery platform enabled efficient intracellular drug accumulation, thereby further enhancing therapeutic efficacy. Moreover, we fully demonstrated that combining ARV-825 with PDT significantly enhanced antitumor efficacy via multiple mechanisms, which can be attributed to increasing PDT activity and overcoming PDT resistance, suggesting potential applications for clinical cancer therapy.

## Materials and methods

### Ethics statement

The research complied with all relevant ethical regulations of Sichuan University. All animal protocols were performed in accordance with the Animal Experimental Ethics Committee of the State Key Laboratory of Biotherapy, Sichuan University.

### Reagents

Methoxy poly(ethylene glycol) carboxyl acid (MPEG-COOH, MW 2000), hydroxyl- and carboxyl-terminated PEG (HO-PEG-COOH, MW 2000), and hydroxyl- and maleimide-terminated PEG (HO-PEG-MAL, MW 2000) were purchased from Shanxi Xinyan Bomei Biotechnology Co., Ltd. ε-Caprolactone was obtained from Tokyo Chemical Industry. 3-(4,5-Dimethyl-2-thiazolyl)-2,5-diphenyl-2H-tetrazolium bromide (MTT), a cell cycle detection kit, and an Annexin V-FITC/PI apoptosis detection kit were obtained from Melunbio. The 1,3-diphenylisobenzofuran (DPBF) and reactive oxygen species (ROS) detection kits (DCFH-DA) were purchased from Shanghai Titan Scientific Co., Ltd. Hoechst 33342, 2-(4-amidinophenyl)-6-indolecarbamidine dihydrochloride (DAPI), and LysoTracker Green were acquired from Beyotime.

### Cell lines and animals

Mouse 4T1, B16F10, and human MDA-MB-231, A375 cell lines were obtained from the American Type Culture Collection. 4T1-Luc cells were generated via stable transfection with luciferase. B16F10 cells were cultured in RPMI 1640 medium supplemented with 10% fetal bovine serum (FBS) and 1% penicillin‒streptomycin. Other cell lines were cultured in DMEM supplemented with 10% FBS and 1% penicillin‒streptomycin. BALB/c mice and C57BL/6 mice were purchased from Beijing Huafukang Biotechnology Co., Ltd., and 6–8-week-old female mice were used for all experiments unless otherwise noted.

### Fabrication and characterization of the nanoformulations

Ce6 and ARV-825 were homogenized separately or together in acetone containing dissolved MPEG-SS-PCL, PCL-PEG-PEI-DM, and cRGD-PEG-PCL, and the resulting mixture was slowly added to deionized water under the action of ultrasound. The residual acetone in the solution was removed through evaporation and stirred overnight, finally obtaining drug-loaded micelles (ARV/Ce6@RDP, Ce6@RDP, ARV@RDP) via 0.45 µm membrane filtration. Blank micelles containing RDP and untargeted Ce6@DP (without cRGD-PEG-PCL) were prepared via the same method. The size distributions and zeta potentials of RDP, ARV@RDP, Ce6@RDP, and ARV/Ce6@RDP were detected via a laser particle size analyzer (Brookhaven Instruments, USA). The morphology of ARV/Ce6@RDP was analyzed via transmission electron microscopy (TEM, FEI, USA). In addition, the UV–vis spectra of the nanoformulations were measured via a UV–vis spectrometer (U-T3C, China).

### pH/GSH responsiveness of ARV/Ce6@RDP

The pH-responsive surface charge reversal of ARV/Ce6@RDP was evaluated via zeta potential measurements after incubation in PBS at pH values of 7.4 and 6.8. Furthermore, the morphology of the micelles at pH 6.8 was visualized via TEM. ARV/Ce6@RDP was dispersed in glutathione (GSH) solution, and the particle size was measured at different time points (0, 1, 2, 4, 6, 8, 10, 12, and 24 h). In addition, samples incubated with GSH for 24 h were observed via TEM.

### In vitro cellular uptake of micelles

4T1 cells and B16F10 cells were separately seeded into a 12-well plate at a density of 1 × 10^5^/well and cultured overnight. Then, Ce6@RDP micelles were added to selected wells and cultured for different durations (2, 4, 8, 12, and 24 h) at 37 °C, after which the cells were washed with PBS and analyzed via flow cytometry (FCM, ACEA NovoCyte, USA). In addition, after coincubation and DAPI staining, the cellular uptake of the micelles was further observed via a high-content imaging system (HCI, PE/Opera Phenix Plus, USA).

For analysis of the ability of RDP micelles to target cells, integrin α_v_β_3_ expression in tumor cells (4T1, B16F10, MDA-MB-231, and A375 cells) was first proven. Briefly, cDNA from tumor cells was subjected to qPCR, followed by agarose electrophoresis of the resulting qPCR products to confirm the expression of the *Itgav* and *Itgb3* genes. To observe the expression of the ITGAV protein on the cell membrane more intuitively, tumor cells were subjected to immunofluorescence staining with a TIGAV antibody, followed by staining with DAPI and examination with a confocal laser scanning microscope (CLSM, LSM 880 with Airyscan, Zeiss, Germany). Afterward, tumor cells were left untreated or treated separately with Ce6, Ce6@DP, or Ce6@RDP for 4 h, after which the cells were collected, and the intracellular Ce6 fluorescence was analyzed via FCM. The targeted uptake of RDP micelles was further demonstrated through an RGD peptide blockade assay. Briefly, cells were pretreated with 0.5 mg/mL c(RGDyC) for 30 min before the addition of Ce6@RDP micelles. After incubation for 4 h, the cells were washed twice with PBS, and flow analysis was performed. Additionally, the tumor cells were cultured with Ce6, Ce6@DP, Ce6@RDP, or Ce6@RDP+cRGD (c(RGDyC) pretreatment) for 4 h. Then, the cells were stained with Hoechst 3342, followed by observation with HCl.

The charge-reversal properties of the RDP micelles were further verified through their cellular uptake. Specifically, cells were seeded in a 96-well plate (4000 cells/well) the previous day, after which the culture supernatants were replaced with pH 7.4 or pH 6.8 media before the addition of Ce6@RDP. Following a 4 h incubation at 37 °C, Hoechst 3342 was used to stain the cell nucleus before imaging with HCl. For quantitative analysis of cellular uptake, cells that had undergone Ce6@RDP (pH 7.4 or pH 6.8) incubation were harvested to detect Ce6 fluorescence via FCM. Furthermore, tumor cells were treated with ARV/Ce6@RDP for 4 h to observe the colocalization of lysosomes and micelles. LysoTracker and Hoechst 3342 were used to label the nucleus and lysosomes, respectively, followed by imaging observations through HCI.

### Live animal imaging

BALB/c mice and C57BL/6 mice were subcutaneously injected with 1×10^6^ 4T1 cells and B16F10 cells, respectively. Upon reaching a tumor volume of approximately 200 mm^3^, the mice were randomly assigned to three groups and received separate administrations of free Ce6, untargeted Ce6@DP, or targeted Ce6@RDP (5 mg/kg Ce6 component) via the tail vein. The fluorescence signals were captured at 1, 3, 6, 8, 12, and 24 h via a live animal in vivo imaging system (IVIS, PerkinElmer, USA). After the last imaging, the organs (heart, liver, spleen, lung, and kidney) and tumor tissues of the mice were harvested and further imaged via an in vivo imaging system to observe the distribution of Ce6.

### Cellular ROS generation

Tumor cells were seeded in 6-well plates and incubated overnight. After being treated with RDP, ARV@RDP, Ce6@RDP, or ARV/Ce6@RDP at a Ce6 concentration of 4 μg/mL for 8 h, the cells were incubated with DCFH-DA (10 μM) solution without FBS for 20 min, followed by laser irradiation or not. The cells were subsequently stained with Hoechst 3342 for capture via a fluorescence inverted microscope (Olympus, Japan) for qualitative analysis of cellular ROS generation, and the ROS quantitative data were acquired via FCM.

### Western blotting

The total cell lysates were prepared with RIPA buffer supplemented with protease inhibitor cocktail, followed by quantification of proteins with a BCA kit (Beyotime). The extracted proteins were denatured by boiling and fractionated by SDS‒PAGE. Proteins were subsequently transferred onto polyvinylidene difluoride membranes, followed by blocking, incubation with primary antibodies, and incubation with secondary antibodies. The antibodies used for immunoblotting are summarized in Supplementary Table 1, following the manufacturer’s instructions. Images were acquired with an e-blot Touch Imager (China).

### Quantitative PCR (RT‒qPCR)

The total RNA was extracted with a UNlQ-10 column total RNA purification kit (Sangon Biotech, China) in accordance with the manufacturer’s instructions, and then the RNA was retrotranscribed with HiScript III SuperMix for qPCR (+gDNA wiper) (Vazyme, China). Quantitative real-time PCR (RT‒PCR) was conducted using a LightCycler® 480 real-time fluorescent quantitative PCR instrument (Roche, Switzerland). *Gapdh* or *β-actin* were employed as reference genes for normalizing gene expression, which was calculated via the delta‒delta Ct method (2^−^^ΔΔCt^). The sequences of primers used are summarized in Supplementary Table 2.

### MTT assay

4T1, B16F10, MDA-MB-231, or A375 cells were inoculated in 96-well plates overnight and then treated with different nanomedicines for 8 h. Following treatment, the cells were washed with PBS, and fresh culture medium was added before laser irradiation. After an additional 24 h incubation period, the MTT experiment was performed as previously described.^18^ The absorbance of each well was measured with a microplate reader (Thermo Fisher Scientific, USA) at 570 nm.

### Apoptosis assay

The cells were seeded in 12-well plates for 12 h and then incubated with RDP, ARV@RDP, Ce6@RDP, or ARV/Ce6@RDP for 8 h, followed by laser irradiation and incubation for an additional 24 h. The cells were harvested and stained with the Annexin V-FITC/PI Cell Apoptosis Kit following the manufacturer’s instructions. Finally, flow cytometry was used to analyze the degree of apoptosis in the samples.

### Cell cycle analysis

The cells were subjected to apoptosis assays as outlined above and subsequently collected for propidium iodide (PI) staining according to the instructions of the Cell Cycle Detection Kit. Briefly, following a PBS wash and overnight fixation at −20 °C in 70% ice-cold ethanol, the harvested cells were stained with PI for 30 min at 37 °C. The cells were then analyzed for alterations in DNA content via FCM.

### Detection of ATP

Following a 24 h treatment period with the aforementioned apoptosis assay, 4T1 cell and B16F10 cell supernatants were collected for ATP detection with an ATP luminescent cell viability assay kit (Yeasen) according to the manufacturer’s instructions. Specifically, 100 μL of each supernatant was dispensed into black 96-well plates (Thermo Fisher Scientific) before the addition of 100 μL of ATP detection reagent. The mixture was then agitated for 2 min and allowed to stand for 10 min at room temperature, followed by luminescence detection via a multifunctional microplate reader (BioTek, Synergy H1, USA).

### Isolation and maturation of BMDCs

Bone marrow-derived dendritic cells (BMDCs) were isolated from the tibia and femur of 6-week-old BALB/C or C57BL/6J mice. Specifically, after the bone was sterilized with 75% alcohol, the bone marrow cells were rinsed with a syringe. Single cells were obtained by filtration with a 70 μm cell strainer, followed by erythrocyte depletion with red blood cell lysis buffer. The cells were subsequently washed with serum-free medium and then cultured in complete culture medium supplemented with 20 ng/mL GM-CSF and 10 ng/mL IL-4. After a three-day incubation period, fresh medium containing GM-CSF and IL-4 was added to the cells. The BMDCs were then harvested for experiments after seven days. For the in vitro maturation study of BMDCs, tumor cells were inoculated into the lower chamber of a transwell system and treated with various micelles, including RDP, ARV@RDP, Ce6@RDP (+), and ARV/Ce6@RDP (+), as outlined above. Following laser irradiation, BMDCs were incubated in the upper chamber for 24 h. Afterward, stimulated BMDCs were collected, with a portion used for subsequent experiments, and a portion was stained with anti-CD11c, anti-CD80, anti-CD86, and anti-MHCII antibodies before flow cytometry analysis.

### Analysis of T cells in vitro

Tumor cells were cultured in a 12-well plate overnight, followed by various treatments. After laser irradiation, lymphocytes from the mouse spleen were added to the treated tumor cells for co-incubating for 48 h. Subsequently, the cells were collected and stained with anti-CD3 and anti-CD8a antibodies to analyze the numbers of CD8^+^ T cells via flow cytometry. For the activation of T cells, stimulated BMDCs and lymphocytes from mouse spleens were co-cultured in a 12-well plate at a 1:5 ratio for 24 h. Afterward, the cells were collected and then stained with anti-CD3, anti-CD8a, anti-CD4, and anti-IFN-γ antibodies, followed by flow cytometry analysis.

### Isolation and polarization of BMDMs

Bone marrow cells were isolated via the same method as described for BMDC isolation. Bone marrow-derived macrophages (BMDMs) were then obtained by culturing the cells in medium containing 20 ng/mL M-CSF for six days. The BMDMs were subsequently subjected to various treatments, followed by flow cytometry to analyze the M2 polarization of the BMDMs via anti-CD11b, anti-F4/80, and anti-CD206 staining. Total mRNA was subsequently extracted to detect M2 macrophage–associated genes via RT‒qPCR.

### Phagocytosis of tumor cells by BMDCs and BMDMs

The tumor cells were stained with CFSE before being stimulated with RDP, ARV@RDP, Ce6@RDP, or ARV/Ce6@RDP. Following laser irradiation, BMDCs or BMDMs were incubated for 12 h at a ratio of 5:1. The cells were subsequently collected and stained with anti-CD11c for BMDCs or anti-CD11b and anti-F4/80 for BMDMs, followed by flow cytometry analysis. For further visualization of cellular phagocytosis, CFSE-labeled tumor cells upon treatment with RDP, ARV@RDP, Ce6@RDP (+), or ARV/Ce6@RDP (+), were coincubated with DiD-labeled BMDMs or BMDCs in confocal dishes for 12 h, followed by imaging via a confocal laser scanning microscope (CLSM, LSM 900, Zeiss, Germany).

### Subcutaneous xenograft tumor mouse model

4T1 cells or B16F10 cells (1 × 10^6^) were subcutaneously injected into the right flank of BALB/c or C57BL/6 mice. The tumor volume was monitored and was approximately 50–100 mm^3^, after which the mice were randomly assigned to eight groups (PBS, RDP, ARV@RDP, Ce6@RDP, Ce6@RDP (+), ARV/Ce6@RDP, ARV/Ce6@DP (+), and ARV/Ce6@RDP (+)). The treatments were performed every two days for a total of four intravenous administrations (Ce6 dose: 5 mg/kg, ARV-825 dose: 10 mg/kg). Following 3 h of administration, the mice in the Ce6@RDP (+), ARV/Ce6@DP (+), and ARV/Ce6@RDP (+) treatment groups were exposed to a 660 nm laser at 100 mW/cm^2^ for 10 min. The tumor volume and body weight of the mice were monitored every two days throughout the treatment period. At the end of treatment, the mice were sacrificed, and then, the tumors were extracted, photographed, weighed, and fixed in 4% paraformaldehyde for subsequent immunohistochemistry (IHC) and immunofluorescence (IF) analysis. Furthermore, the main organs (heart, liver, spleen, lung, and kidney) and serum of the mice were harvested for hematoxylin‒eosin (H&E) staining and blood biochemistry analysis, respectively.

### Postoperative recurrence and metastasis mouse model

4T1 cells labeled with luciferase (1 × 10^6^) were subcutaneously implanted into the right flank of BALB/c mice. When the tumor volume reached approximately 300 mm^3^, the tumors were surgically resected, and the mice were subsequently randomized into four groups. On the fourth day after surgery, the mice received various treatments (PBS, ARV@RDP, Ce6@RDP (+), and ARV/Ce6@RDP (+)), which were repeated five times at intervals of one day. After surgery and treatment, the tumors in each group were visualized through an in vivo bioluminescence imaging system following intraperitoneal injection of D-luciferin. The tumor recurrence rates and body weights of the mice in each group were monitored and recorded every two days. When the maximum recurrence volume of the tumor reached 1500 mm^3^, the mice were sacrificed, and the tumor tissues were extracted and weighed. In addition, the lungs of the mice were harvested to count the number of pulmonary metastatic nodules in each group and fixed in paraformaldehyde for H&E staining analysis.

### Flow cytometry analysis of immune cells in vivo

4T1 or B16F10 subcutaneous tumor-bearing mice were established via the aforementioned method. The mice were assigned to different groups and injected intravenously with PBS, ARV@RDP, Ce6@RDP, or ARV/Ce6@RDP, followed by laser irradiation at 3 h intervals for the Ce6@RDP and ARV/Ce6@RDP groups. After four administrations, the tumor-draining lymph nodes and tumor tissues were collected to prepare single-cell suspensions. The cells that formed the lymph nodes were stained with anti-CD11c, anti-CD80, anti-CD86, and anti-MHCII antibodies. The cells from the tumor tissues were subjected to various fluorescence-conjugated antibodies to label T cells (L/D^−^CD45^+^CD3^+^CD4^+^CD8a^+^), Treg cells (L/D^−^CD45^+^CD3^+^CD4^+^Foxp3^+^), DCs (L/D^−^CD45^+^CD11c^+^CD80^+^CD86^+^; L/D^−^CD45^+^CD11c^+^MHCII^+^), M2 macrophages (L/D^−^CD45^+^CD11b^+^F4/80^+^CD206^+^), NK cells (L/D^−^CD45^+^CD3^−^CD49b^+^CD107a^+^) and MDSCs (L/D^−^CD45^+^CD11b^+^Gr1^+^). Dead cells were excluded via the Zombie NIR^TM^ Fixable Viability Kit (#423105, BioLegend), while nonspecific antibody binding was prevented by blocking FcR with TruStain FcXTM PLUS (anti-mouse CD16/32, #156604, BioLegend). All the experimental procedures were conducted in accordance with the manufacturer’s instructions, and the results were analyzed via a flow cytometer. All the flow cytometry antibodies used are listed in Supplementary Table 3.

### Immunohistochemistry (IHC) and immunofluorescence (IF) of tumor tissues

The formalin-fixed and paraffin-embedded (FFPE) tumor tissues were subjected to a series of steps, including sectioning, dewaxing, hydration, antigen retrieval, blocking of endogenous peroxidase, and sealing with serum. For IHC, the slides were subsequently separately stained with primary antibodies against Ki67 (#GB111141, Servicebio), CD31 (#GB113151, Servicebio), PDL1 (#ab213480, Abcam), CCL5 (SC-365826, Santa Cruz), CD206 (#GB113497, Servicebio), and Foxp3 (#GB112325, Servicebio), followed by incubation with an HRP-conjugated secondary antibody. Color development was achieved via the use of a peroxidase substrate DAB kit, with counterstaining performed via hematoxylin. For the IF assay, tumor slides were subjected to TUNEL staining according to the TUNEL Apoptosis Detection Kit (BD Bioscience). In addition, calreticulin (CRT) exposure in tumors was examined via immunofluorescence staining with an anti-CRT antibody (#GB112134, Servicebio). For CD8^+^ T cells and M2-like macrophages, the tumor slides were subjected to immunofluorescence staining with anti-CD8 antibody (#GB114196, Servicebio) and anti-CD206 antibody (#GB113497, Servicebio), respectively. For Treg cells, slides were stained with an anti-CD4 antibody (#GB15064, Servicebio) and an anti-Foxp3 antibody (#GB112325, Servicebio). DAPI was used to stain the nuclei in all the immunofluorescence-stained sections. Images of the IHC or IF tumor sections were captured with an upright microscope (Olympus, Japan) and a pathological scanner (3DHISTECH, Hungary) or via quantitative pathology imaging and analysis (Vectra Polaris, USA).

### RNA sequencing (RNA-seq)

4T1 cells or B16F10 cells were subjected to the indicated treatment, followed by lysis in TRIzol reagent (Ambion). The samples were subsequently collected for RNA extraction, RNA testing, library construction, sequencing on the Illumina platform, and data analysis implemented by Maiwei Metabolic Biotechnology Co., Ltd. (Wuhan, China).

### Plasmid construction and establishment of stable cell lines

The shRNA against *Ccl5* was inserted into the pLKO.1 vector, and the plasmid encoding *Ccl5* was cloned and inserted into the pCDH-CMV-MCS-EF1-Puro vector. The primer sequences utilized in this study are summarized in Supplementary Table 4. Stable cell lines with *Ccl5* gene knockdown and overexpression were established through lentiviral infection. To achieve this, the recombinant plasmid was co-transfected with packaging PSPAX2 and PMD2. G vectors into HEK293T cells. The culture medium containing virus particles was subsequently harvested 48 h post-transfection and used to infect 4T1, B16F10, MDA-MB-231, and A375 cells, followed by selection with puromycin.

### ELISA

After treatment, the cell culture supernatants were collected to detect the secretion of CCL5 via a CCL5 ELISA kit (#88-56009, Invitrogen) according to the manufacturer’s protocol. Briefly, the ELISA plate was coated with the captured antibodies overnight before blocking. The samples were added, followed by a 2 h incubation with the detection antibody and washing. The diluted streptavidin HRP conjugate was subsequently added and incubated for 30 min at room temperature, after which the substrate solution was added for color development prior to the addition of the HCl stop solution. The optical density was assessed via a microplate reader at a wavelength of 450 nm.

### Dual-luciferase reporter assay

The promoter fragments of *Ccl5* (-1953 - +57 bp, -979 - +8 bp, and -150 - +65 bp) were inserted into the pGL3-basic vector to generate *Ccl5* firefly luciferase reporters. The primer sequences are listed in Supplementary Table 5. B16F10 cells were cultured in 12-well plates and then co-transfected with *Ccl5* luciferase reporters and Renilla luciferase reporters. After 4 h of transfection, the cells were subjected to various treatments (RDP, ARV@RDP, Ce6@RDP (+), and ARV/Ce6@RDP (+)) for 24 h. Luciferase activities were determined via a Dual-Luciferase Reporter Assay Kit (MA0518-L, Meilunbio) following the product instructions.

### ChIP‒qPCR

Chromatin immunoprecipitation (ChIP) assays were performed with a Simplechip Enzymatic Chromatin IP Kit (#9003, CST) following the manufacturer’s instructions. Briefly, tumor cells were crosslinked with 1% formaldehyde, followed by chromatin digestion and sonication. Chromatin samples were precipitated with rabbit anti-BRD4 antibody (#83375, CST) or an equivalent amount of normal rabbit IgG isotype control (#2729, CST) prior to the addition of protein G magnetic beads. Following chromatin elution and reverse cross-linking with protease K, the DNA was purified and subsequently quantified via a qPCR assay with the primers listed in Supplementary Table 6.

### Statistical analysis

All the data are presented as the means ± SDs for in vitro experiments and as the means ± SEMs for in vivo experiments. Data analyses were performed via GraphPad Prism software 8.0. Statistical analysis was performed with two-tailed Student’s *t*-tests for comparisons between two groups, two-way ANOVA for comparisons among multiple groups, and the log-rank test for survival studies. The results were considered statistically significant if *P* < 0.05, and those with *P* > 0.05 had no statistical significance, represented by ns.

## Supplementary information

Revised supplementary materials

## Data Availability

The RNA-seq data used in this study have been deposited in the NCBI Sequence Read Archive (SRA) with the BioProject number PRJNA1346556. The data in Supplementary Figs. 46a, b were downloaded directly from the online UALCAN database (https://ualcan.path.uab.edu/). Supplementary Fig. 46c, d data were downloaded directly from the online TIMER 2.0 database (http://timer.comp-genomics.org/). The remaining data are available within the article and Supplementary Information.
