# Supplementary material for: Employing epigenetic protein degradation techniques to block CCL5-mediated photodynamic therapy via a programmed delivery platform
Source: Signal Transduct Target Ther. 2026 Jan 30;11:30. doi: 10.1038/s41392-025-02542-y (PMC12855822; doi:10.1038/s41392-025-02542-y)
Supplement: Supplementary file 1 — Revised supplementary materials [file 41392_2025_2542_MOESM1_ESM.docx]

Supplementary Materials for

Employing epigenetic protein degradation techniques to block CCL5-regulating photodynamic therapy mediated by a programmed delivery platform

Tingting Yang^1^, Yuzhu Hu^1^, Anjie Guo^1^, Xifeng Zhang^1^, Wanyu Wang^1^, Linbin Yi^1^, Rui Zhang^1^, Xinyu Gou^1^, Zhiyong Qian^1^, Bilan Wang^2,3,4,5*^, Yongzhong Cheng^1,*^, Xiang Gao^1,*^

* Corresponding author: Bilan Wang, Yongzhong Cheng and Xiang Gao

1. mail: [bilan@scu.edu.cn](mailto:bilan@scu.edu.cn), chengyz@scu.edu.cn and [xianggao@scu.edu.cn](mailto:xianggao@scu.edu.cn)

**This PDF file includes:**

Materials and Methods

Figures S1 to S58

Tables S1 to S7

**Materials and Methods**

**Molecular dynamics simulation**

Molecular dynamics simulation of the interaction between MPEG-SS-PCL, Ce6 and ARV-825 was performed by NAMD software. Briefly, MPEG-SS-PCL polymer molecules and drug small molecules were first randomly placed within a 10 angstrom (0.1nm) distance range from each other, and then the phase transition process of the two molecules approaching and being embedded in each other was calculated and simulated using molecular dynamics simulation in NAMD software. The molecular dynamics time was at least 20 ns.

**Synthesis of MPEG-SS-PCL**

MPEG-COOH (1 g), 1-Ethyl-3-(3-dimethylaminopropyl) carbodiimide hydrochloride (EDC, 143.8 mg) and N-Hydroxysuccinimide (NHS, 86.3 mg) were homogenized in anhydrous N, N-Dimethylformamide (DMF, 20 mL) by magnetic stirring under an ice bath for 30 min, followed by adding bis(2-hydroxyethyl) disulfide (154.2 mg) to react for 24 h at room temperature. Subsequently, the resulting products were dialyzed for 3 days and then obtained MPEG-SS-OH through vacuum freeze drying, with further characterization conducted using proton nuclear magnetic resonance (^1^H NMR) spectroscopy. Next, ε-Caprolactone (1 g) and the obtained MPEG-SS-OH (1 g) were melted by heating, followed by the addition of Tin (II) 2-ethylhexanoate (Sn(Oct)_2_) for an 12 h reaction at 140℃ under a nitrogen atmosphere. The product was dissolved in dichloromethane and then precipitated with cold petroleum ether, which was repeated three times for initial purification. Further purification was performed through dialysis and vacuum to obtain MPEG-SS-PCL, which was characterized by ^1^H NMR analysis.

**Synthesis of PCL-PEG-PEI-DM**

HO-PEG-COOH (1 g), ε-Caprolactone (1 g) and the catalyst Sn(Oct)_2_ were melted and the reaction were kept at 140℃ under nitrogen protection. After 12 h, the product PCL-PEG-COOH was purified using MPEG-SS-PCL purification method mentioned above. The structure of PCL-PEG-COOH was characterized by ^1^H NMR. Subsequently, the carboxyl group of obtained PCL-PEG-COOH (1 g) was activated with EDC (2 eq) and NHS (2 eq) in a dimethyl sulfoxide (DMSO) system for 1 h, followed by addition of PEI (1.5 eq) for reaction under the room temperature. After 24 h, the product was dialyzed against deionized water and further freeze-dried to yield PCL-PEG-PEI, with characterization via ^1^H NMR. The obtained PCL-PEG-PEI (1 g) and excess 2,3-dimethylmaleic anhydride (DMMA) were dissolved in DMSO, and then stirred for 24 h, followed by dialysis and lyophilization for obtaining final product PCL-PEG-PEI-DM. The prepared PCL-PEG-PEI-DM was characterized by ^1^H NMR.

**Synthesis of cRGD-PEG-PCL**

PCL-PEG-MAL was synthesized by the above ring opening polymerization method and was characterized by ^1^H NMR. The identified PCL-PEG-MAL and excess c(RGDyC) were homogenized in DMSO, and then stirred for 24 h. The product was dialyzed against deionized water to remove unreacted c(RGDyC), further undergoing lyophilization to obtain cRGD-PEG-PCL. Subsequently, the structure of cRGD-PEG-PCL was characterized by ^1^H NMR.

**Evaluations of ARV/Ce6@RDP stability**

The ARV/Ce6@RDP was respectively dispersed in PBS solution and 10% FBS culture medium, followed by monitoring changes of particle size at time points of 0, 2, 4, 8 and 24 h. Besides, the long-time stability of ARV/Ce6@RDP also was evaluated via detecting the change in particle size over 50 days.

**Determinant of critical micelle concentration (CMC)**

The critical micelle concentration of the ARV/Ce6@RDP micelle was ascertained by the light scattering method. Briefly, the light scattering intensity and particle size of ARV/Ce6@RDP micelles with different concentrations (1-1000 μg/mL) was measured by dynamic light scattering (DLS) analyzer.

**Hemolysis assessment**

Red blood cells were collected from mouse blood, and then incubated with 1 mL of ddH_2_O, PBS, 10% FBS culture medium and ARV/Ce6@RDP at 37℃ for 4 h respectively. After centrifugation (1000 rpm, 15 min), each group of samples was photographed to observe hemolysis. Subsequently, the supernatant was collected and analyzed for UV absorption spectra and absorbance at 540 nm.

**Single line oxygen measurement**

DPBF reagent as singlet oxygen (^1^O_2_) probe was employed for detection of ^1^O_2_ generated by ARV/Ce6@RDP or Ce6@RDP following laser exposure, as evidenced by a decrease in absorbance at approximately 410 nm resulting from the reaction between DPBF and ^1^O_2._ A freshly prepared DPBF solution in DMF was combined with either ARV/Ce6@RDP or Ce6@RDP micelle. Free Ce6 with the equivalent concentration, blank micelles RDP and the only BPDF served as control. The samples were subjected to laser irradiation at a wavelength of 660 nm and power density of 100 mW/cm^2^ for 0.5, 1, 2, 3, 4, 6, and 8 minutes. The characteristic decrease in absorbance at approximately 410 nm was quantified using UV-vis spectroscopy. The efficiency of ^1^O_2_ generation was calculated using the remaining DPBF (%) equation: A_t_/A_0_ × 100%, where A_t_ was the absorbance of DPBF after irradiation for t min and A_0_ was the absorbance of before irradiation.

**Immunofluorescence staining for cells**

Round coverslips were placed in 24-well plates before cells implantation. After specific treatment or no treatment, the cells were blocked with 5% normal goat serum for 60 min at room temperature following fixation with 4% formaldehyde for 15 min. For the intracellular protein, the cell membrane was permeabilizated using 0.1% Triton X-100 for 5 min before blocking. The cells were incubated with primary antibodies against anti-TIGAV (#CY6887, Abways, 1:200), anti-CRT (#12238S, CST, 1:400) and anti-HMGB1 (#3935S, CST, 1:50) overnight at 4℃, followed by incubation with Alexa Fluor 488-labeled secondary antibody (#ab150077, Abcam, 1:1000) for 1 h at room temperature. The cells were stained with DAPI and captured using a laser confocal microscope (LSM 880 with Airyscan, Zeiss, Germany).

**PDL1 inhibition by ARV/Ce6@RDP**

4T1 and B16F10 cells were seeded in 12-well plates overnight. After being treated by RDP, ARV@RDP, Ce6@RDP (+) and ARV/Ce6@RDP (+) for 24 h, cells were collected to stain with anti-PDL1 for 30 min at 4℃ and analyze by FCM. Additionally, treated cells were harvested to detect PDL1 protein expression by western blot.

**Survival analyses**

To evaluate the effect of ARV/Ce6@RDP on survival rates, a 4T1 or B16F10 subcutaneous tumor mouse model was established as described above. Subsequently, the mice were assigned to four groups (PBS, ARV@RDP, Ce6@RDP (+) and ARV/Ce6@RDP (+)) and subjected to the same treatment as before. The survival of the mice post-treatment was also recorded, and mice were considered deceased either upon death or when the tumor volume exceeded 2000 mm³.

**In vivo safe evaluation**

The formalin-fixed and paraffin-embedded (FFPE) organ tissues, including the heart, liver, spleen, lung, and kidney, underwent histological staining with H&E to assess potential histological changes following different therapeutic interventions. Additionally, serum samples were collected post-treatment to analyze blood biochemical indexes (ALB, ALP, ALT, AST, TP2, AMYL, CREJ2, UREAL, UA2, GLUC3, CHO2I, HDLC4, LDLC3, TRIGL, LDHI2, CKMB2) using a blood biochemical instrument (Roche, Switzerland).

**PDT for *Ccl5* knockdown or overexpression tumor cells**

For in vitro assay, tumor cells with *Ccl5* gene knockdown (sh*Ccl5*-1 and sh*Ccl5*-2) and control cells (shNC) were seeded into 12-well plates overnight, and then treated with or without PDT, followed by MTT assay for cells cytotoxicity, flow cytometry for cells apoptosis, and western blot for related proteins change. Similarly, *Ccl5*-overexpression cells (oe*Ccl5*) and control cells (oeNC) were performed to analyze cell viability and apoptosis after treatment with or without Ce6@RDP (+).

For in vivo assay, a subcutaneous xenograft *Ccl5*-knockdown tumor-bearing mouse model was established as previously described. Briefly, 4T1 or B16F10 cells (1×10^6^) from each group (shNC, sh*Ccl5*-1 and sh*Ccl5*-2) were subcutaneously injected into mice, followed by performing 4 times of PDT and recording tumor volume and body weight of mice in each group. Tumors were harvested at the end of the experiment for measuring tumor weight, IHC staining and extracting proteins to western blot analysis.

**Migration of M2 macrophages**

BMDMs were stimulated with IL4 (20 ng/mL) for 24 h for polarizing into M2 type macrophages, and then co-cultured with *Ccl5* knockdown or overexpression tumor cells in a transwell to induce migration. After 48 h, the migrating macrophages were stained with crystal violet and photographed (Olympus, Japan).

**Proliferation analysis by CFSE staining**

BMDMs exposed to different conditioned medium from tumor cells were co-cultured with CFSE-labeled tumor cells or lymphocytes from spleen in an indirect co-culture system or direct co-culture system respectively. Subsequently, cells were collected, and tumor cells were directly analyzed for proliferation via flow cytometry, while lymphocytes stained with anti-CD3 and anti-CD8a and subjected to flow cytometric analysis.

**Tregs detection in vitro**

Tumor cells with either *Ccl5* knockdown or overexpression were implanted into the lower chamber of a transwell system and incubated overnight. Subsequently, lymphocytes isolated from the spleen were introduced into the upper chamber for co-incubation over a period of 48 hours. Cells were collected and stained with anti-CD3, anti-CD4, anti-CD25 and anti-Foxp3, followed by flow cytometry analysis.

**Detection of *Ccl5* promoter activity by a dual fluorescent plasmid system**

The *Ccl5* promoter-GFP plasmid was first constructed by replacing the CMV promoter on PVAX-GFP with the promoter fragments of *Ccl5* (-1953 - +57 bp), and then the mcherry gene was inserted into the PVAX vector to generate CMV promter-mcherry plasmid as a control plasmid. The primer sequences were listed in Supplementary Table 7. Subsequently, the *Ccl5* promoter-GFP plasmid and CMV promter-mcherry plasmid were co-transfected into tumor cells. After a 4 h transfection, cells were exposed to various treatments, followed by photographed and flow cytometry analysis.


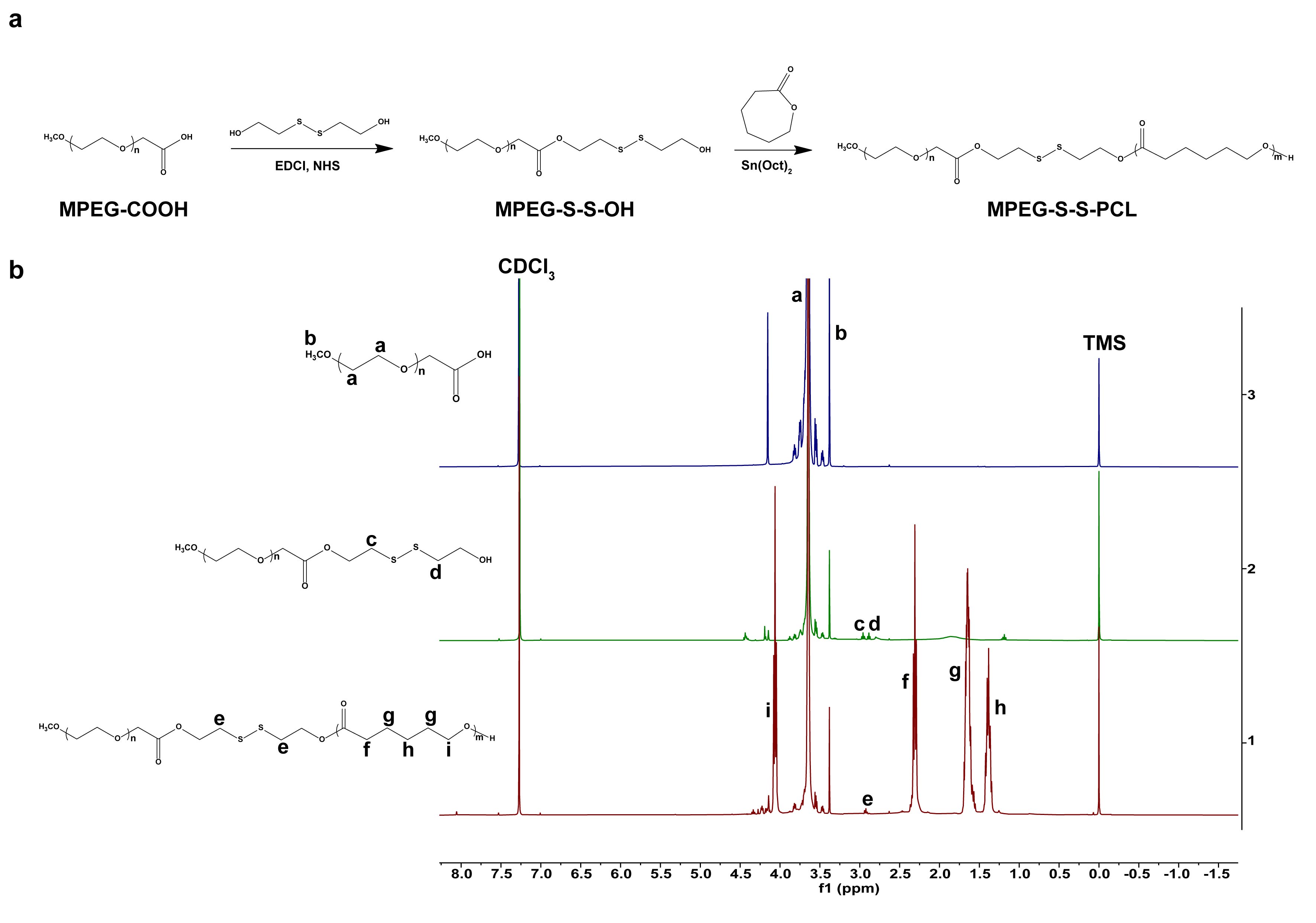


**Figure S1.**

**The synthesis and characterization of MPEG-SS-PCL**. (**a**) The synthetic route of MPEG-SS-PCL. (**b**) ^1^H NMR spectrum of MPEG-SS-PCL.


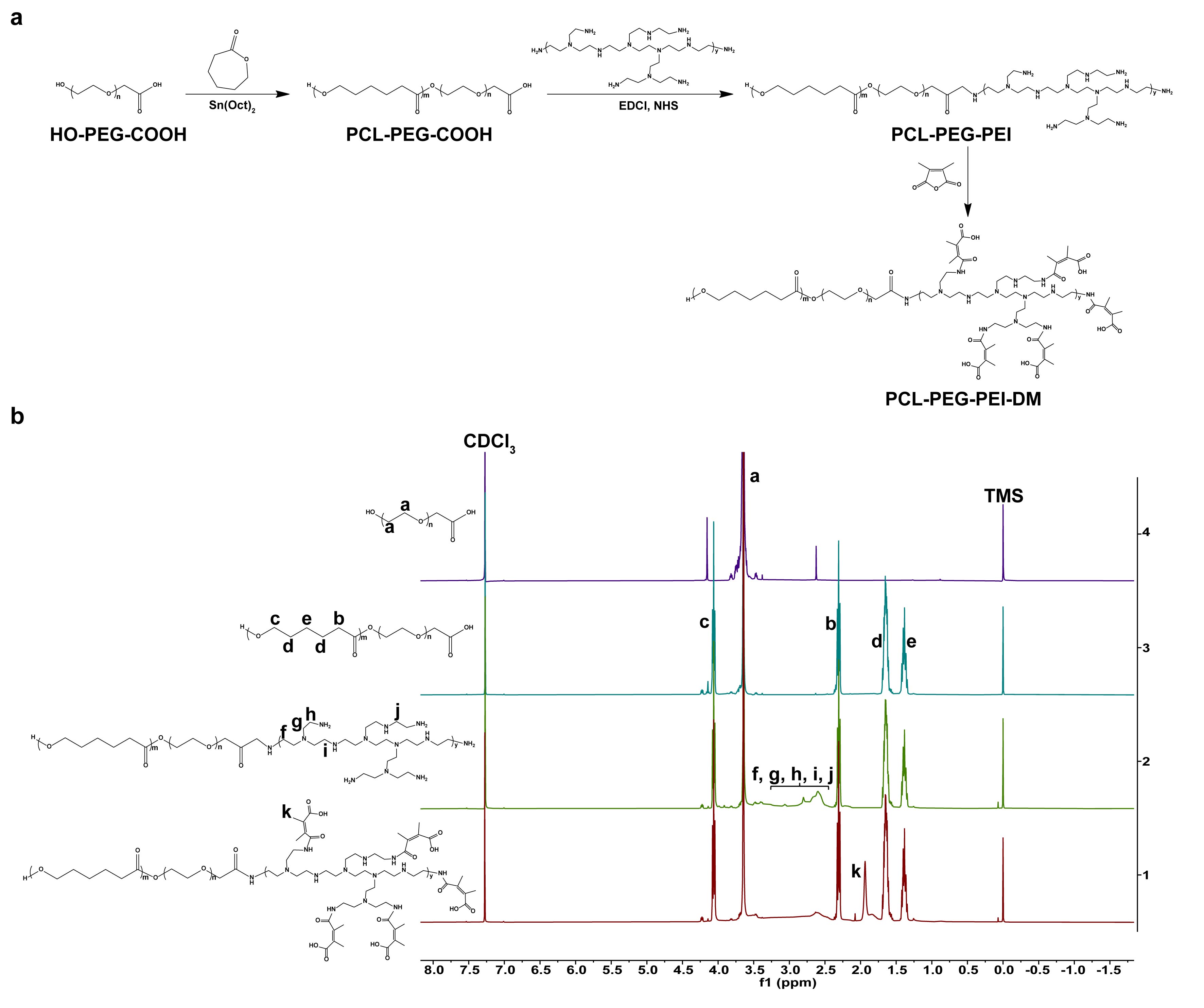


**Figure S2.**

**The synthesis and characterization of PCL-PEG-PEI-DM**. (**a**) The synthetic route of PCL-PEG-PEI-DM. (**b**) ^1^H NMR spectrum of PCL-PEG-PEI-DM.


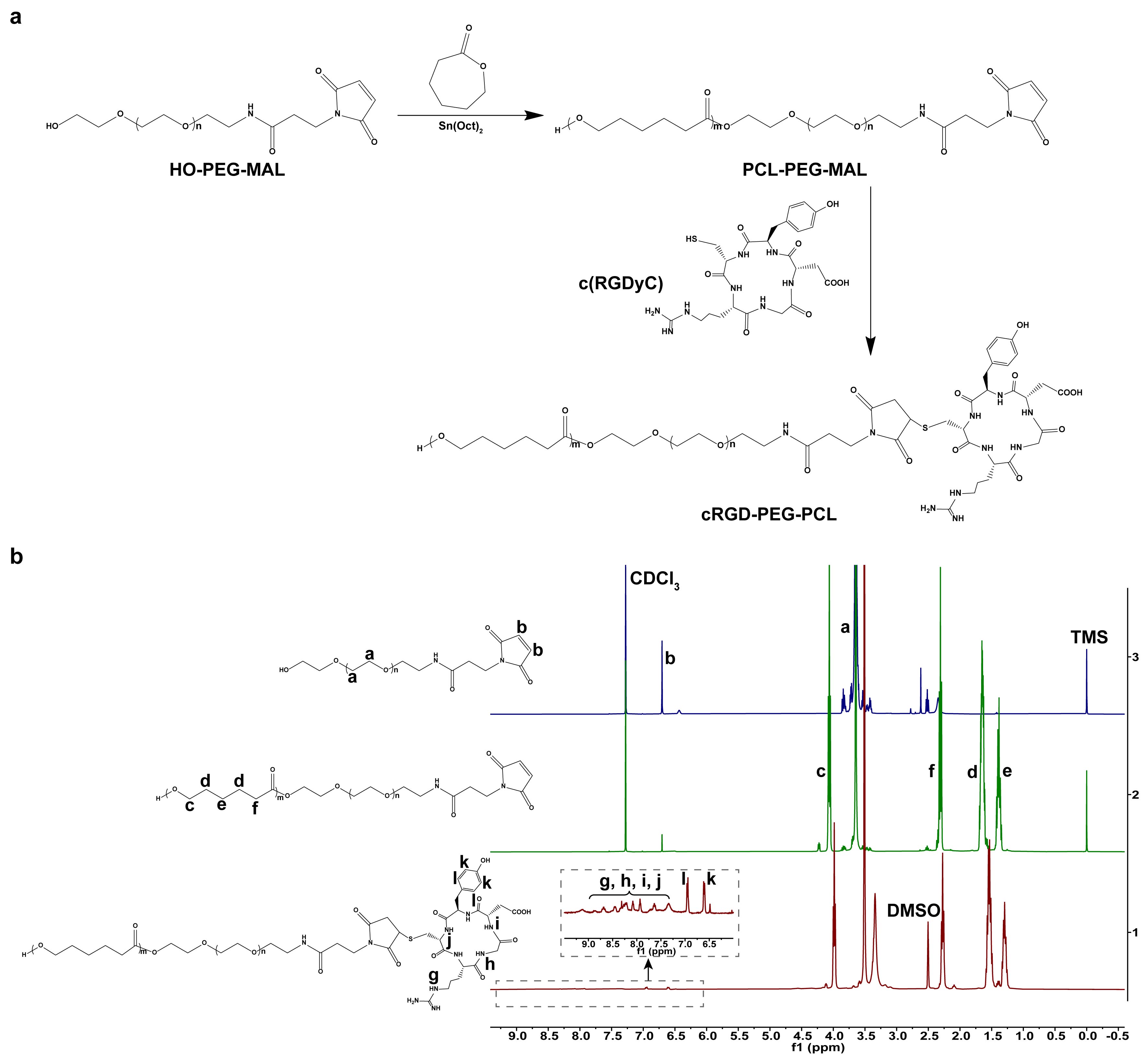


**Figure S3.**

**The synthesis and characterization of cRGD-PEG-PCL**. (**a**) The synthetic route of cRGD-PEG-PCL. (**b**) ^1^H NMR spectrum of cRGD-PEG-PCL.


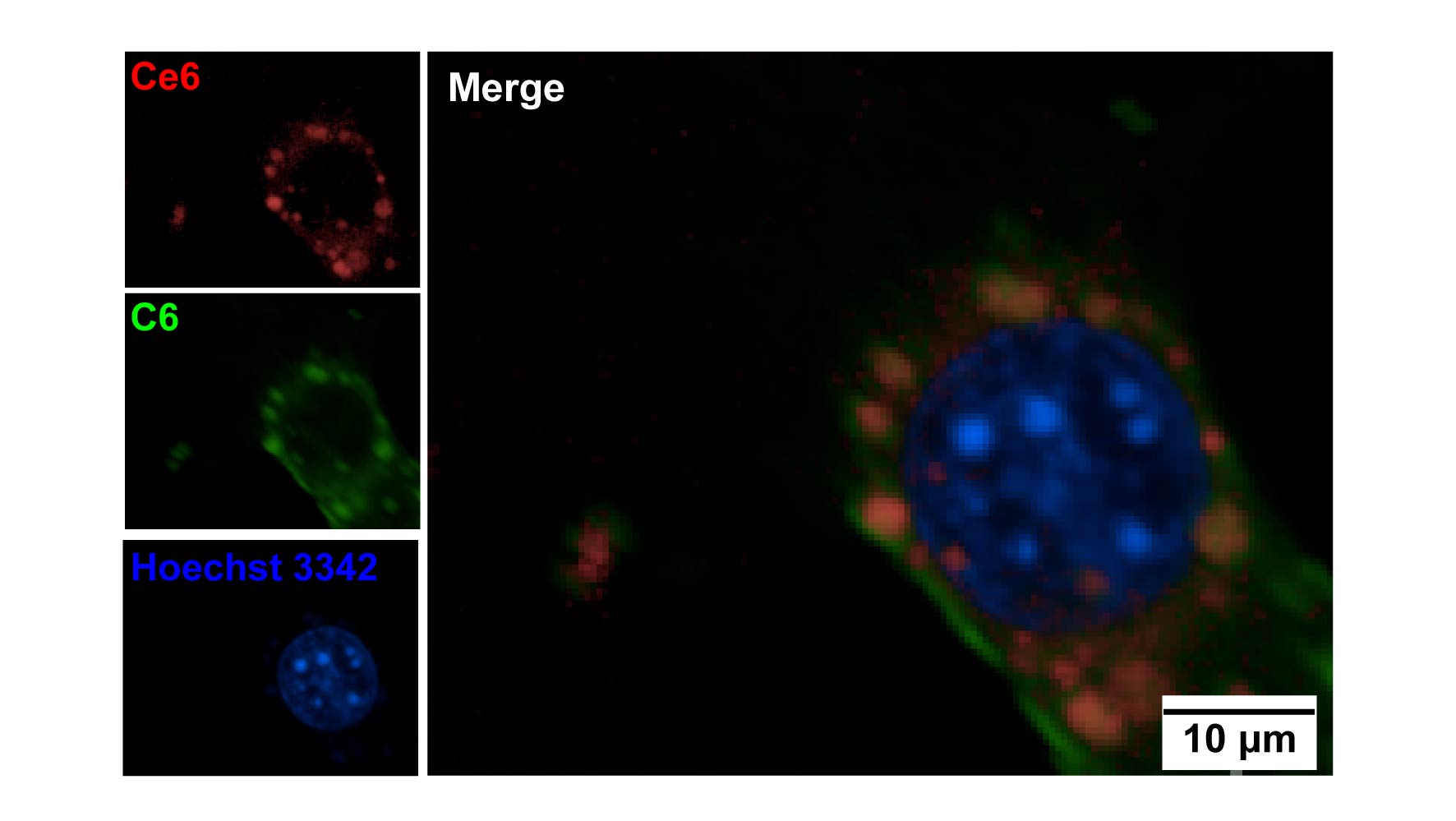


**Figure S4.**

**The cellular uptake of the co-assembled Ce6 and C6 micelles by 4T1 cells observed under HCI (blue: nucleus; green: C6, red: Ce6)**. Scale bar: 10 µm.


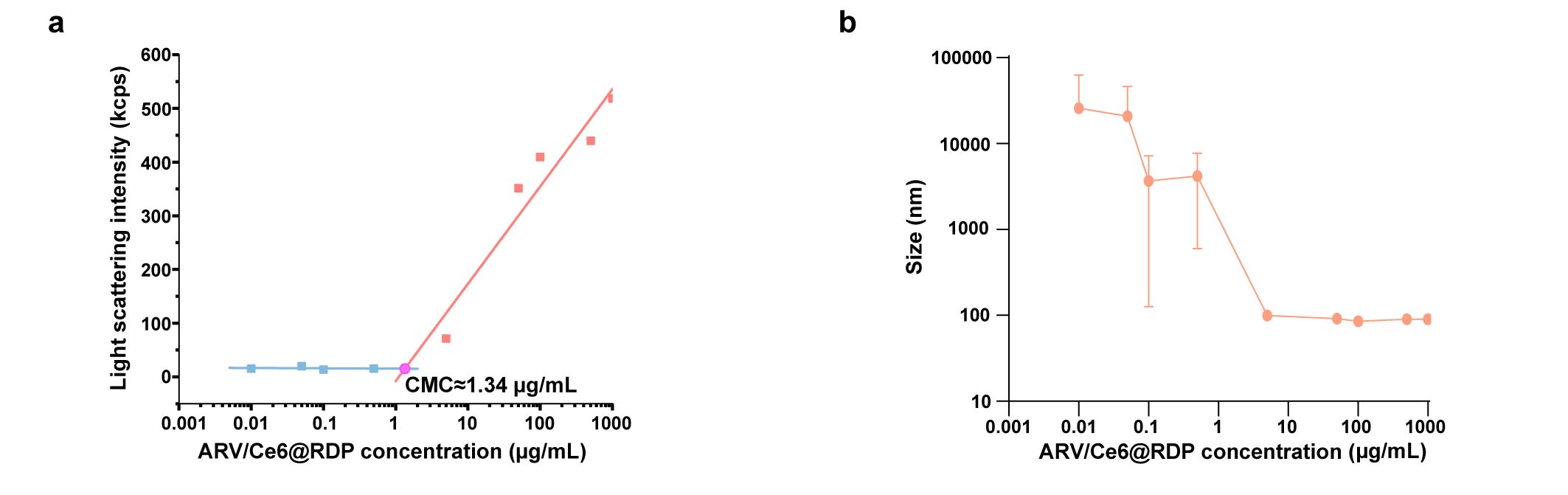


**Figure S5.**

**Critical micelle concentrations (CMCs) of ARV/Ce6@RDP micelles**. (**a**) Changes in light scattering intensity of the ARV/Ce6@RDP micelle at different dilutions. (**b**) Changes in particle size of the ARV/Ce6@RDP micelle at different dilutions (n = 3 per group, data are shown as mean ± SDs).


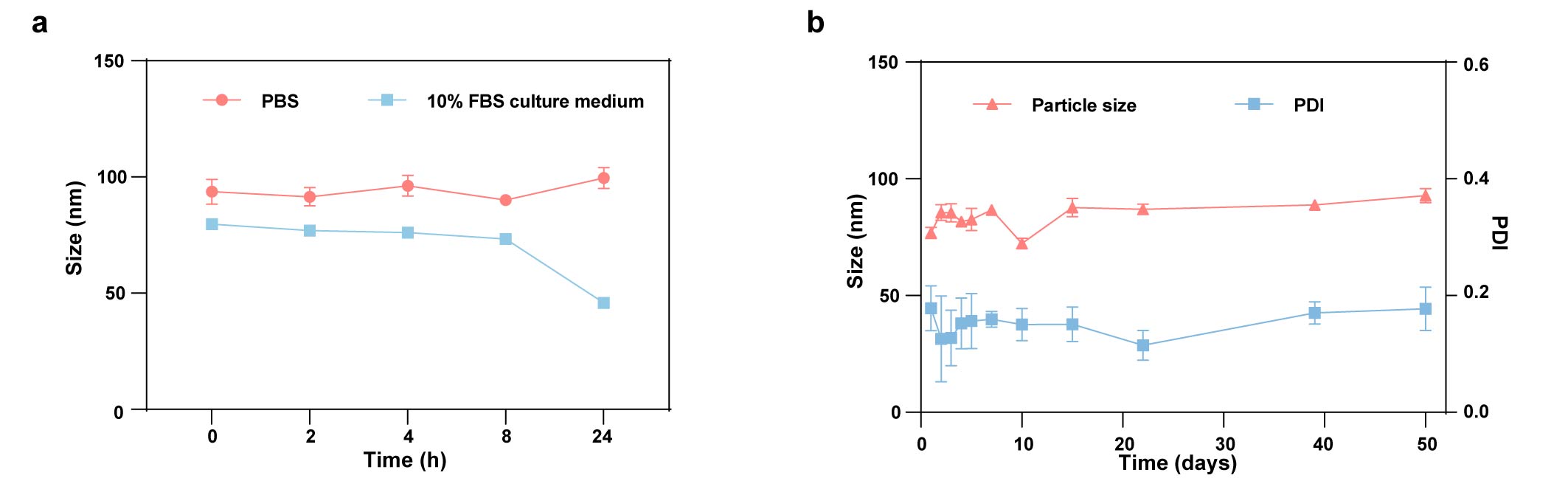


**Figure S6.**

**The stability of ARV/Ce6@RDP micelles**. (**a**) The stability of ARV/Ce6@RDP micelles in PBS and 10% FBS culture medium. Store at 37°C for 24 h (n = 3 per group). (**b**) Long-term stability of ARV/Ce6@RDP micelles (n = 3 per group). All data in this figure are shown as mean ± SDs.


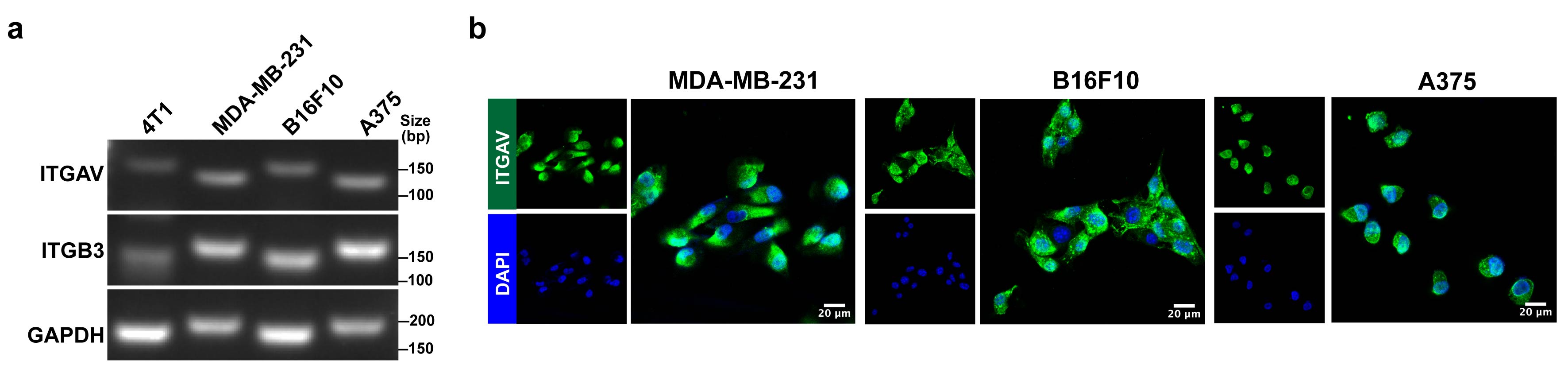


**Figure S7.**

**Expression of integrin α_v_β_3_ in tumor cells**. (**a**) Agarose gel electrophoresis image of RT-qPCR products of *Itgav* and *Itgb3* genes in 4T1, MDA-MB-231, B16F10 and A375 cells. (**b**) Confocal immunofluorescence images of ITGAV proteins in MDA-MB-231, B16F10 and A375 cells (blue: nucleus; green: ITGAV proteins). Scale bar: 20 µm.


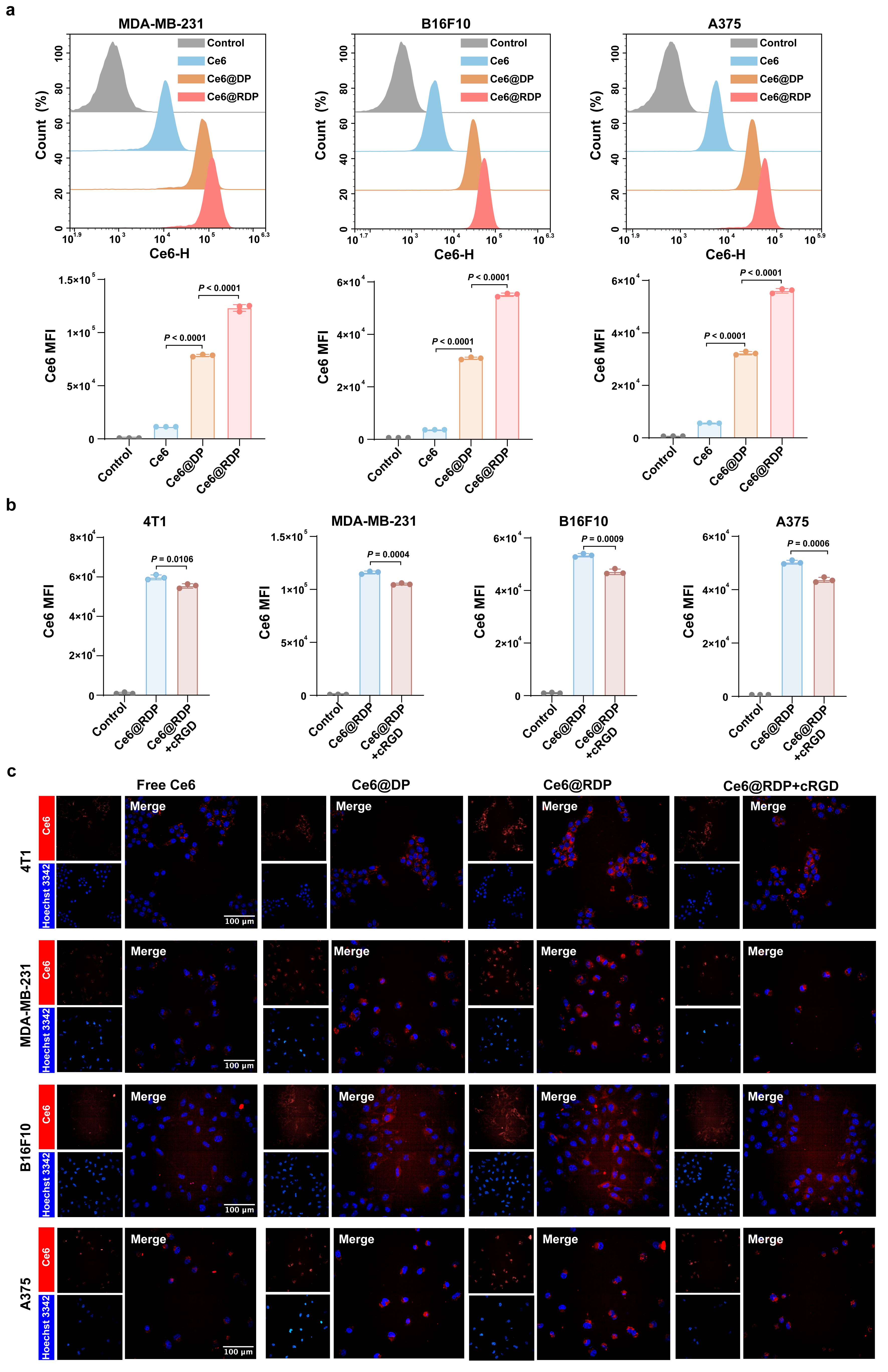


**Figure S8.**

**Cellular uptake of micelles**. (**a**) Cellular uptakes in MDA-MB-231, B16F10 and A375 cells of free Ce6, Ce6@DP and Ce6@RDP measured by flow cytometry (n = 3 per group, two-tailed unpaired Student’s *t* test). (**b**) Flow analysis for cellular uptake in 4T1, MDA-MB-231, B16F10 and A375 cells of Ce6@RDP after blocking integrin α_v_β_3_ with cRGD peptide (n = 3 per group, two-tailed unpaired Student’s *t* test). (**c**) HCI images of 4T1, MDA-MB-231, B16F10 and A375 cells incubated with free Ce6, Ce6@DP, Ce6@RDP and Ce6@RDP+cRGD for 4 h (blue: nucleus; red: Ce6 fluorescence). Scale bar: 100 µm. All data in this figure are shown as mean ± SDs.


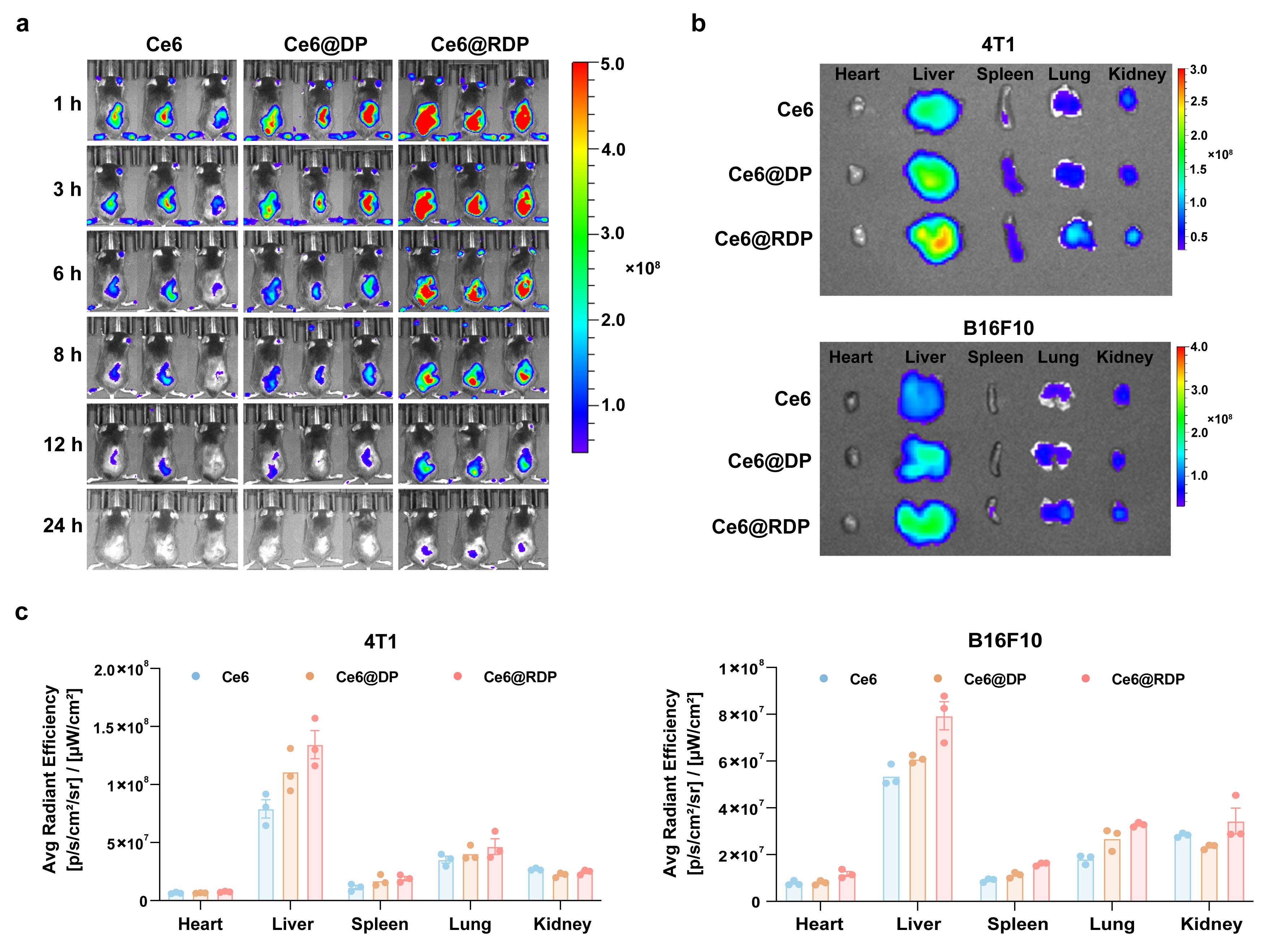


**Figure S9.**

**Tumor accumulation of micelles**. (**a**) In vivo fluorescence images of the B16F10 tumor-bearing mice at 1, 3, 6, 8, 12 and 24 h post-injection of free Ce6, Ce6@DP and Ce6@RDP. (**b**) Ex vivo fluorescence imaging of main organs (Heart, Liver, Spleen, Lung and Kidney) of 4T1 and B16F10 tumor-bearing mice after 24 h injection. (**c**) Ex vivo fluorescence quantitative analysis of main organs of 4T1 and B16F10 tumor-bearing mice after 24 h injection (n = 3 per group, data are shown as mean ± SEMs).


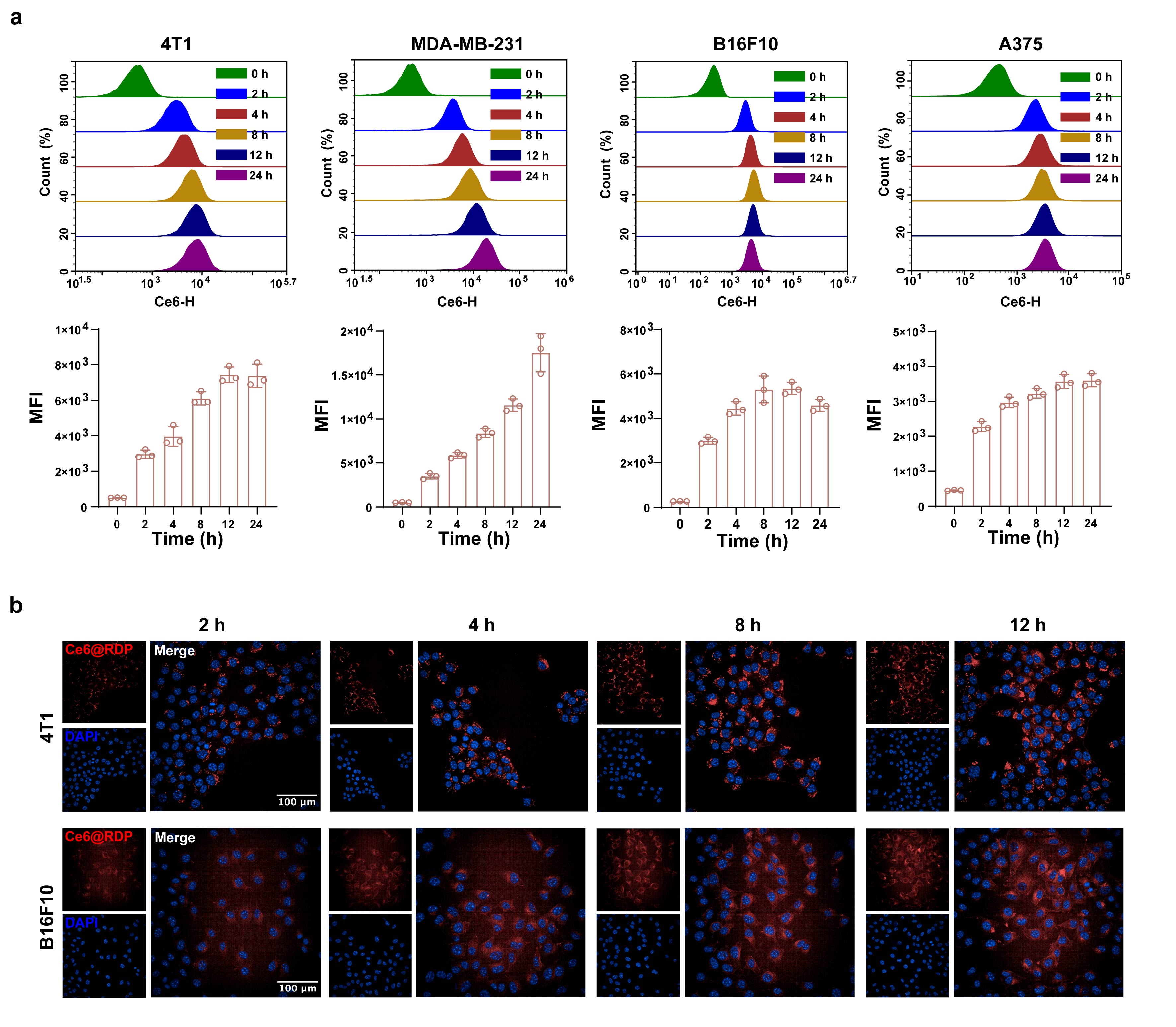


**Figure S10.**

**Cellular uptakes of Ce6@RDP in tumor cells at different time points**. (**a**) Flow cytometric analysis of the cellular uptakes of Ce6@RDP in 4T1, MDA-MB-231, B16F10 and A375 cells at different time points (n = 3 per group). (**b**) HCI images of 4T1 and B16F10 cells after incubating with Ce6@RDP for different time points (blue: nucleus; red: Ce6@RDP). Scale bar: 100 µm.

**
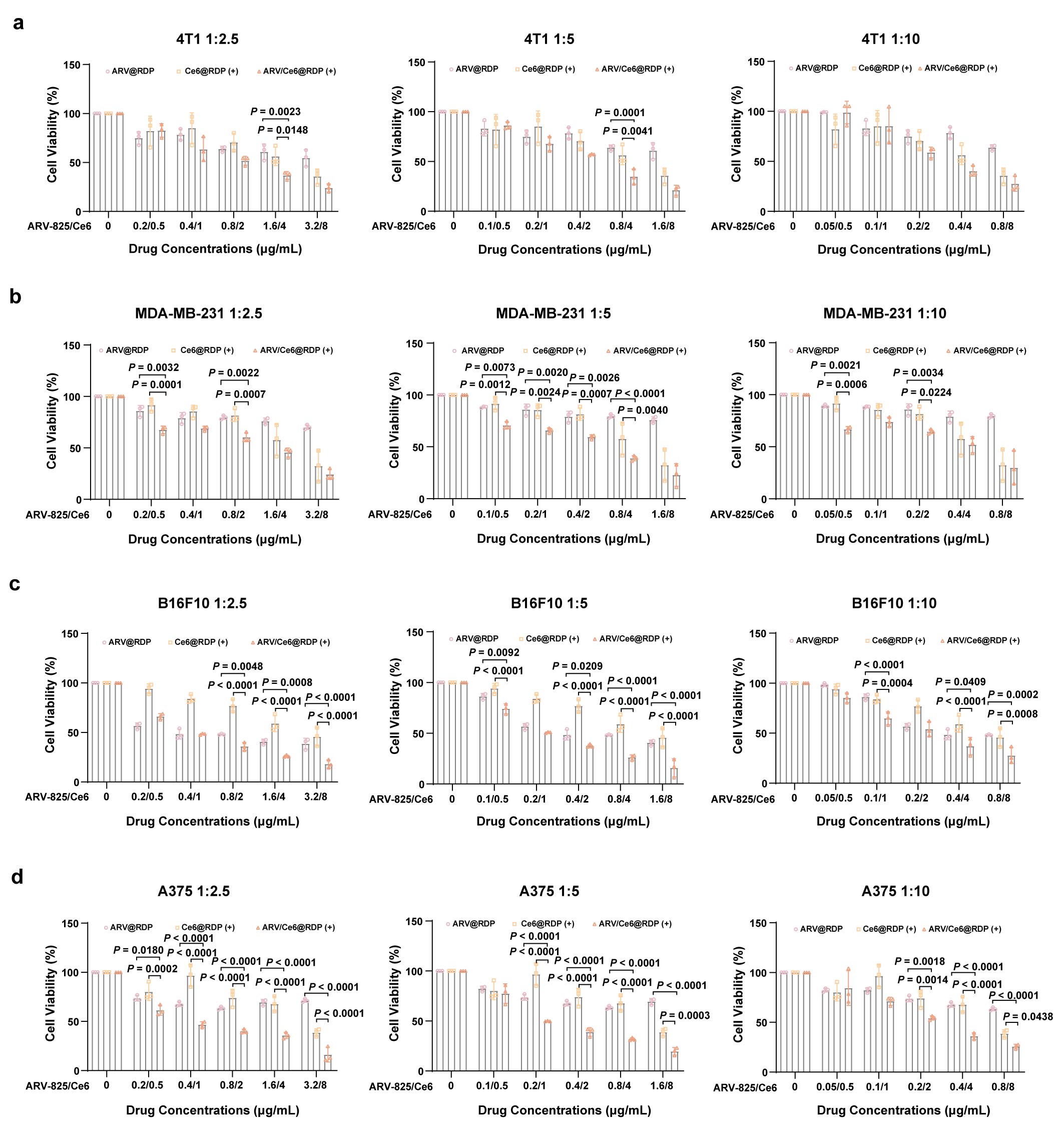
**

**Figure S11.**

**Cytotoxicity of ARV-825 and Ce6 combination at different drug ratios.** Cell viability assay of 4T1 (**a**), MDA-MB-231 (**b**), B16F10 (**c**) and A375 (**d**) cells after various treatments (n = 3 per group, data are shown as mean ± SDs, two-tailed unpaired Student’s *t* test).


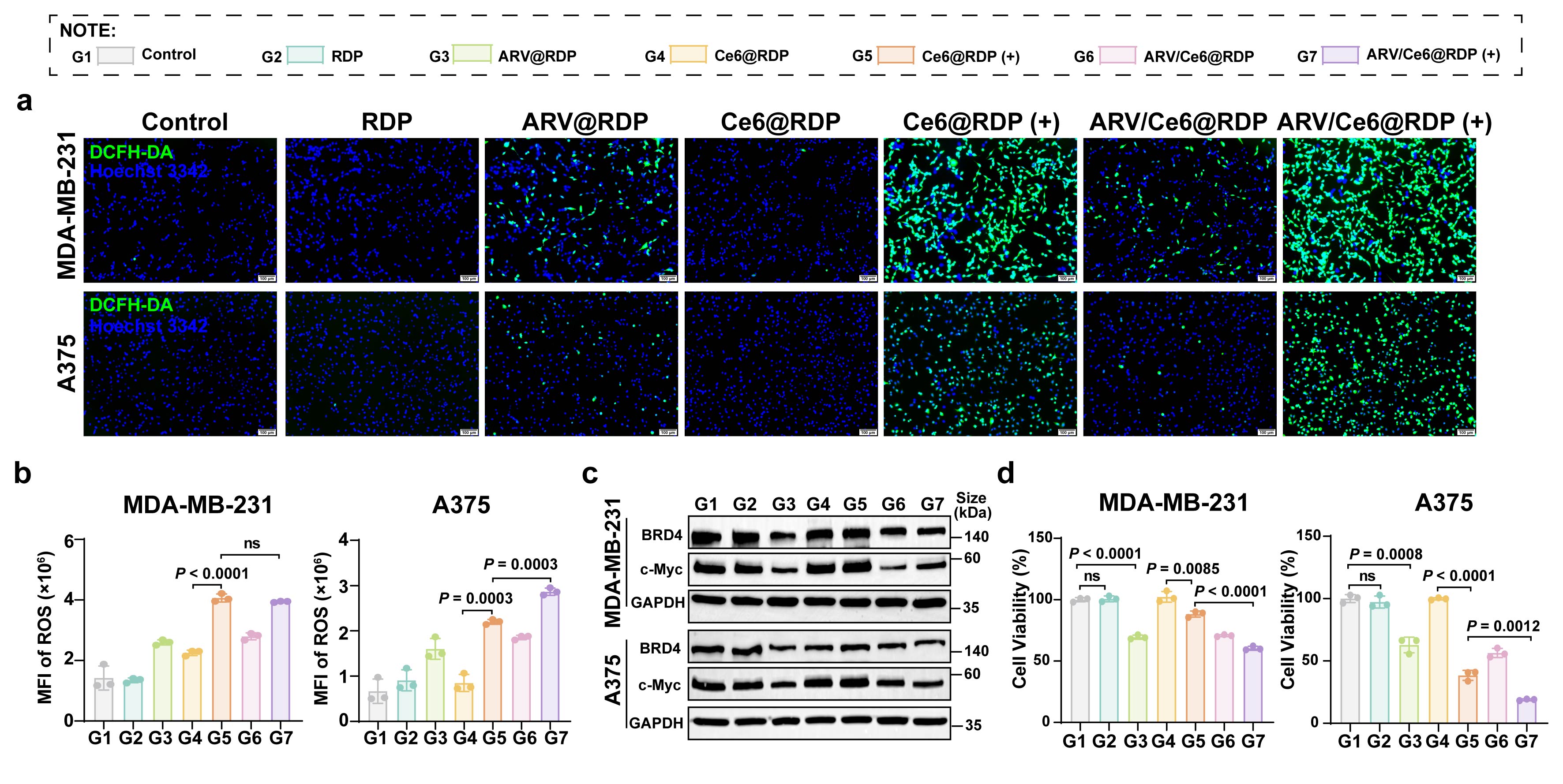


**Figure S12.**

**ROS generation and cytotoxicity**. (**a**) Representative ROS production images of MDA-MB-231 and A375 cells from different treatments (blue: nucleus; green: DCFH-DA labelling ROS). Scale bar: 100 µm. (**b**) Flow cytometric analyses for ROS production of MDA-MB-231 and A375 cells after various treatments (n = 3 per group, two-tailed unpaired Student’s *t* test). (**c**) Changes in BRD4 and c-Myc proteins of MDA-MB-231 and A375 cells following various treatments. (**d**) MTT cytotoxicity assay of various treatments in MDA-MB-231 and A375 cells (n = 3 per group, two-tailed unpaired Student’s *t* test). All data in this figure are shown as mean ± SDs.


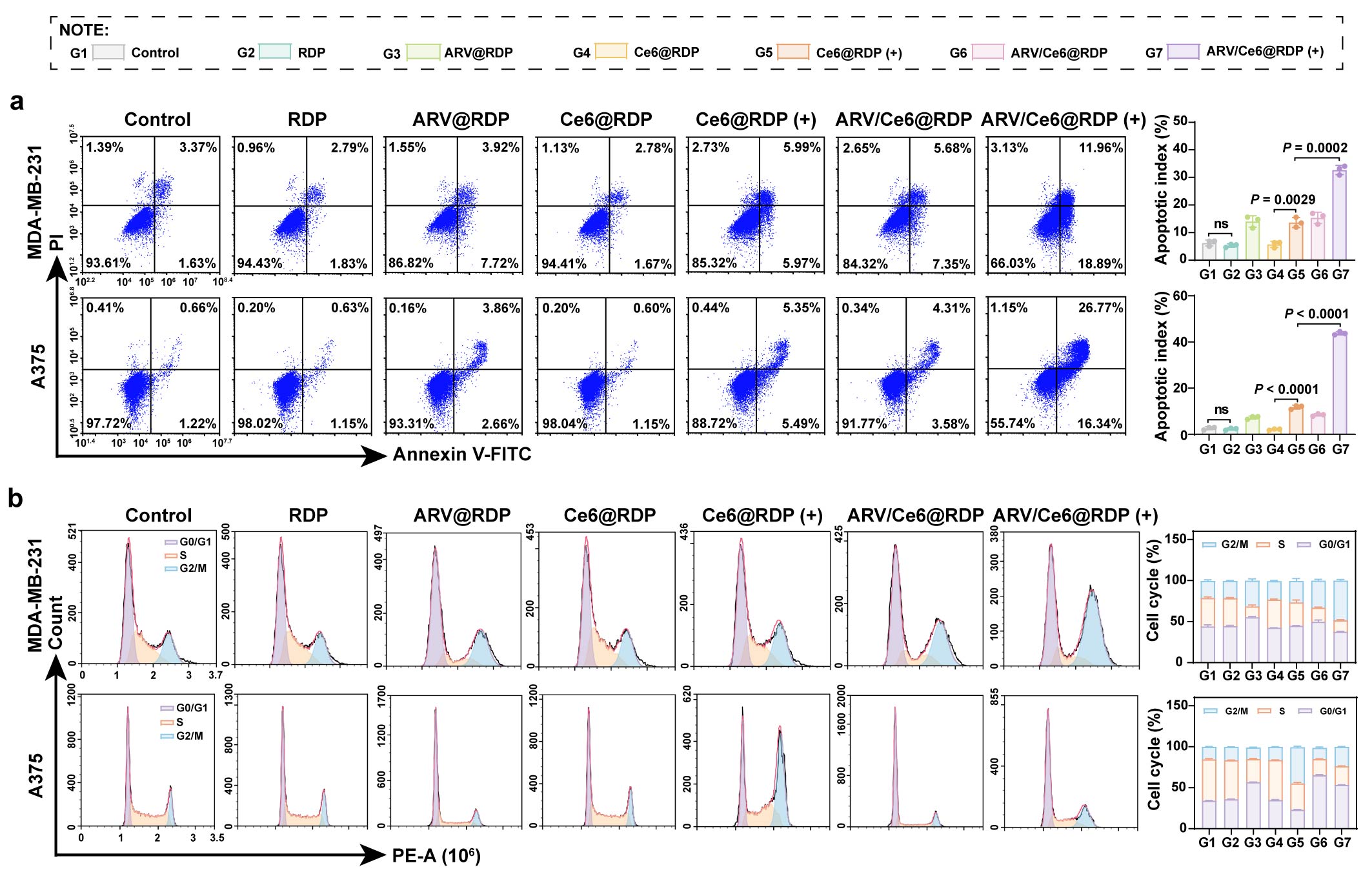


**Figure S13.**

**Analysis of various nanomedicine on cells apoptosis and cycle**. (**a**) Flow analysis of apoptosis in MDA-MB-231 and A375 cells after different treatments by Annexin V-FITC/PI double staining (n = 3 per group, two-tailed unpaired Student’s *t* test). (**b**) Flow analysis of cell cycles in MDA-MB-231 and A375 cells after different treatments by PI staining (n = 3 per group). All data in this figure are shown as mean ± SDs.


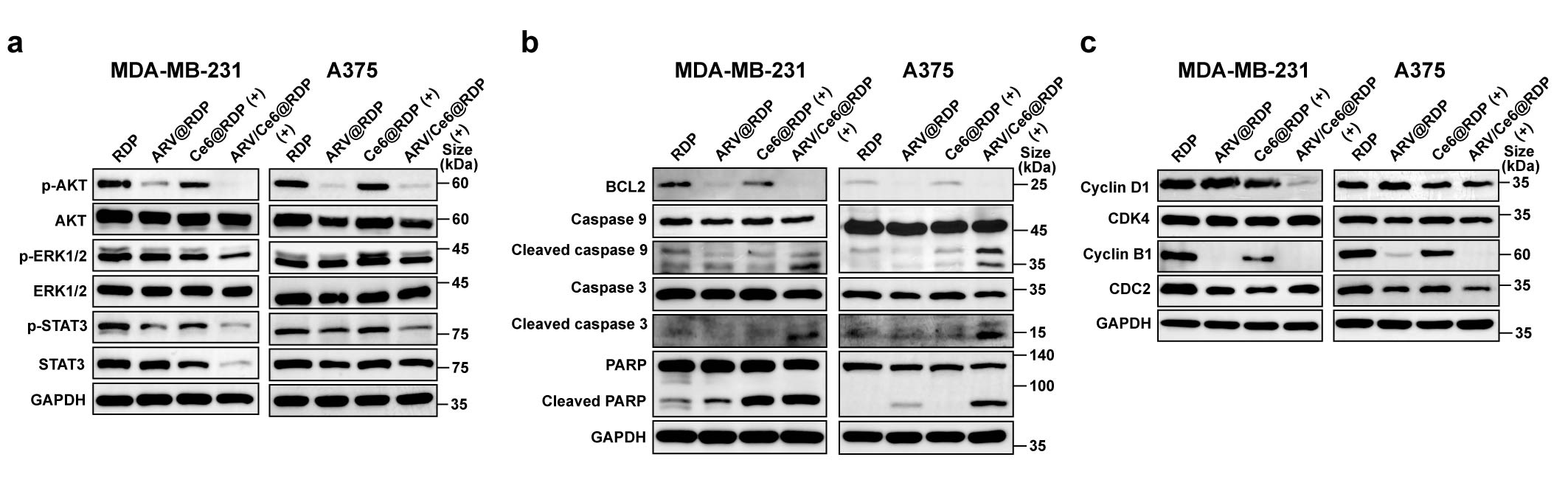


**Figure S14. Western blot analysis**.

Changes in proteins related to proliferation pathway (**a**), apoptosis pathway (**b**) and cell cycle (**c**) in MDA-MB-231 and A375 cells following different treatments.


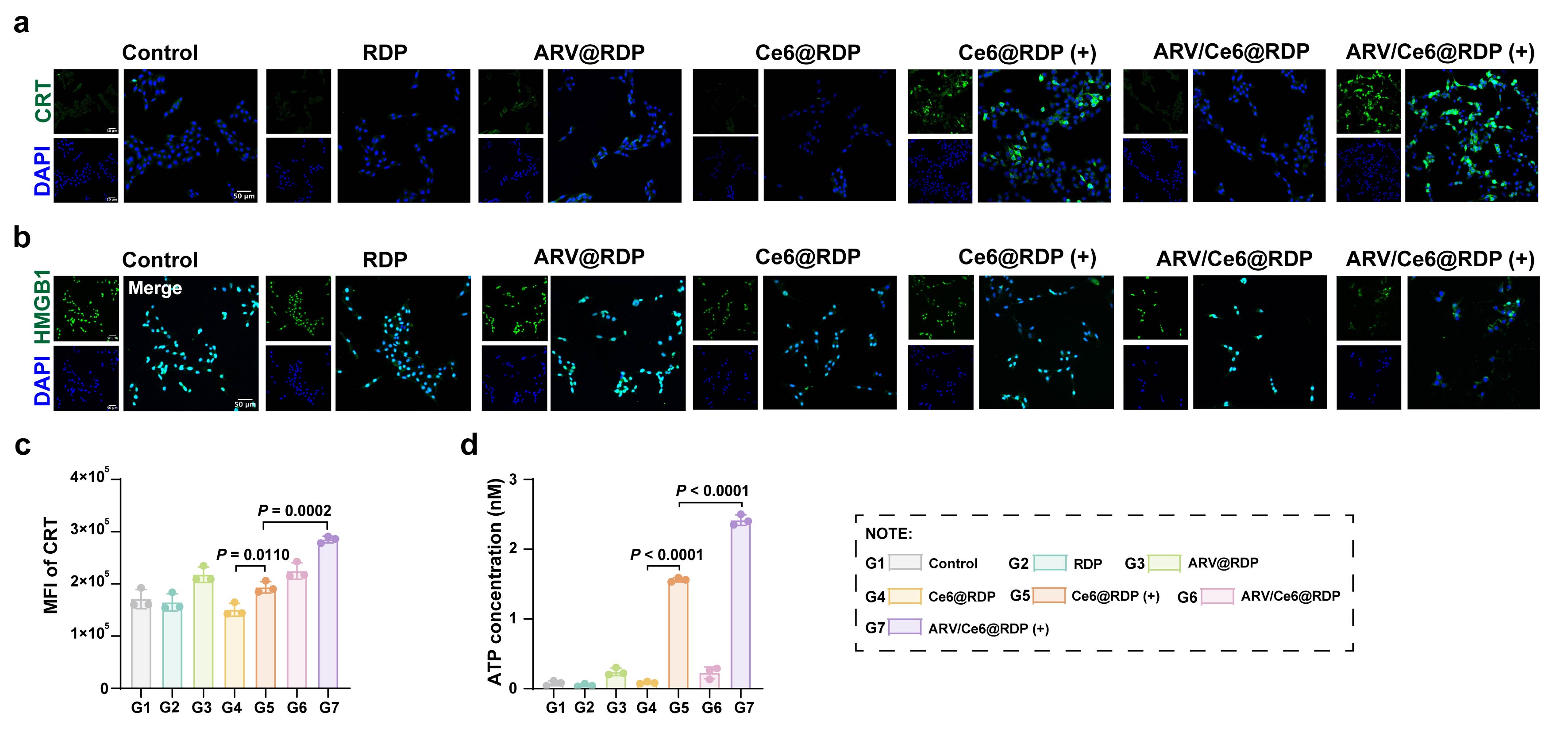


**Figure S15.**

**Detection of immunogenic cell death markers**. (**a**) CLSM examinations of CRT exposure in B16F10 cells induced by various treatments (blue: nucleus; green: CRT). Scale bar: 50 µm. (**b)** CLSM images of HMGB1 expression in B16F10 cells following different treatments (blue: nucleus; green: HMGB1). Scale bar: 50 µm. (**c**) Quantitative flow cytometry analysis of CRT exposure of B16F10 cells after different treatments (n = 3 per group, two-tailed unpaired Student’s *t* test). (**d**) Detection of ATP release from B16F10 cells receiving various treatments (n = 3 per group, two-tailed unpaired Student’s *t* test). All data in this figure are shown as mean ± SDs.


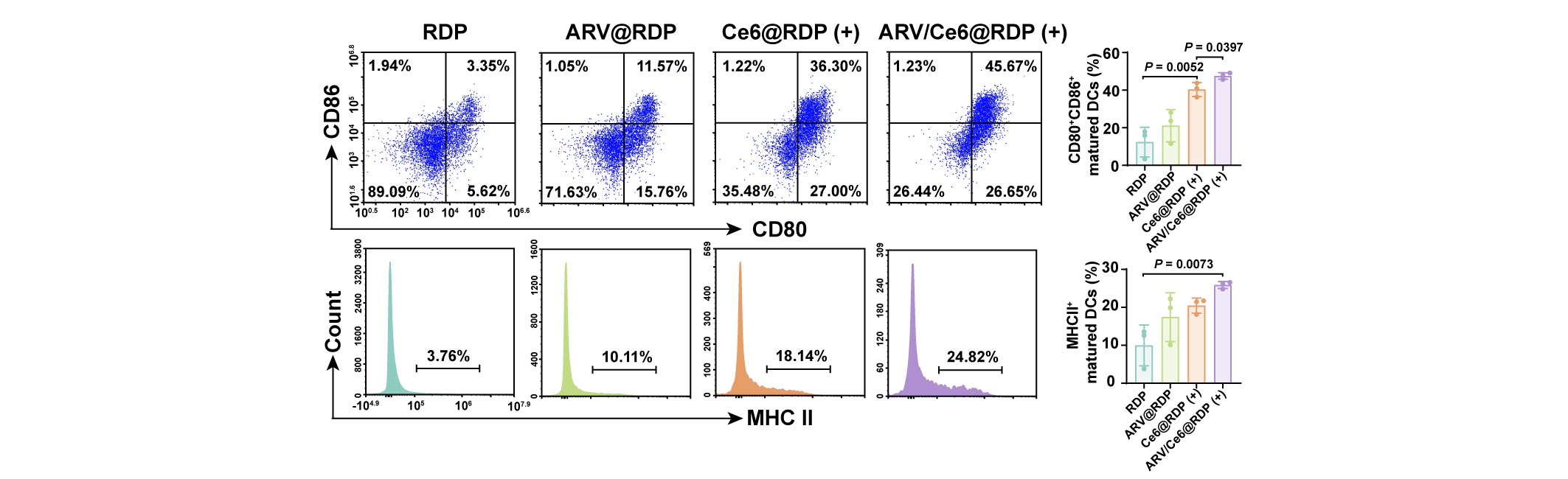


**Figure S16.**

**Maturation of DCs**. Flow cytometric analyses for matured DCs (CD11c^+^CD80^+^CD86^+^ and CD11c^+^MHCⅡ^+^) after co-incubation with B16F10 cells receiving various treatments (n = 3 per group, two-tailed unpaired Student’s *t* test). All data in this figure are shown as mean ± SDs.


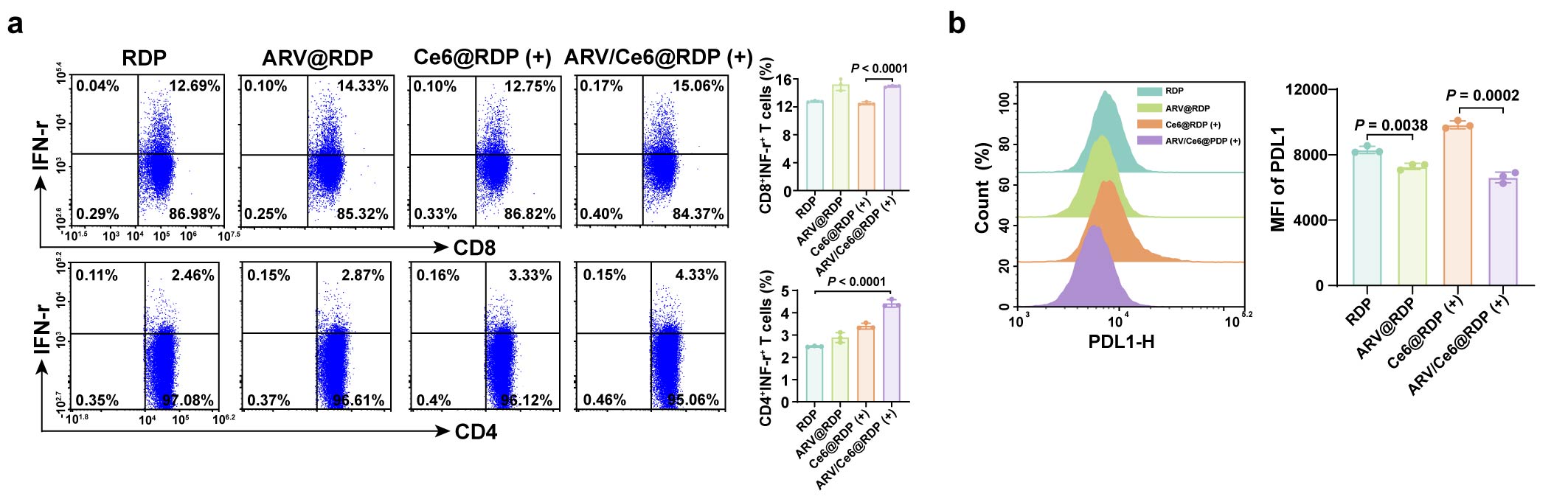


**Figure S17.**

**Activation of T cells and inhibition of PDL1**. (**a**) Flow cytometric analyses for T cells activation (CD3^+^CD8^+^IFN-γ^+^ and CD3^+^CD4^+^IFN-γ^+^) following co-incubation with matured DCs induced by B16F10 cells receiving various treatments (n = 3 per group, two-tailed unpaired Student’s *t* test). (**b**) Flow cytometric analyses for PDL1 expression in B16F10 cells following various treatments (n = 3 per group, two-tailed unpaired Student’s *t* test). All data in this figure are shown as mean ± SDs.


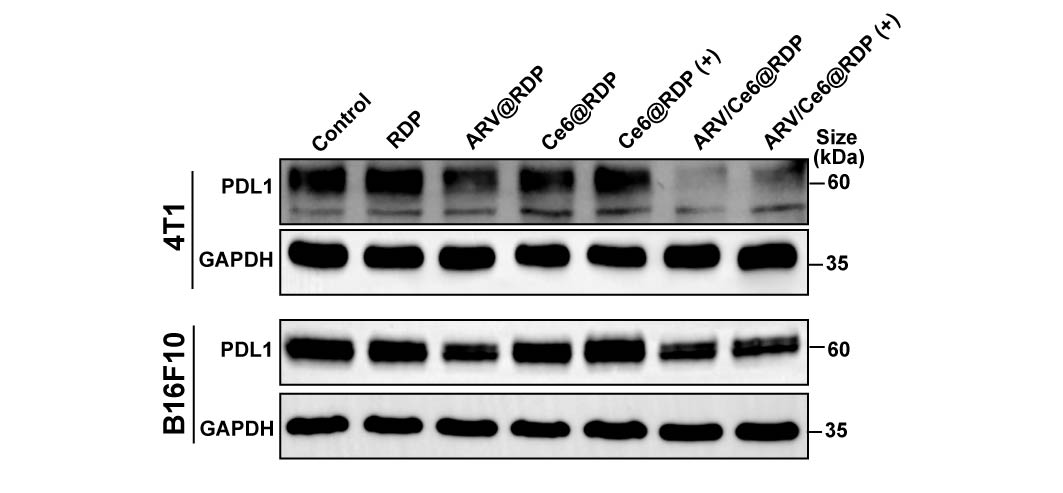


**Figure S18.**

**Changes in PDL1 protein of 4T1 and B16F10 cells following various treatments**.


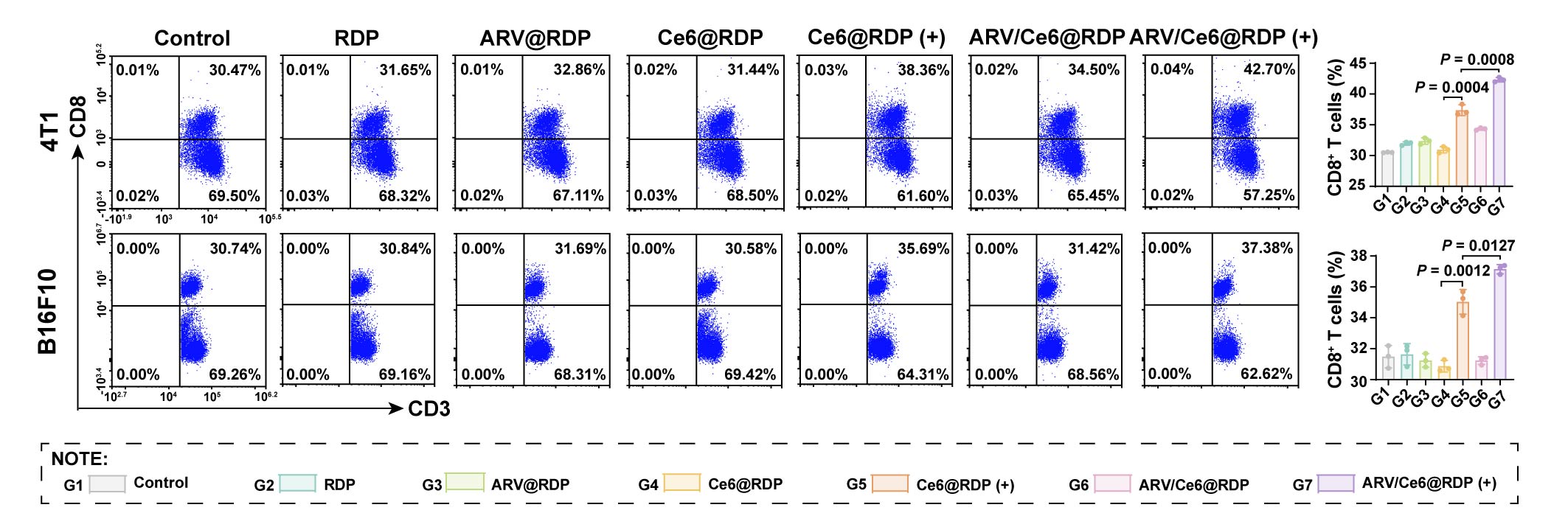


**Figure S19.**

**Flow cytometric analyses for CD3^+^CD8^+^ T cells population after co-incubation with 4T1 or B16F10 cells subjected to various treatments** (n = 3 per group, two-tailed unpaired Student’s *t* test). All data in this figure are shown as mean ± SDs.


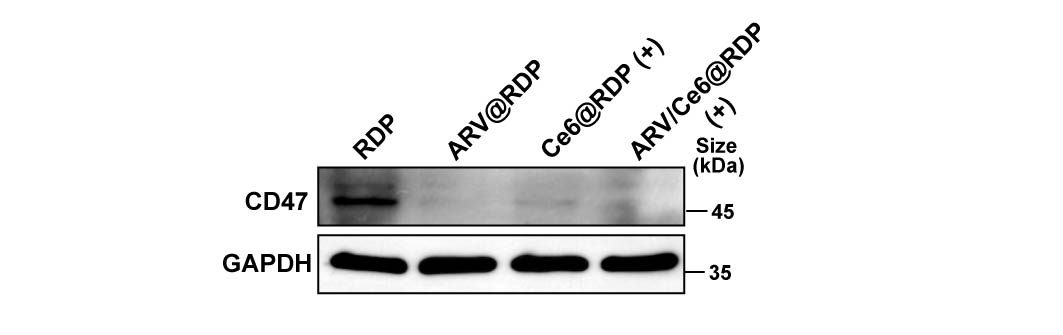


**Figure S20.**

**Changes in CD47 protein of B16F10 cells after different treatments**.


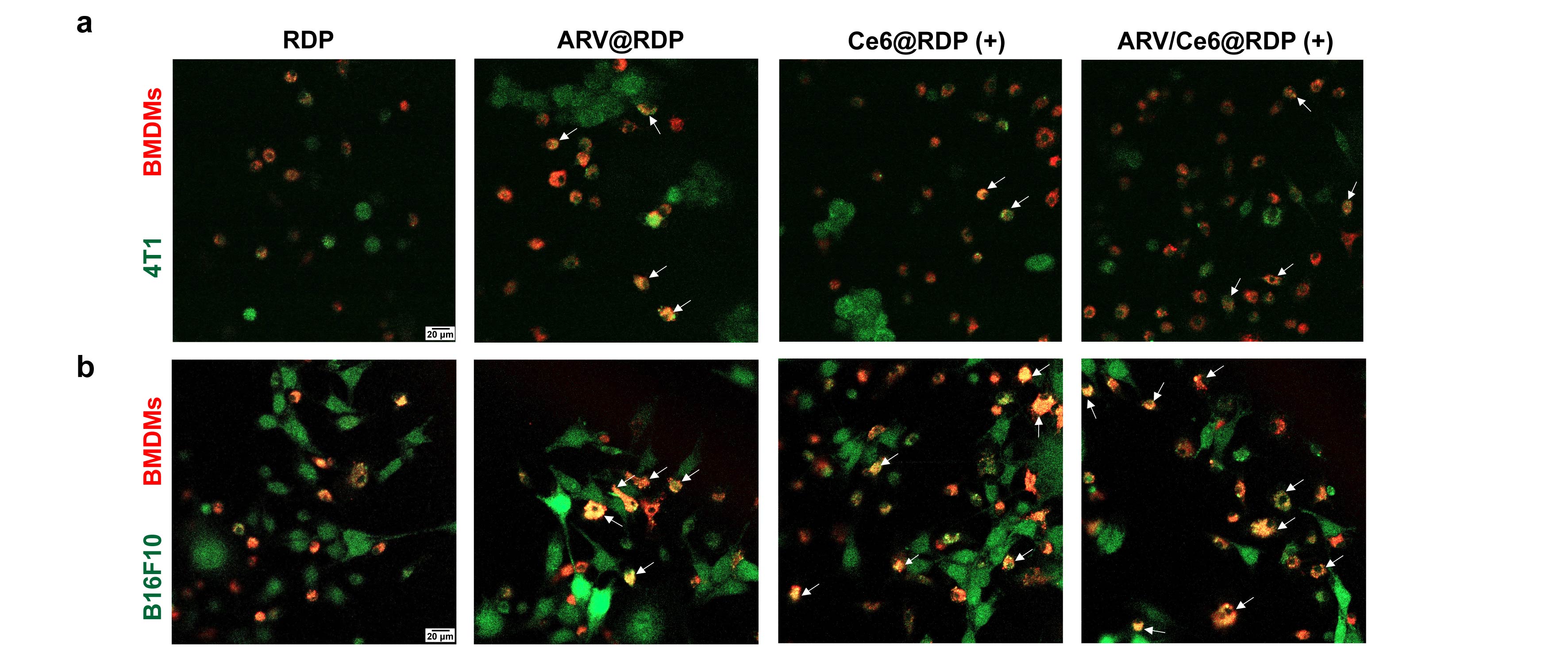


**Figure S21.**

**Representative CLSM images of in vitro phagocytosis of 4T1** (**a**) **and B16F10** (**b**) **cells by BMDMs**. Tumor cells were stained with CFSE (green). BMDMs were stained with DiD (red). Scale bar: 20 µm.


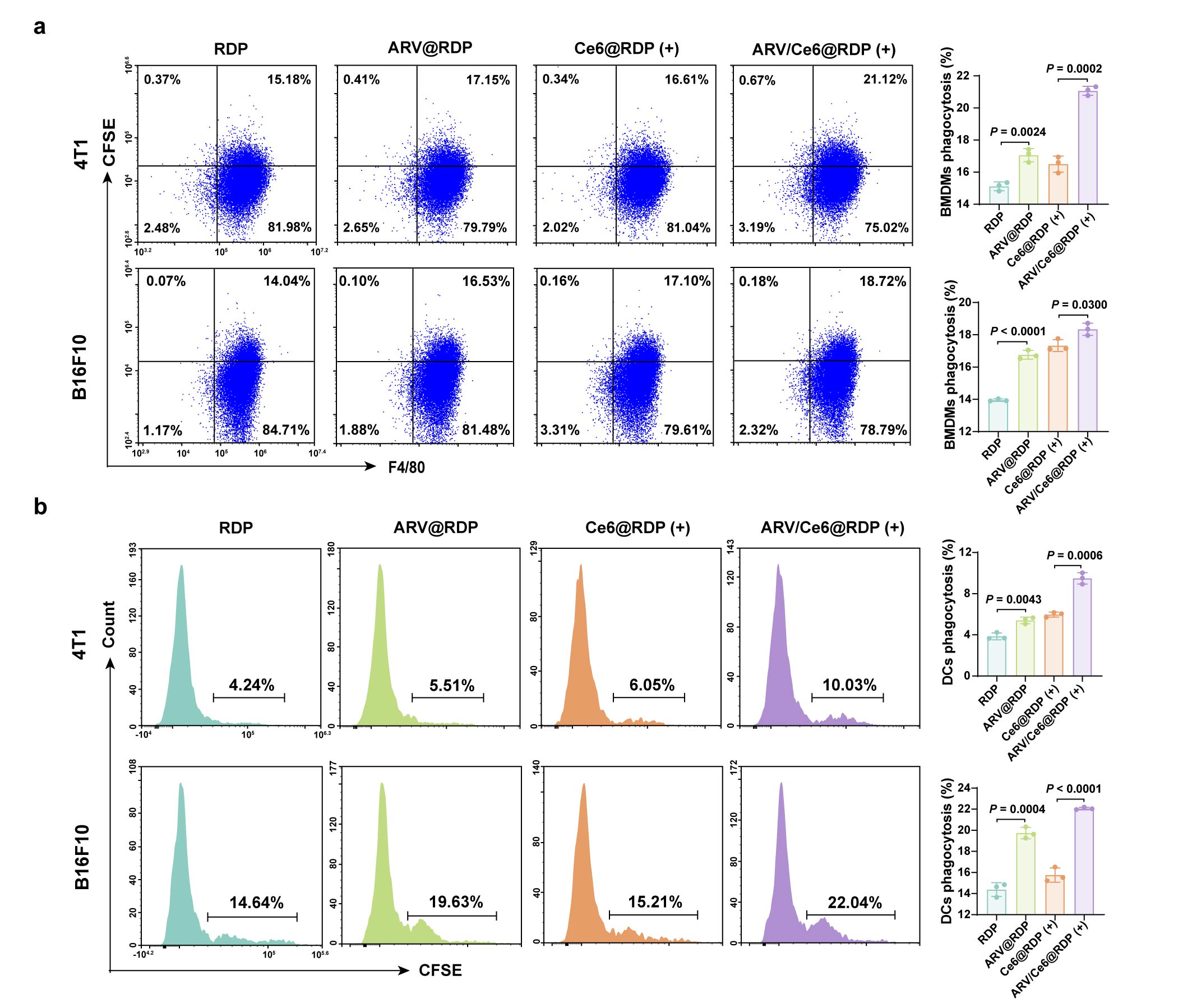


**Figure S22.**

**BMDMs or BMDCs phagocytosis of tumor cells detected by flow cytometry**. (**a**) Flow cytometric analyses for BMDMs phagocytosis of 4T1 and B16F10 cells receiving various treatments (n = 3 per group, two-tailed unpaired Student’s *t* test). (**b**) Flow cytometric analyses for BMDCs phagocytosis of 4T1 and B16F10 cells following various treatments (n = 3 per group, two-tailed unpaired Student’s *t* test). All data in this figure are shown as mean ± SDs.


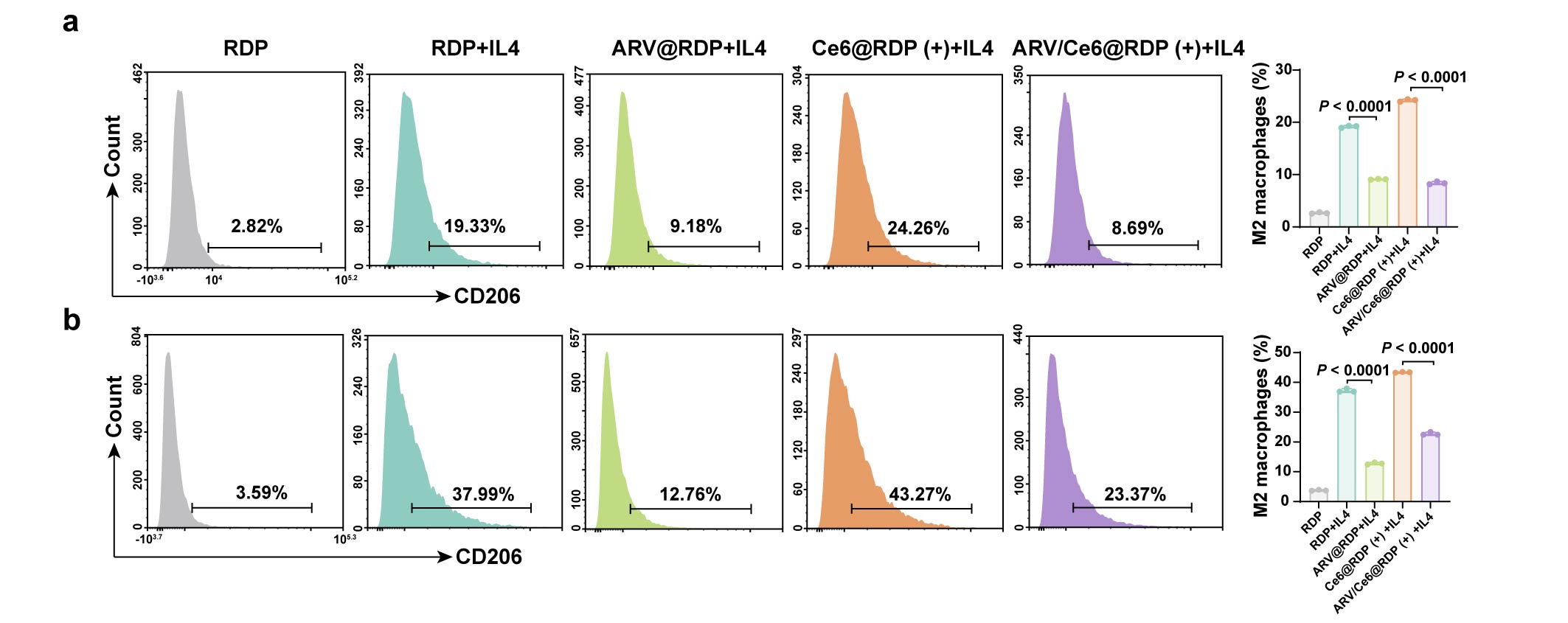


**Figure S23.**

**Macrophage M2 polarization analysis**. Flow cytometric analyses for M2 polarization of BMDMs from BALB/c (a) and C57BL/6 (b) mice after different treatments (n = 3 per group, two-tailed unpaired Student’s *t* test). All data in this figure are shown as mean ± SDs.


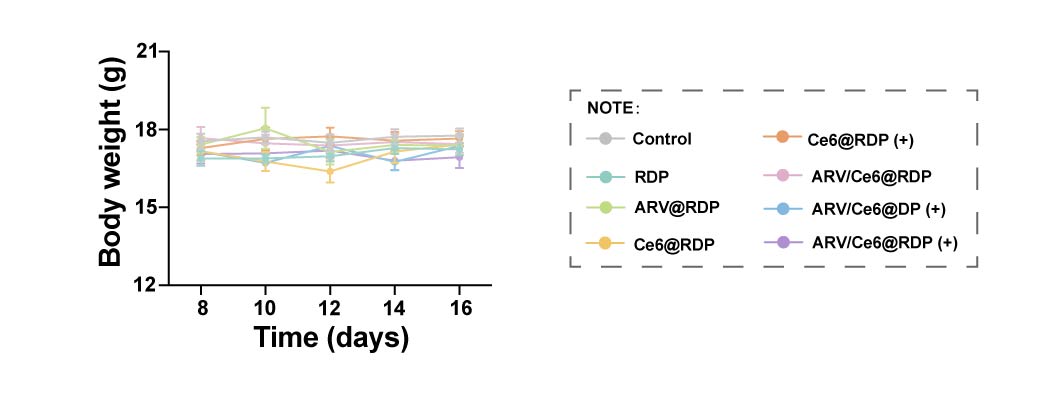


**Figure S24.**

**Change in** **body weight of the 4T1 tumor-bearing mice in each group** (n = 6 per group, data are shown as mean ± SEMs).


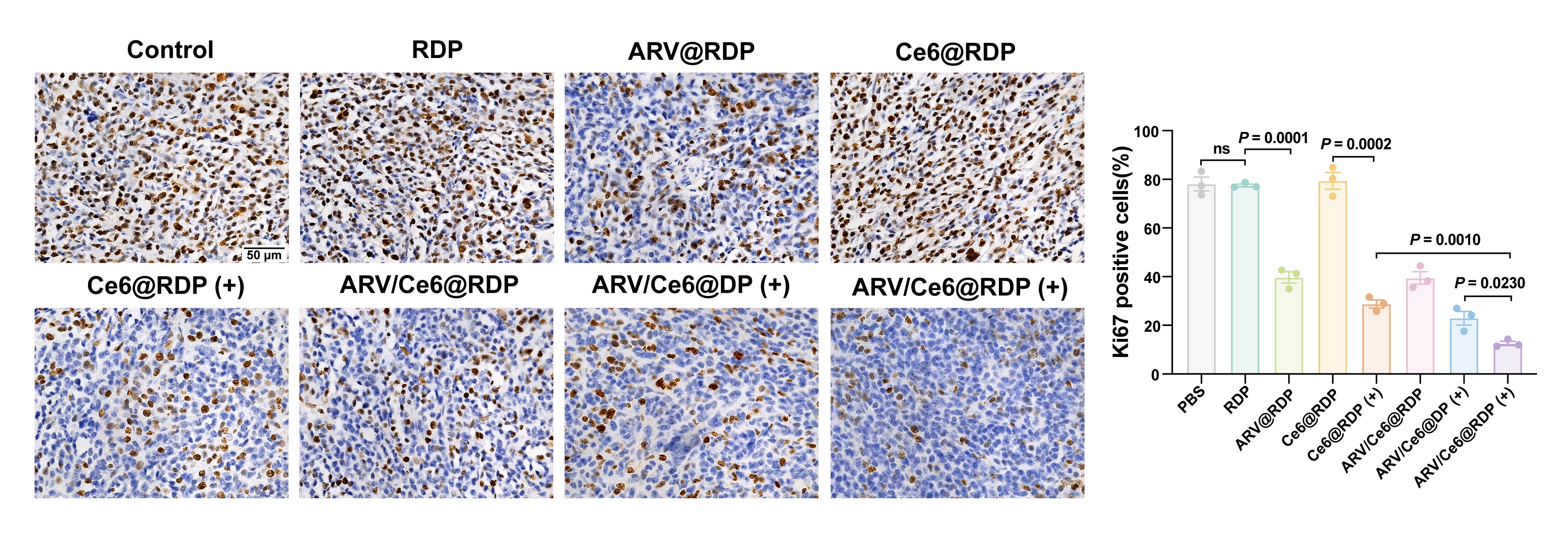


**Figure S25. Representative Ki67 IHC images and quantitative analysis of 4T1 tumor sections from mice receiving different treatments** (n = 3 per group, two-tailed unpaired Student’s *t* test, ns: not significant). Data are shown as mean ± SEMs. Scale bar: 50 µm.


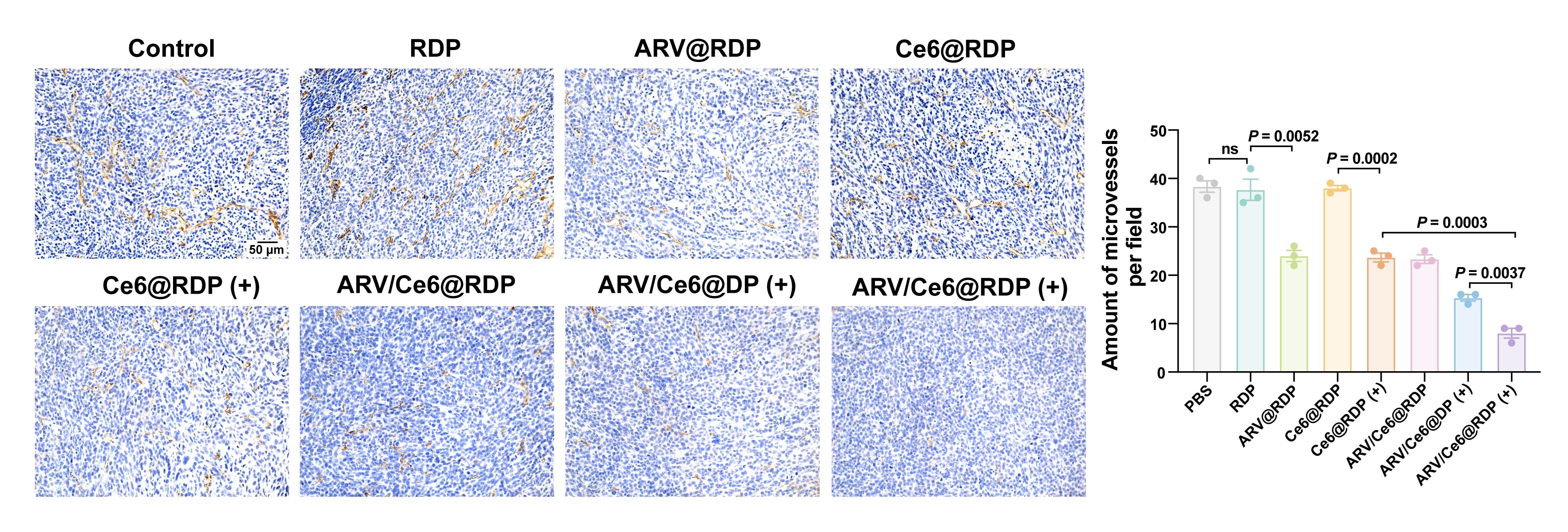


**Figure S26.**

**IHC staining of CD31 proteins and quantitative analysis in 4T1 tumor tissues from mice receiving various treatments** (n = 3 per group, two-tailed unpaired Student’s *t* test, ns: not significant). Data are shown as mean ± SEMs. Scale bar: 50 µm.


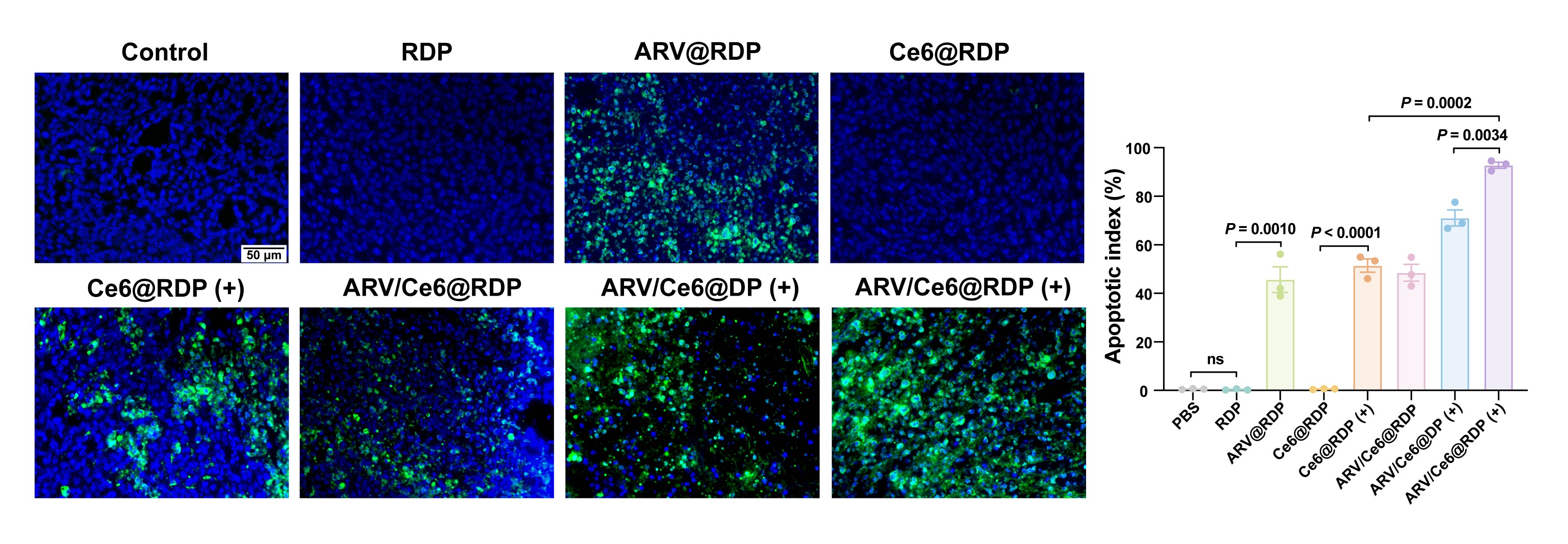


**Figure S27.**

**Representative TUNEL staining images and quantitative analysis of 4T1 tumor sections from mice receiving different treatments** (n = 3 per group, two-tailed unpaired Student’s *t* test, ns: not significant). Data are shown as mean ± SEMs. Scale bar: 50 µm.


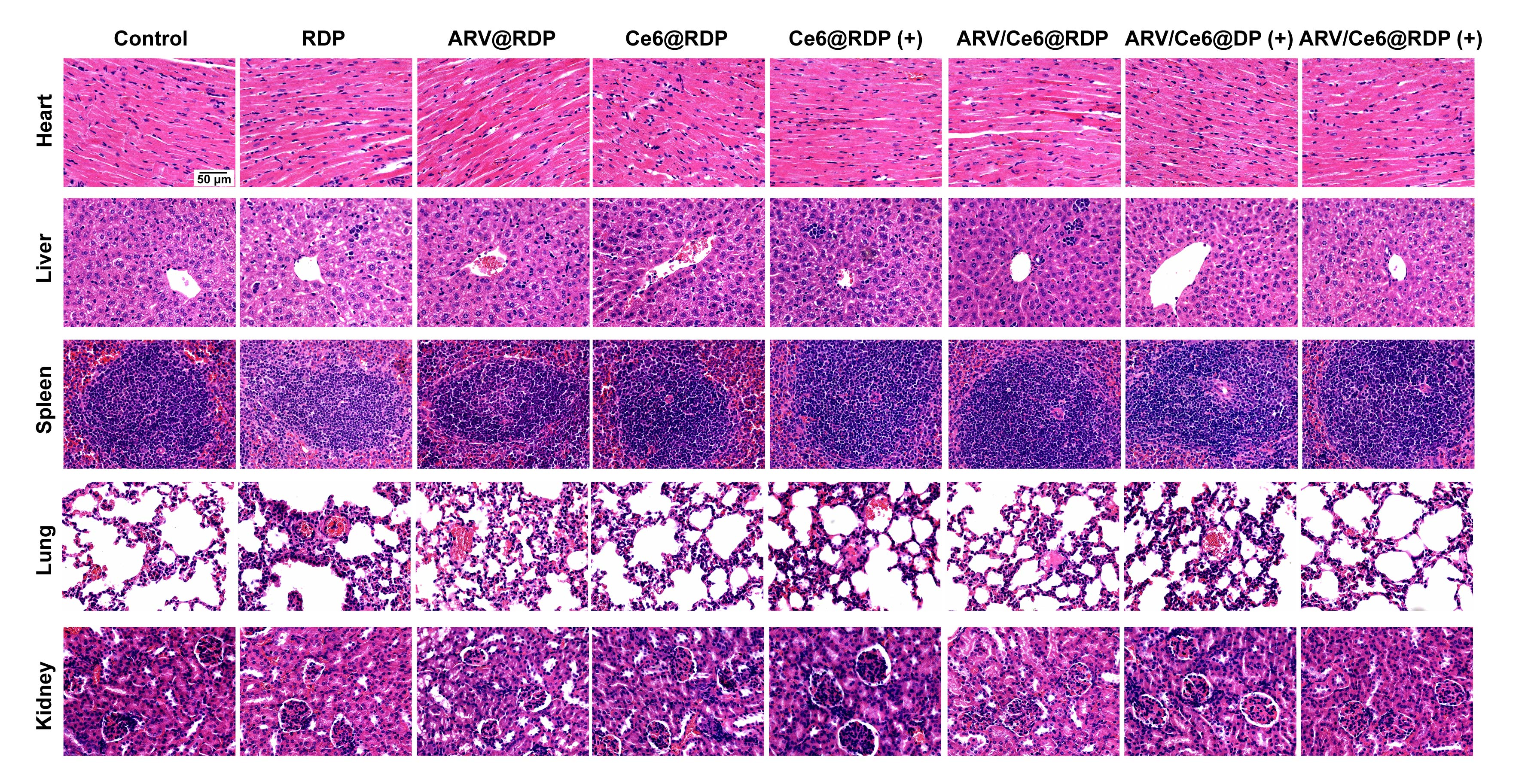


**Figure S28.**

**Representative H****&E staining of heart, liver, spleen, lung and kidney in 4T1 tumor-bearing mice after different treatments**. Scale bar: 50 µm.


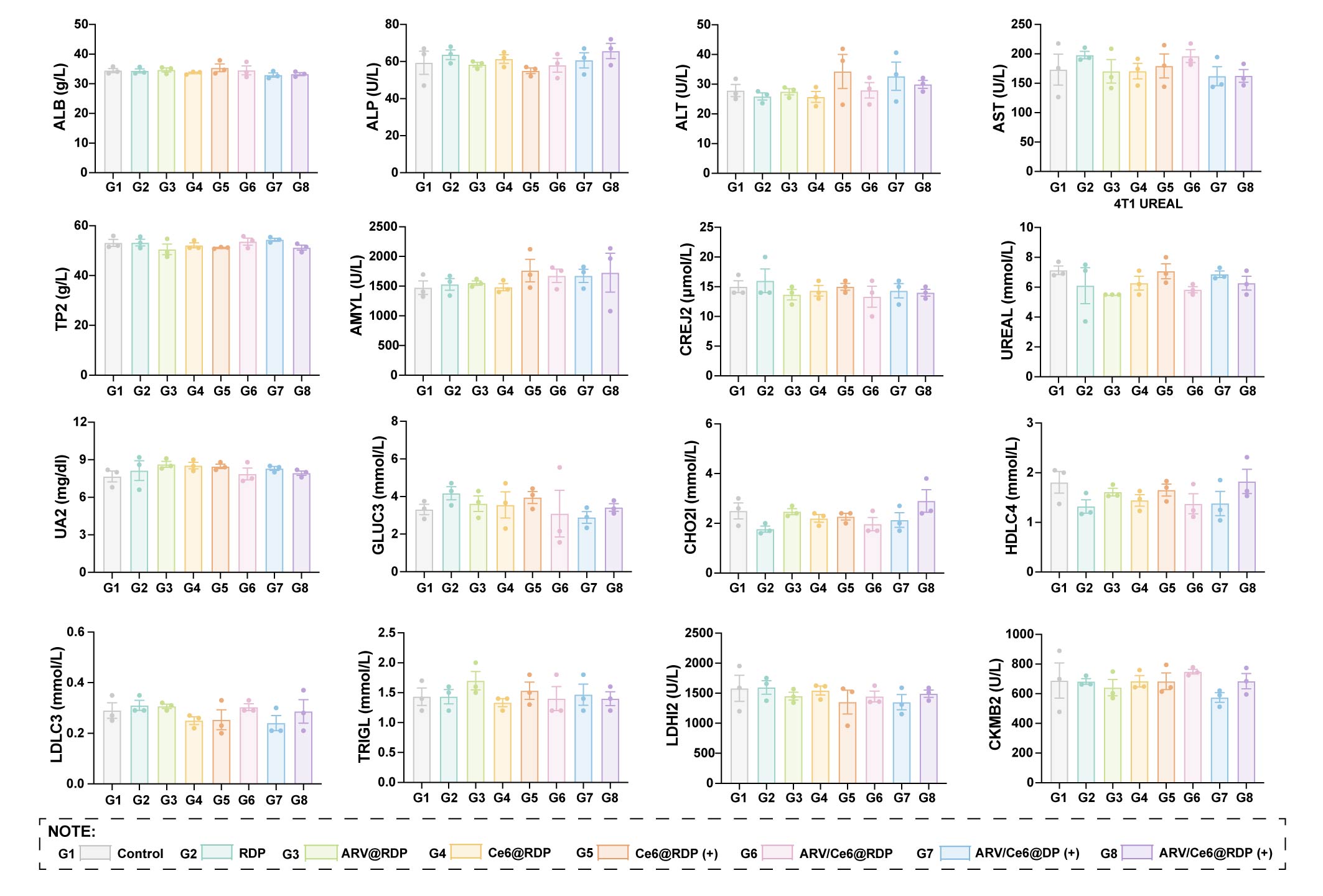


**Figure S29.**

**Blood biochemical indicators analysis of** **4T1** **tumor-bearing mice after various treatments** (n = 3 per group). Data are shown as mean ± SEMs.


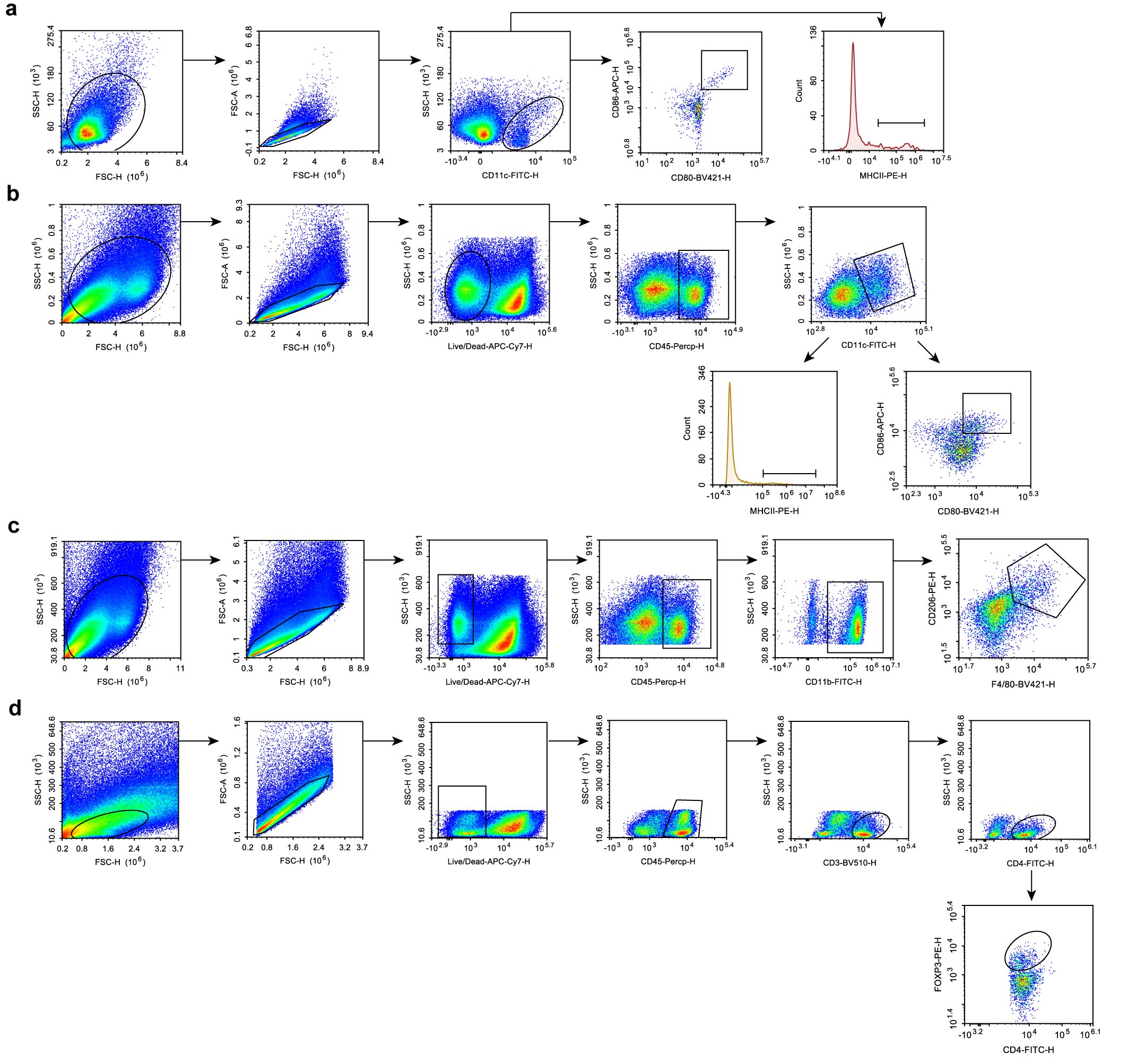


**Figure S30.**

**Flow cytometry gating strategies for Figure 5j-o in 4T1 tumor-bearing mice.** (**a**) Gating strategy for CD11c^+^CD80^+^CD86^+^ DCs and CD11c^+^MHCⅡ^+^ DCs in tumor-draining lymph nodes. (**b**) Gating strategy for L/D^-^CD45^+^CD11c^+^CD80^+^CD86^+^ DCs and L/D^-^CD45^+^CD11c^+^MHCⅡ^+^ DCs in tumor tissues. (**c**) Gating strategy for L/D^-^CD45^+^CD11b^+^F4/80^+^CD206^+^ macrophages in tumor tissues. (**d**) Gating strategy for L/D^-^CD45^+^CD3^+^CD4^+^Foxp3^+^ Tregs in tumor tissues.


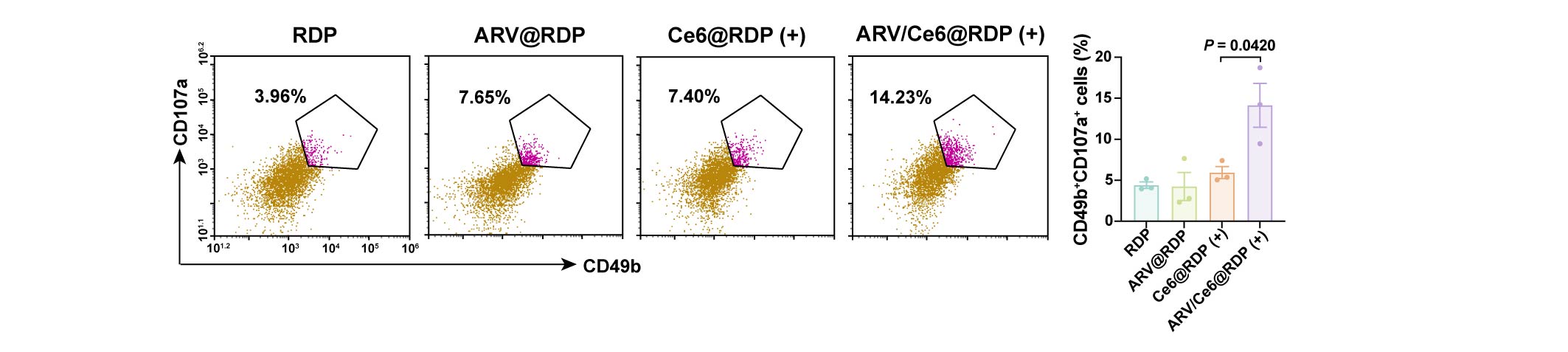


**Figure S31.**

**Flow cytometrical analysis of activated NK cells (L/D^-^CD45^+^CD3^-^CD49b^+^CD107a^+^) in tumor tissues from 4T1 tumor-bearing mice receiving different treatments** (n = 3 per group, two-tailed unpaired Student’s *t* test). Data are shown as mean ± SEMs.


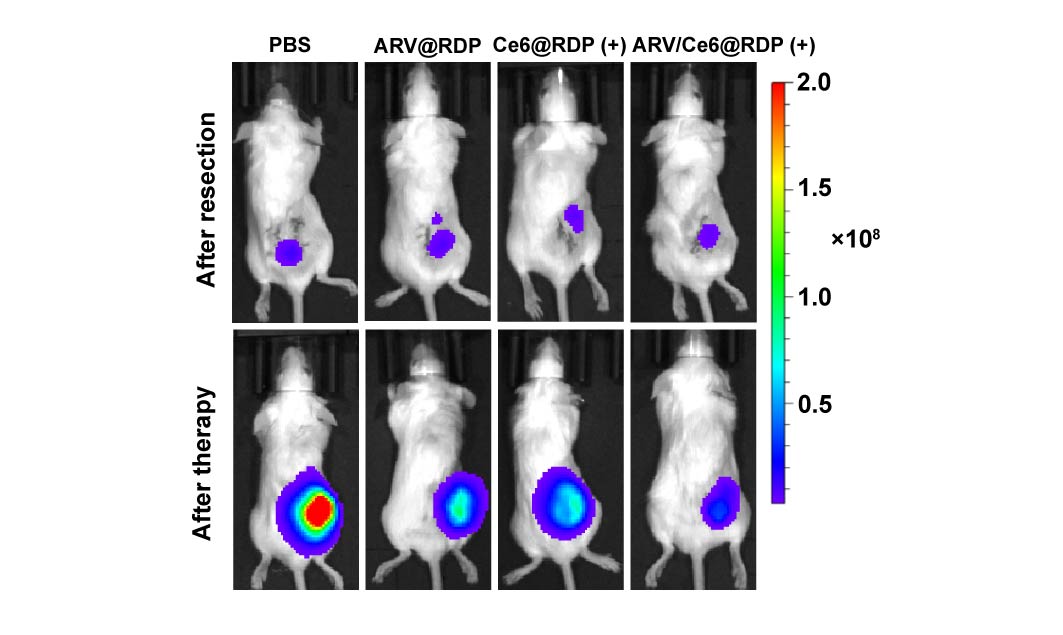


**Figure S32.**

**In vivo imaging of 4T1 subcutaneous tumor-bearing mice after surgery and treatment**.


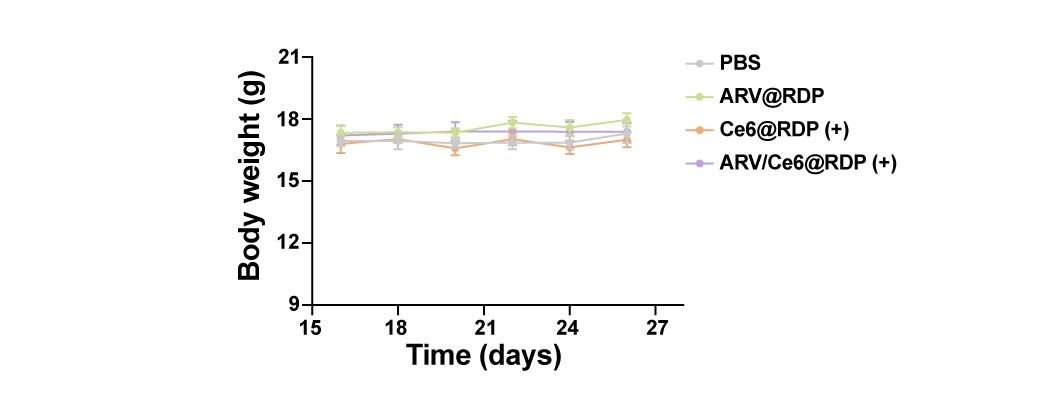


**Figure S33.**

**Body weight fluctuation curves of** **postoperative 4T1 tumor-bearing mice during treatments** (n = 6 per group, data are shown as mean ± SEMs).


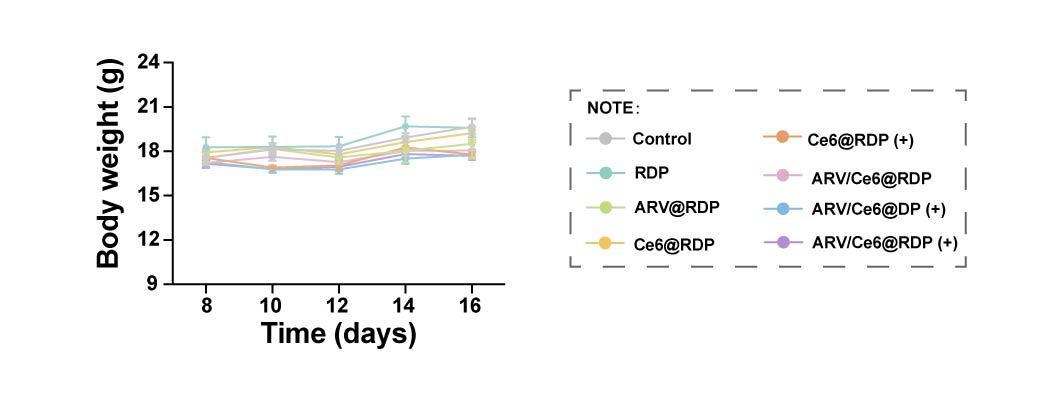


**Figure S34.**

**Body weight change curves of B16F10 tumor-bearing mice upon various treatments** (n = 6 per group, data are shown as mean ± SEMs).


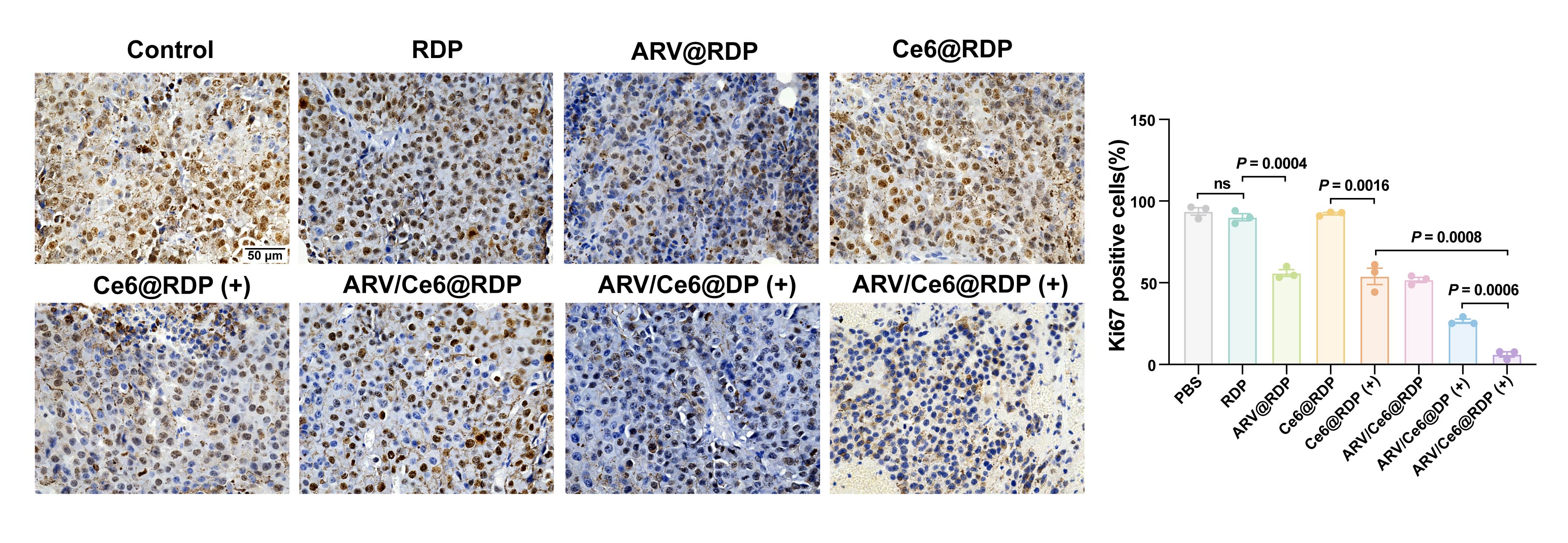


**Figure S35.**

**IHC staining of Ki67 in B16F10 tumor tissues after various treatments** (n = 3 per group, two-tailed unpaired Student’s *t* test, ns: not significant). Data are shown as mean ± SEMs. Scale bar: 50 µm.


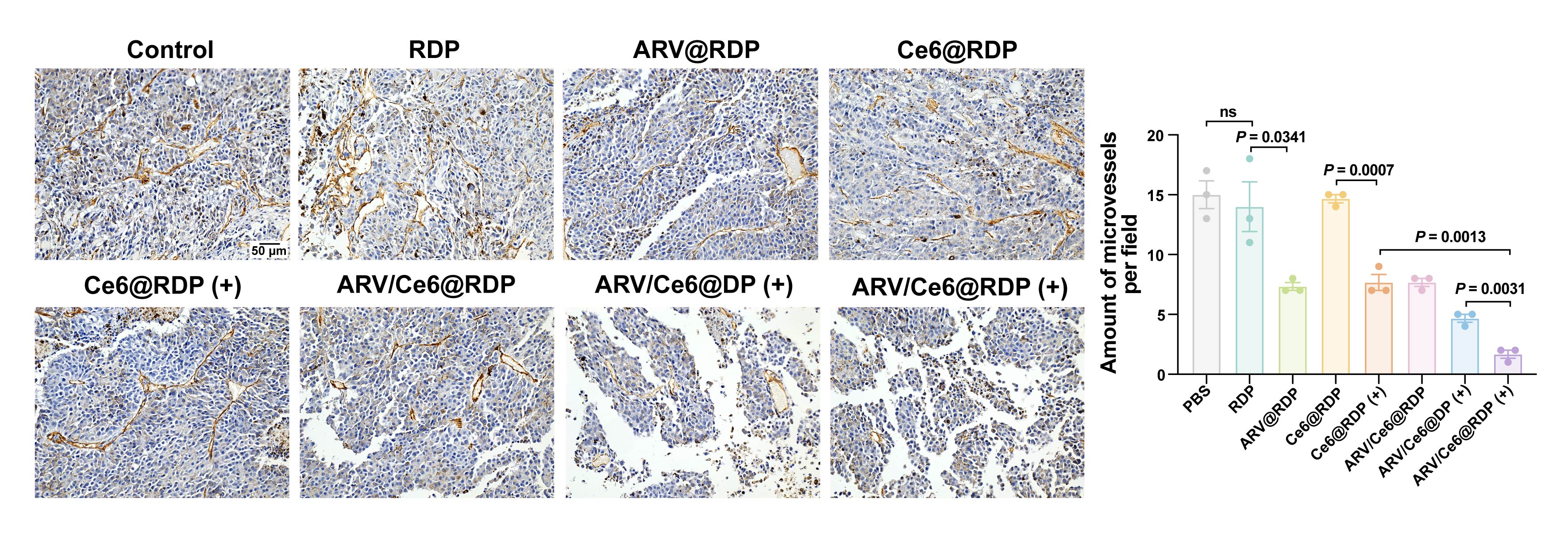


**Figure S36.**

**CD31 staining of tumor sections from B16F10 tumor-bearing mice receiving different treatments** (n = 3 per group, two-tailed unpaired Student’s *t* test, ns: not significant). Data are shown as mean ± SEMs. Scale bar: 50 µm.


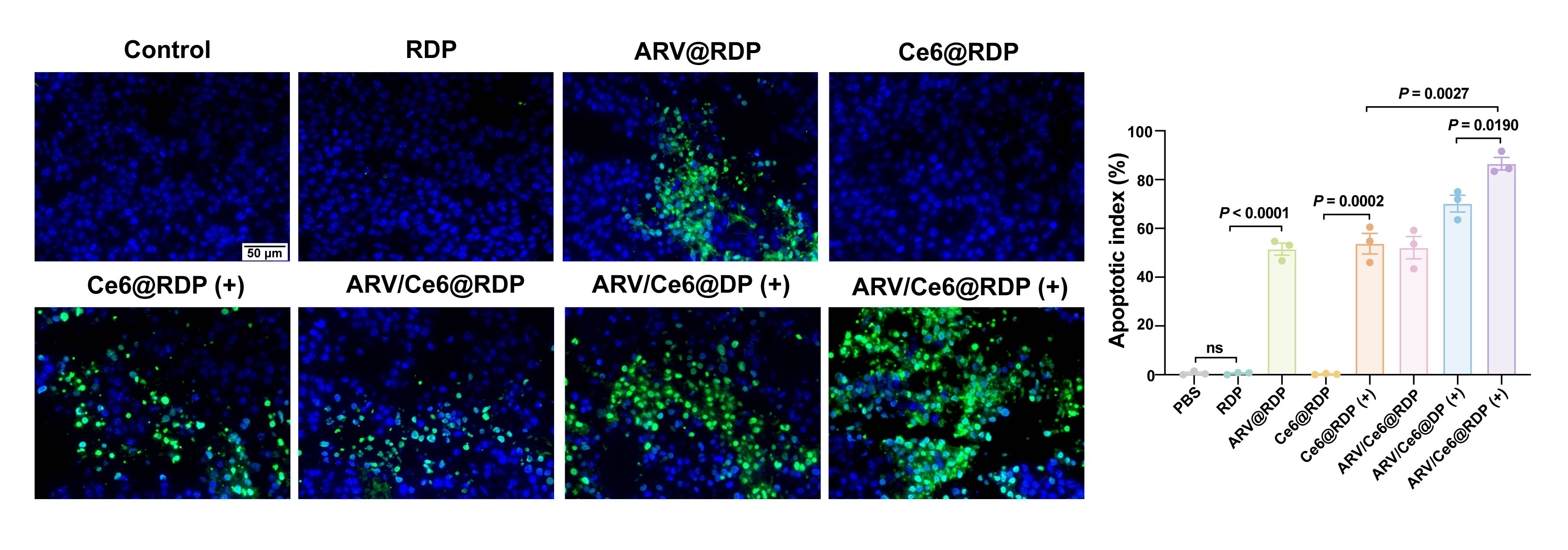


**Figure S37.**

**TUNEL staining of B16F10 tumor sections upon different treatments**. (n = 3 per group, two-tailed unpaired Student’s *t* test, ns: not significant). Data are shown as mean ± SEMs. Scale bar: 50 µm.


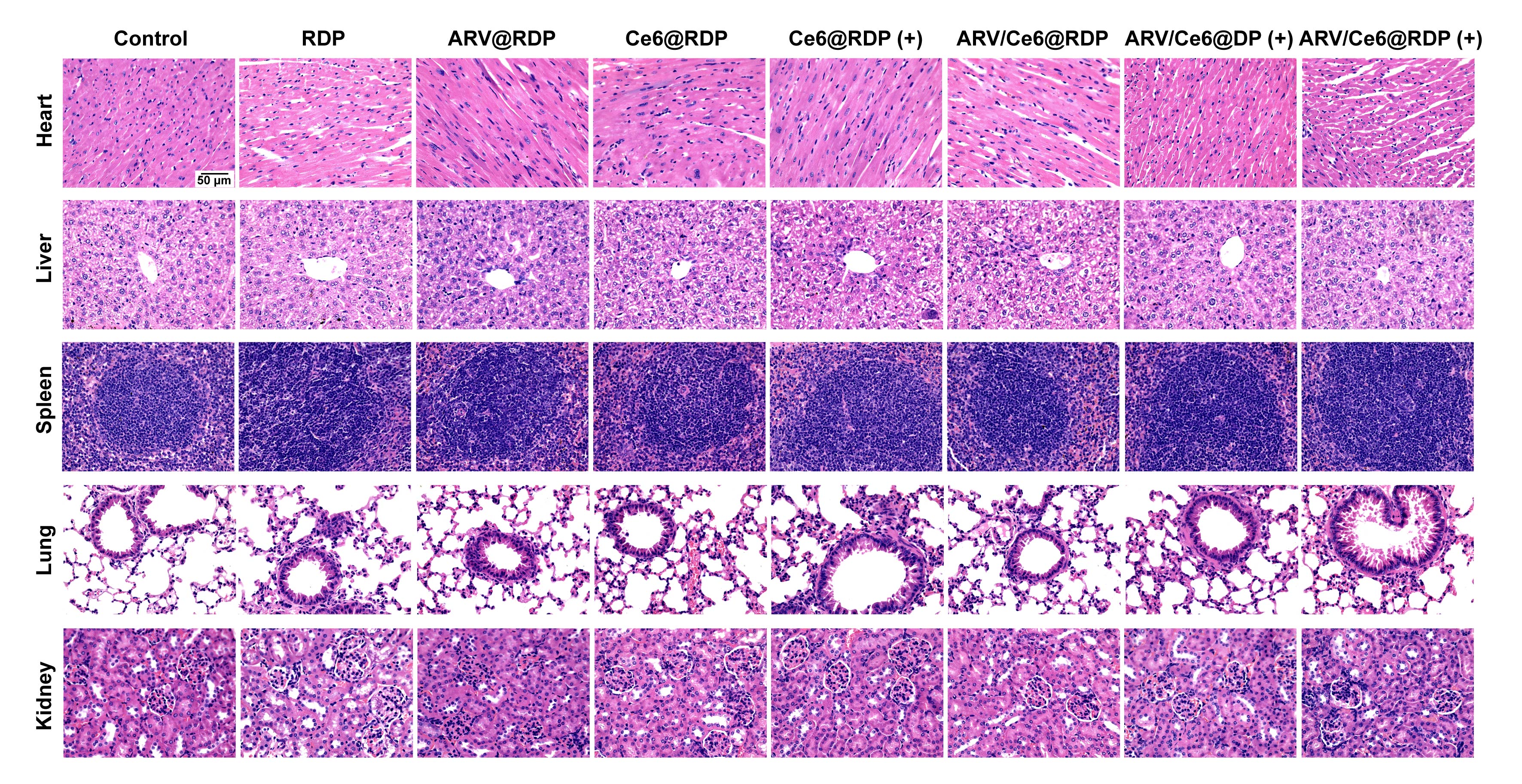


**Figure S38.**

**Representative pictures of H&E staining of heart, liver, spleen, lung and kidney from B16F10 tumor-bearing mice receiving different treatments**. Scale bar: 50 µm.


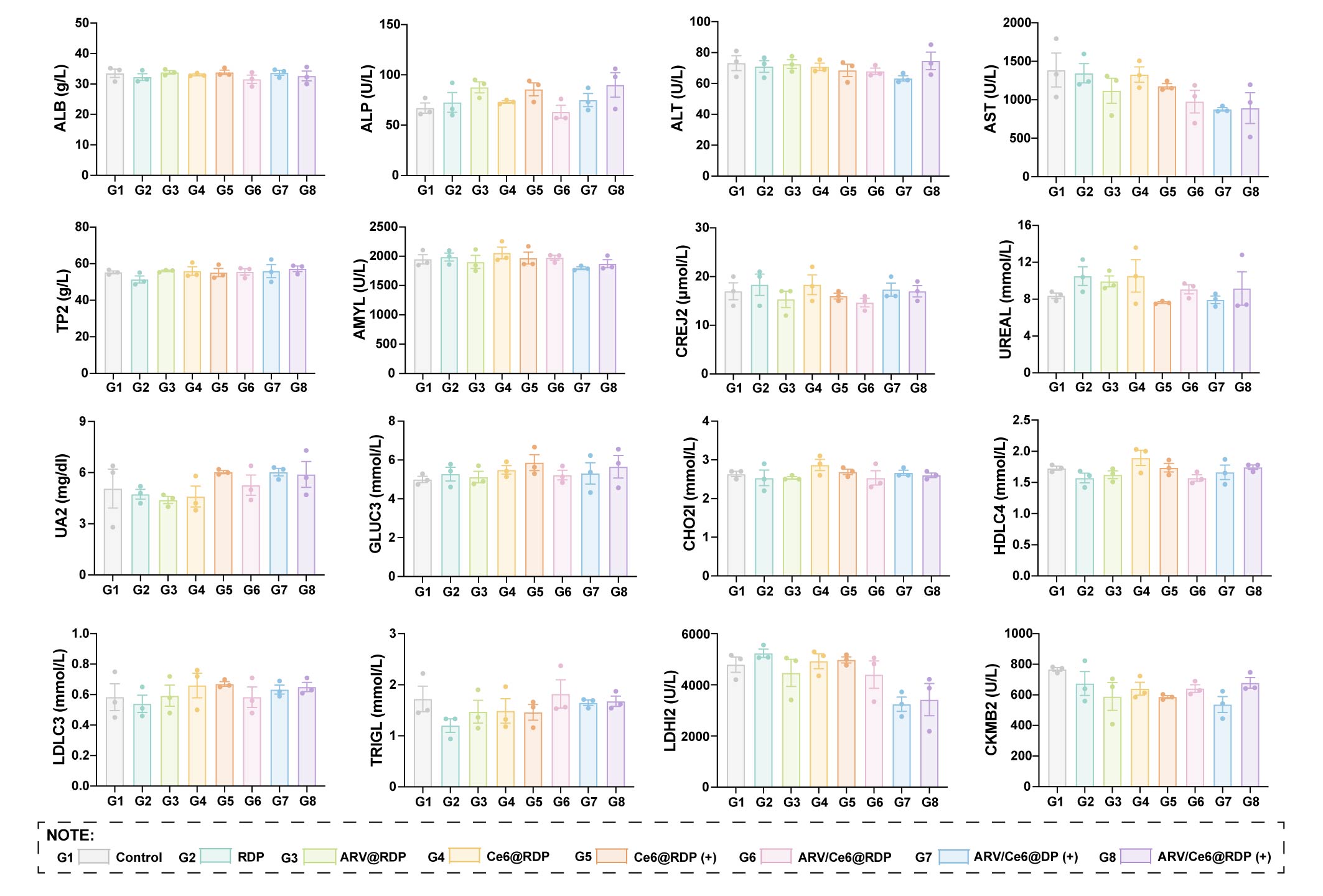


**Figure S39.**

**Levels of main blood biochemical indexes in serum from B16F10 tumor-bearing mice treated with different formulations** (n = 3 per group). Data are shown as mean ± SEMs.


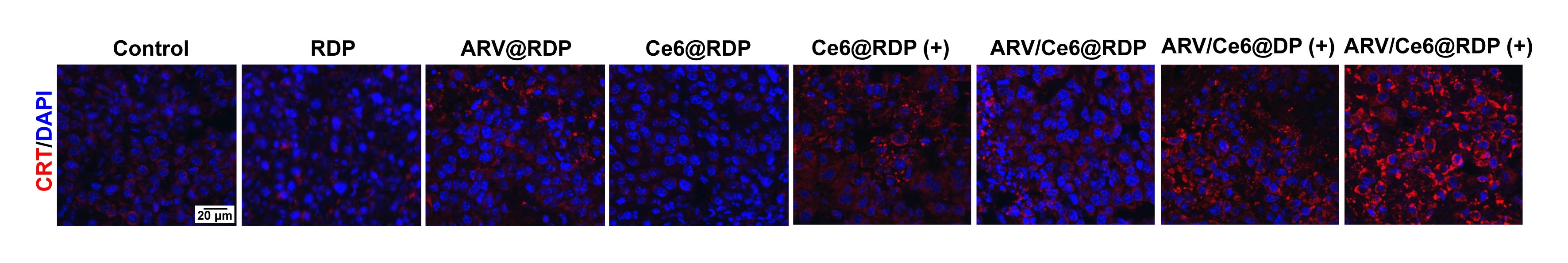


**Figure S40.**

**Representative IF images of CRT expression in tumor tissue from each treatment group**. Scale bar: 20 µm.


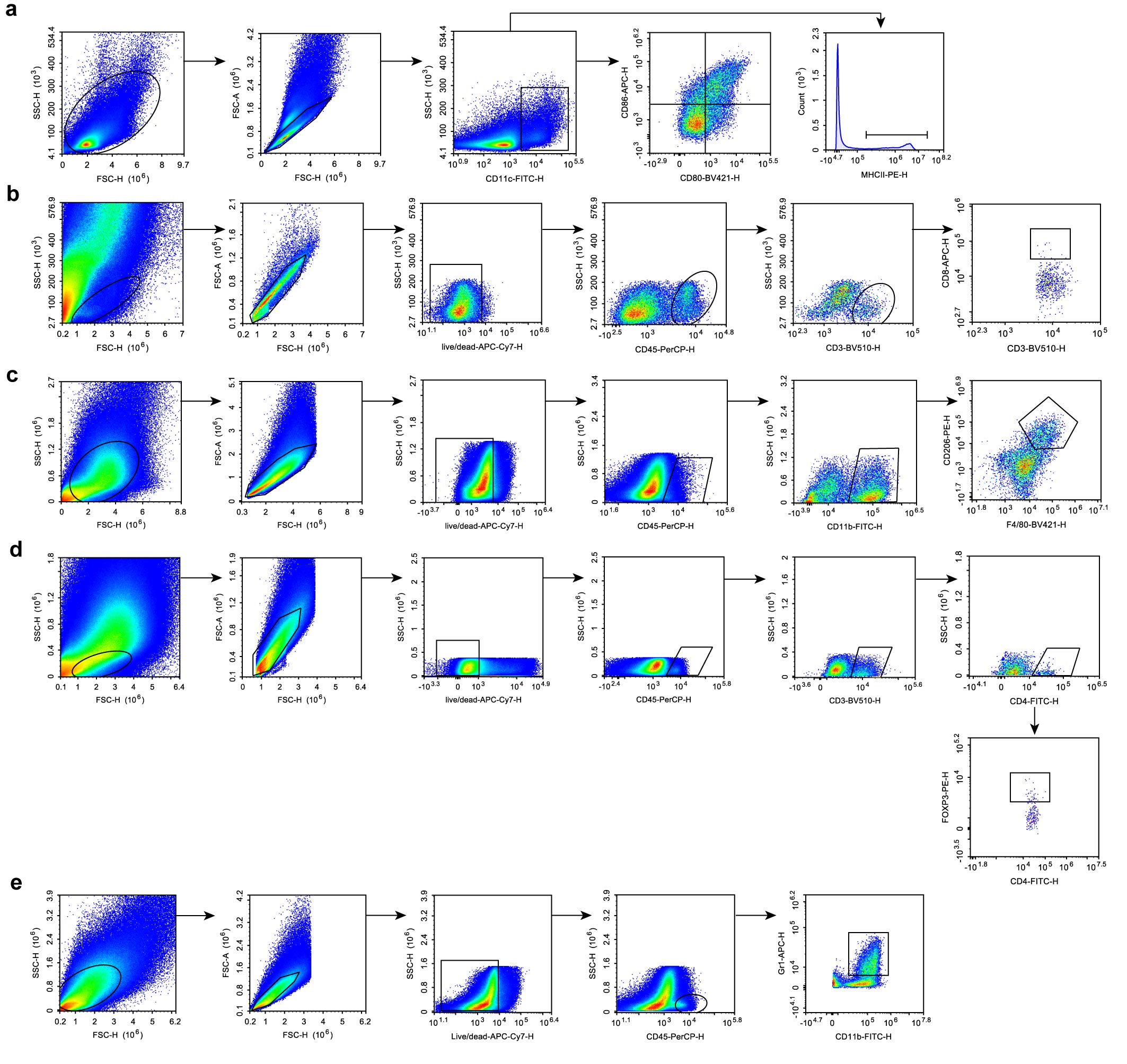


**Figure S41.**

**Flow cytometry gating strategies for Figure 6g and h in B16F10 tumor-bearing mice.** (**a**) Gating strategy for CD11c^+^CD80^+^CD86^+^ DCs and CD11c^+^MHCⅡ^+^ DCs in tumor-draining lymph nodes. (**b**) Gating strategy for L/D^-^CD45^+^CD3^+^CD8^+^ T cells in tumor tissues. (**c**) Gating strategy for L/D^-^CD45^+^CD11b^+^F4/80^+^CD206^+^ macrophages in tumor tissues. (**d**) Gating strategy for L/D^-^CD45^+^CD3^+^CD4^+^Foxp3^+^ Tregs in tumor tissues. (**e**) Gating strategy for L/D^-^CD45^+^CD11b^+^Gr1^+^ MDSCs in tumor tissues.


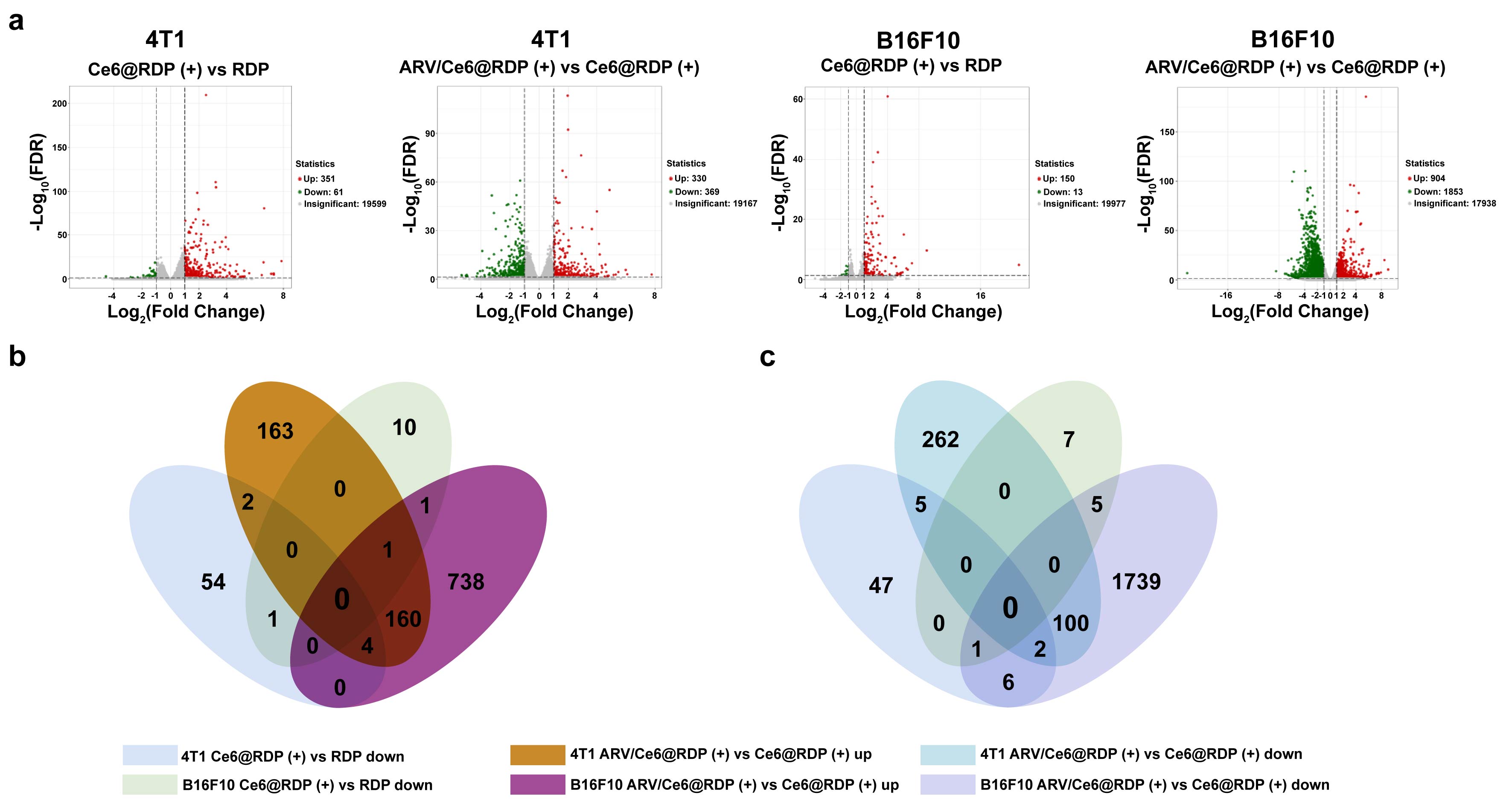


**Figure S42.**

**Volcano plots and Venn diagrams analysis from RNA-seq**. (**a**) Volcano plots of Ce6@RDP (+) vs RDP and ARV/Ce6@RDP (+) vs Ce6@RDP (+) for 4T1 and B16F10 cells from RNA-Seq (Red dots: upregulated genes; Green dots: downregulated genes; Grey dots: insignificant genes; |log2Fold Change| ≥ 1 and FDR < 0.05). (**b**) Venn diagram displaying the intersection of downregulated genes in Ce6@RDP (+) vs RDP and upregulated genes in ARV/Ce6@RDP (+) vs Ce6@RDP (+) for 4T1 and B16F10 cells. (**c**) Venn diagrams showing the overlaps of downregulated genes in Ce6@RDP (+) vs RDP and downregulated genes in ARV/Ce6@RDP (+) vs Ce6@RDP (+) for 4T1 and B16F10 cells.


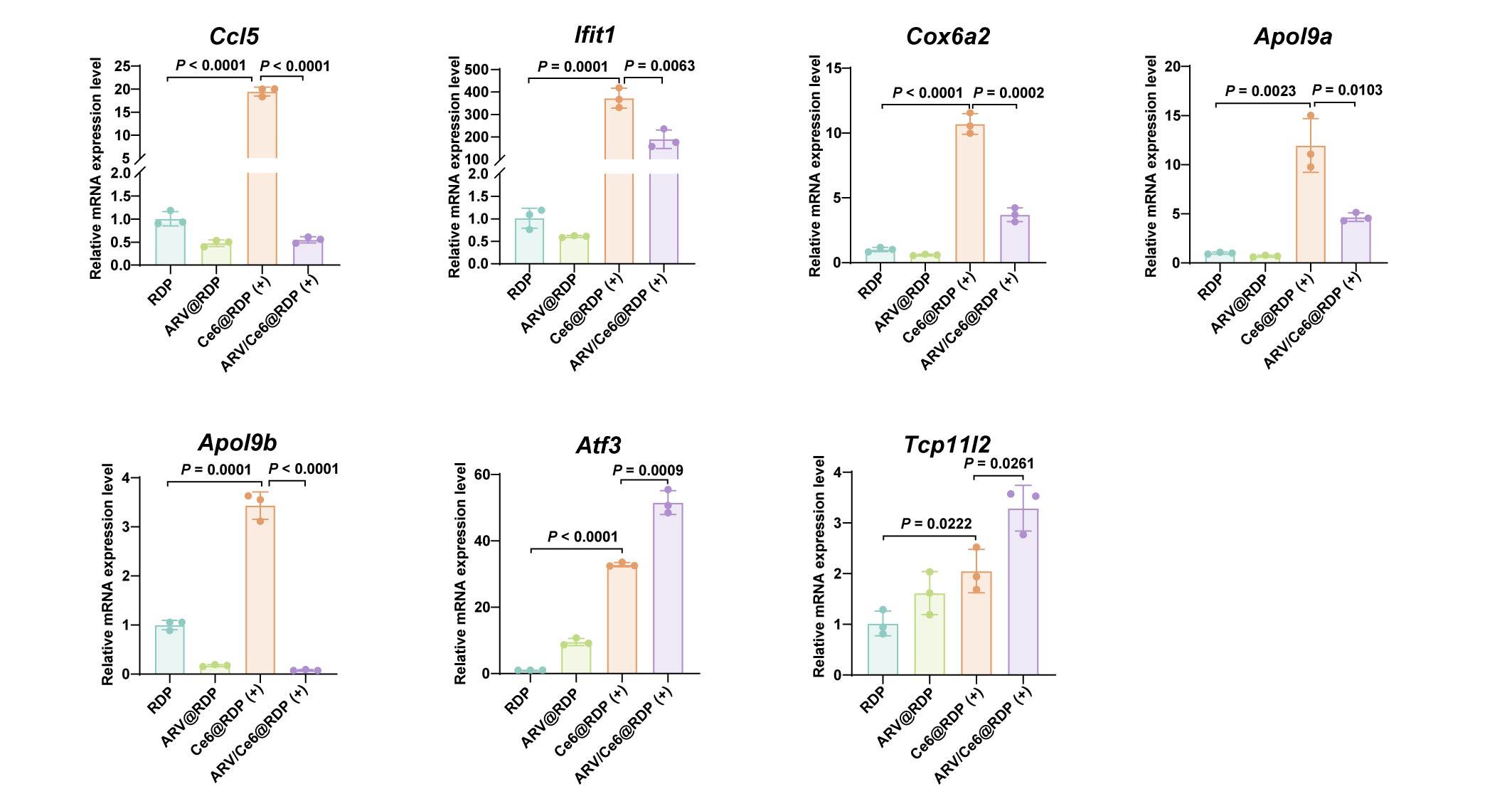


**Figure S43.**

**qPCR analysis of *Ccl5*, *Ifit1*, *Cox6a2*, *Apol9a*, *Apol9b*, *Atf3* and *Tcp11l2* genes expression in 4T1 cells receiving different treatments** (n = 3 per group, two-tailed unpaired Student’s *t* test). Data are shown as mean ± SDs.


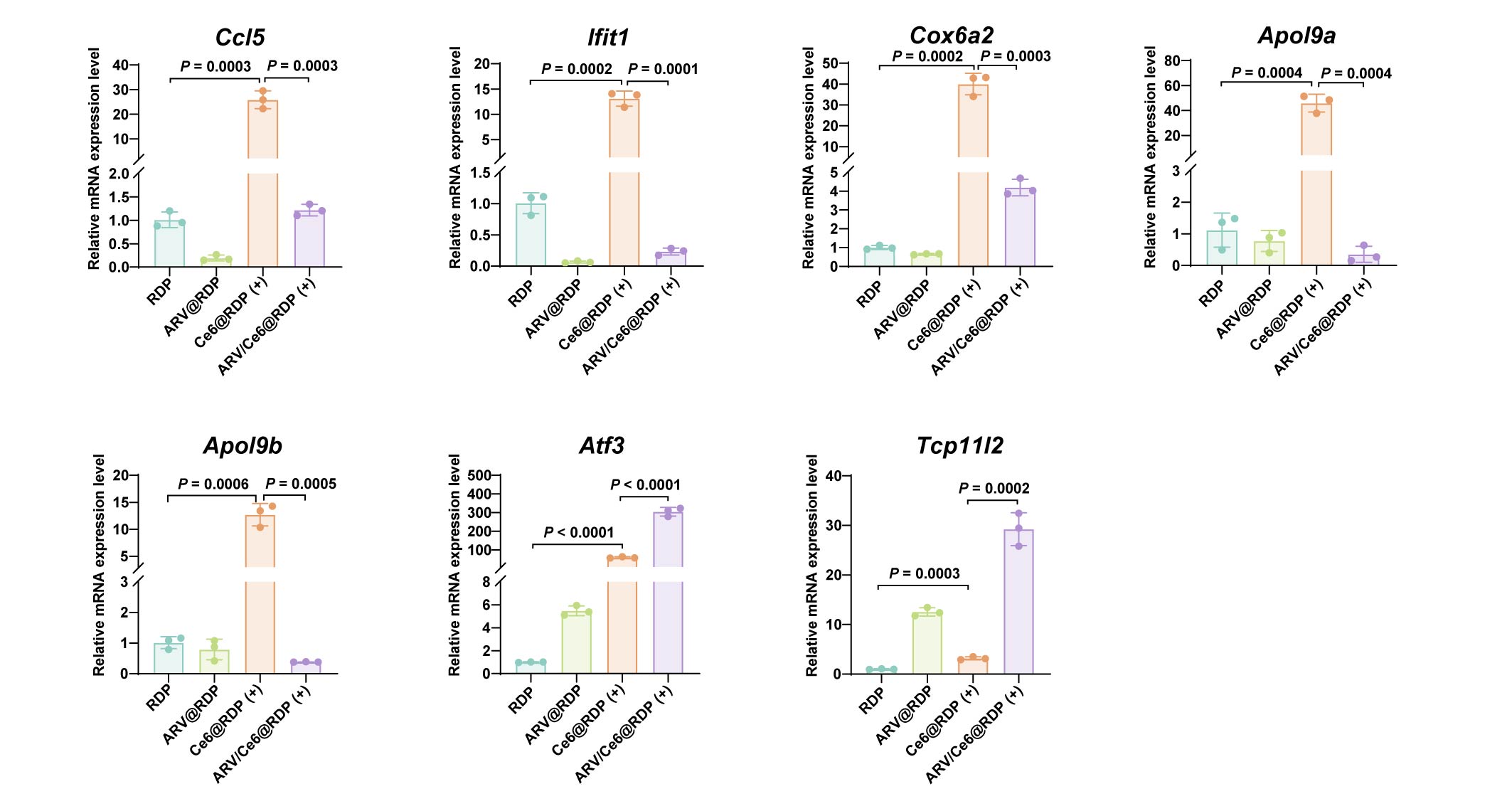


**Figure S44.**

**Changes in genes (*Ccl5*, *Ifit1*, *Cox6a2*, *Apol9a*, *Apol9b*, *Atf3* and *Tcp11l2*) expression of B16F10 cells following various treatments** (n = 3 per group, two-tailed unpaired Student’s *t* test). Data are shown as mean ± SDs.


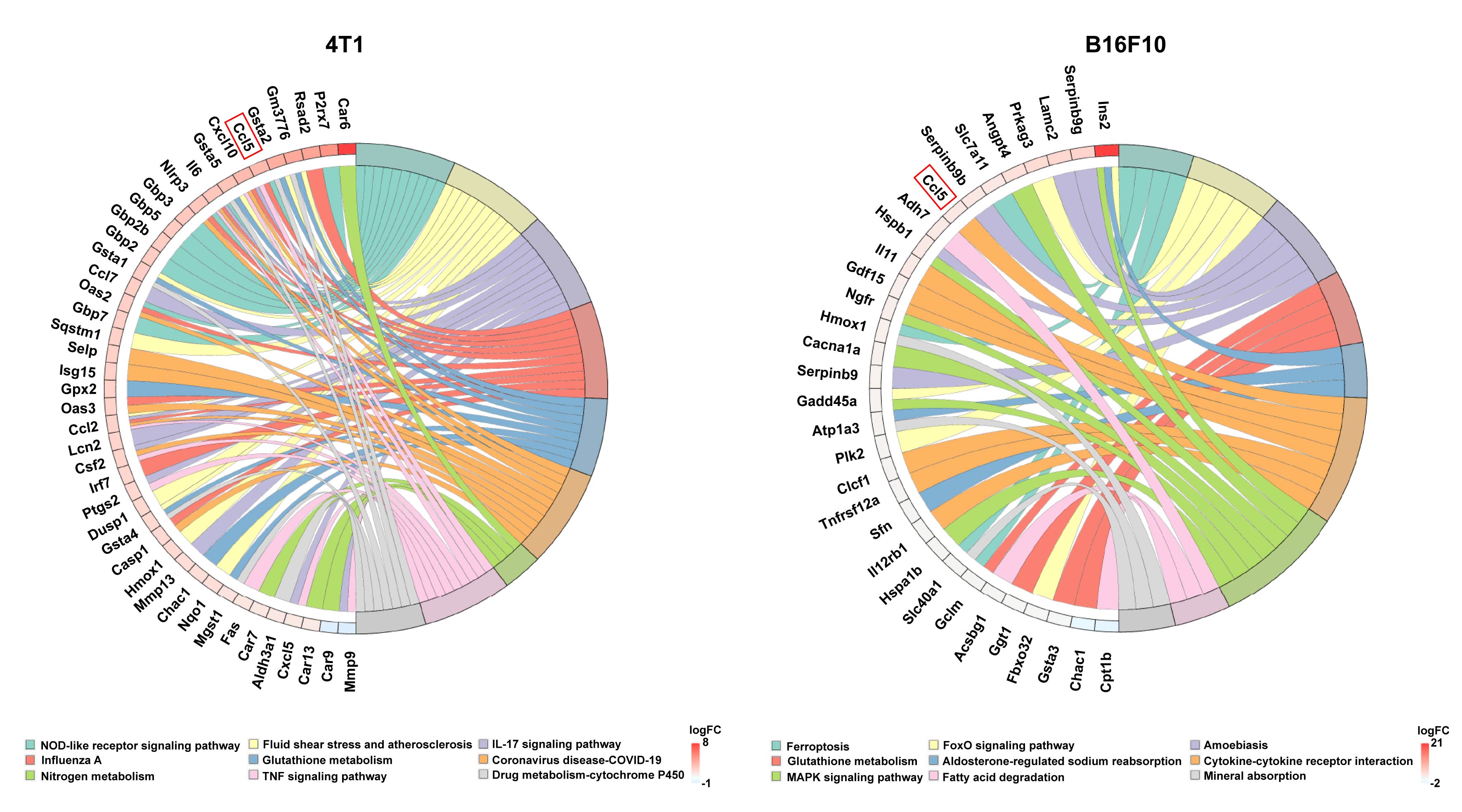


**Figure S45.**

**Chord diagrams of KEGG pathway enrichment analysis between Ce6@RDP (+) and RDP groups in 4T1 and B16F10 cells**. On the left side of each chord plot are the top 10 genes with the largest |logFC| in each category, and on the right side are the 9 pathways with the most significant enrichment.


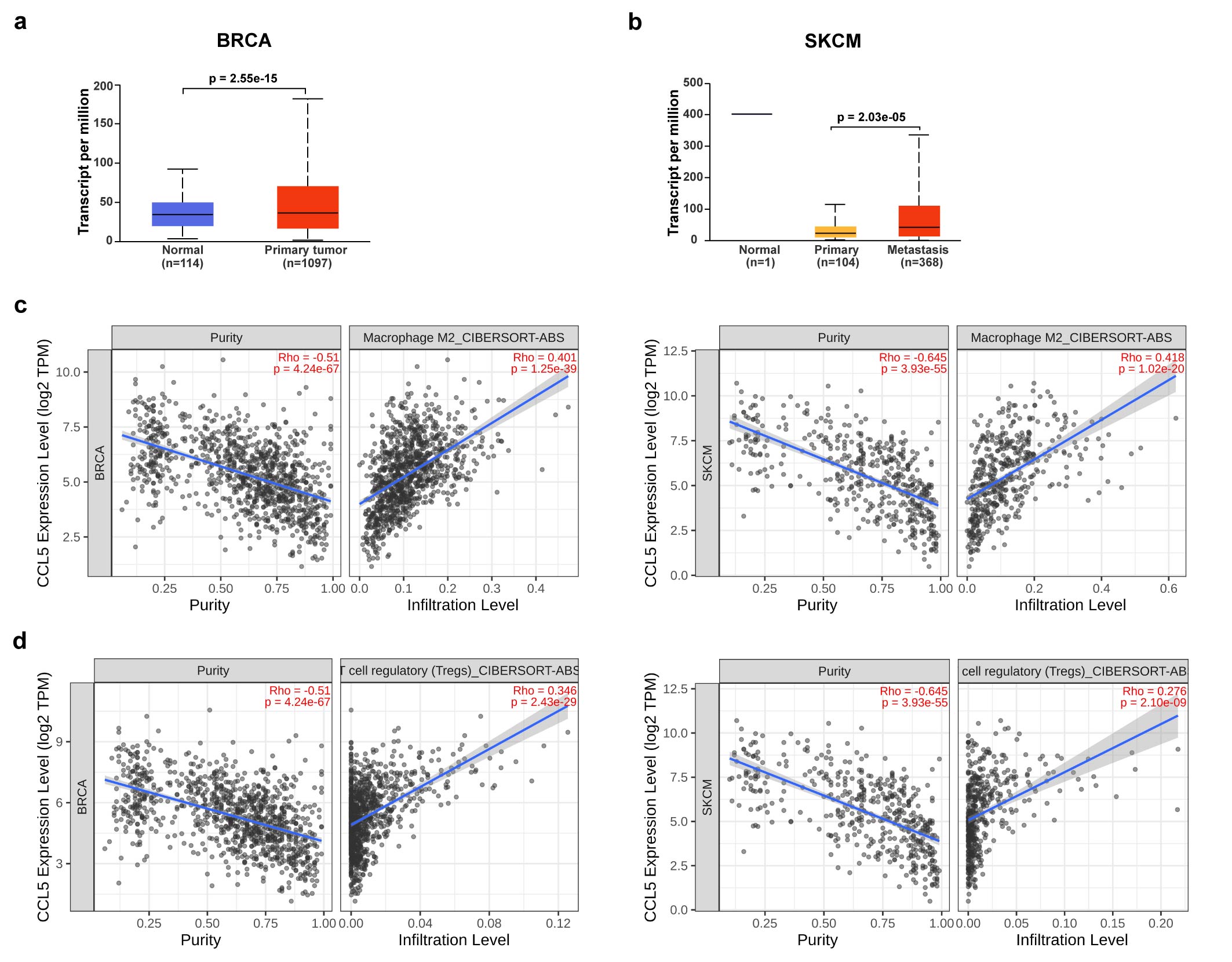


**Figure S46.**

**Online database analysis of mRNA expression, patient survival and immune infiltration related to *Ccl5* gene in BRCA and SKCM**. (**a**) mRNA expression levels of *Ccl5* in BRCA samples and normal tissues from the UALCAN database. (**b**) mRNA expression levels of *Ccl5* in primary SKCM samples, metastasis SKCM samples and normal tissues from the UALCAN database. (**c, d**) Correlation analysis of *Ccl5* expression and M2 macrophages (**c**) or Tregs cells (**d**) immune infiltration in BRCA and SKCM through TIMER 2.0 database.


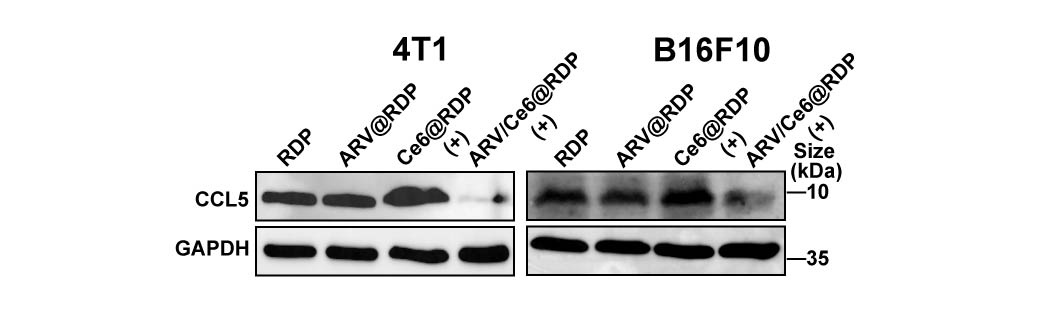


**Figure S47.**

**Changes in CCL5 protein expression in 4T1 and B16F10 cells** **after various treatment**s.


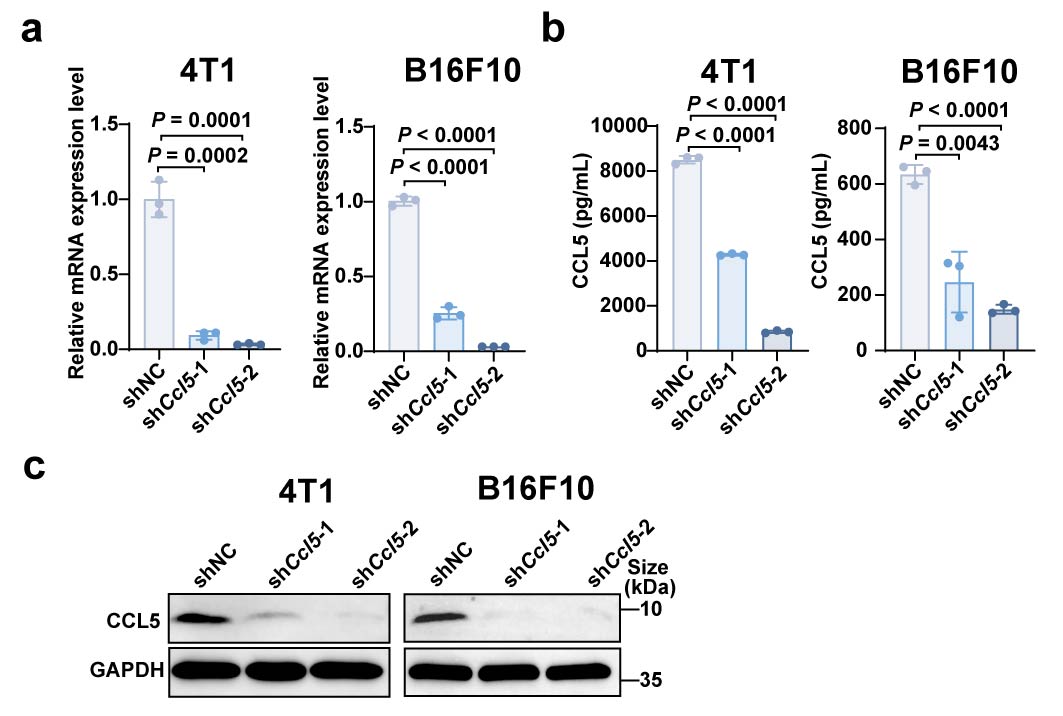


**Figure S48.**

**Validation of *Ccl5* knockdown in 4T1 and B16F10 cells**. (**a**) qPCR analysis of *Ccl5* mRNA expression (n = 3 per group, two-tailed unpaired Student’s *t* test). (**b**) ELISA analysis of CCL5 protein secretion (n = 3 per group, two-tailed unpaired Student’s *t* test). (**c**) Western blot analysis of CCL5 protein expression. All data in this figure are shown as mean ± SDs.

**
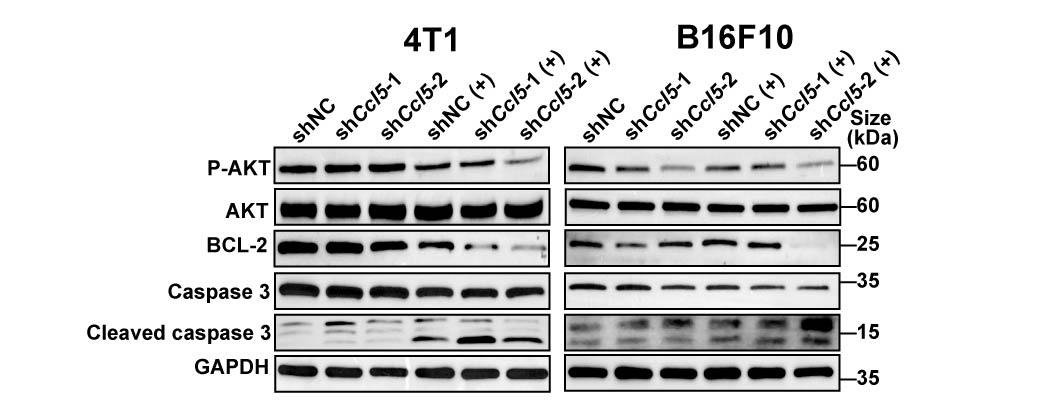
**

**Figure S49.**

**Changes in proliferation and apoptosis associated proteins of *Ccl5*-knockdown 4T1 and B16F10 cells after PDT treatment**.


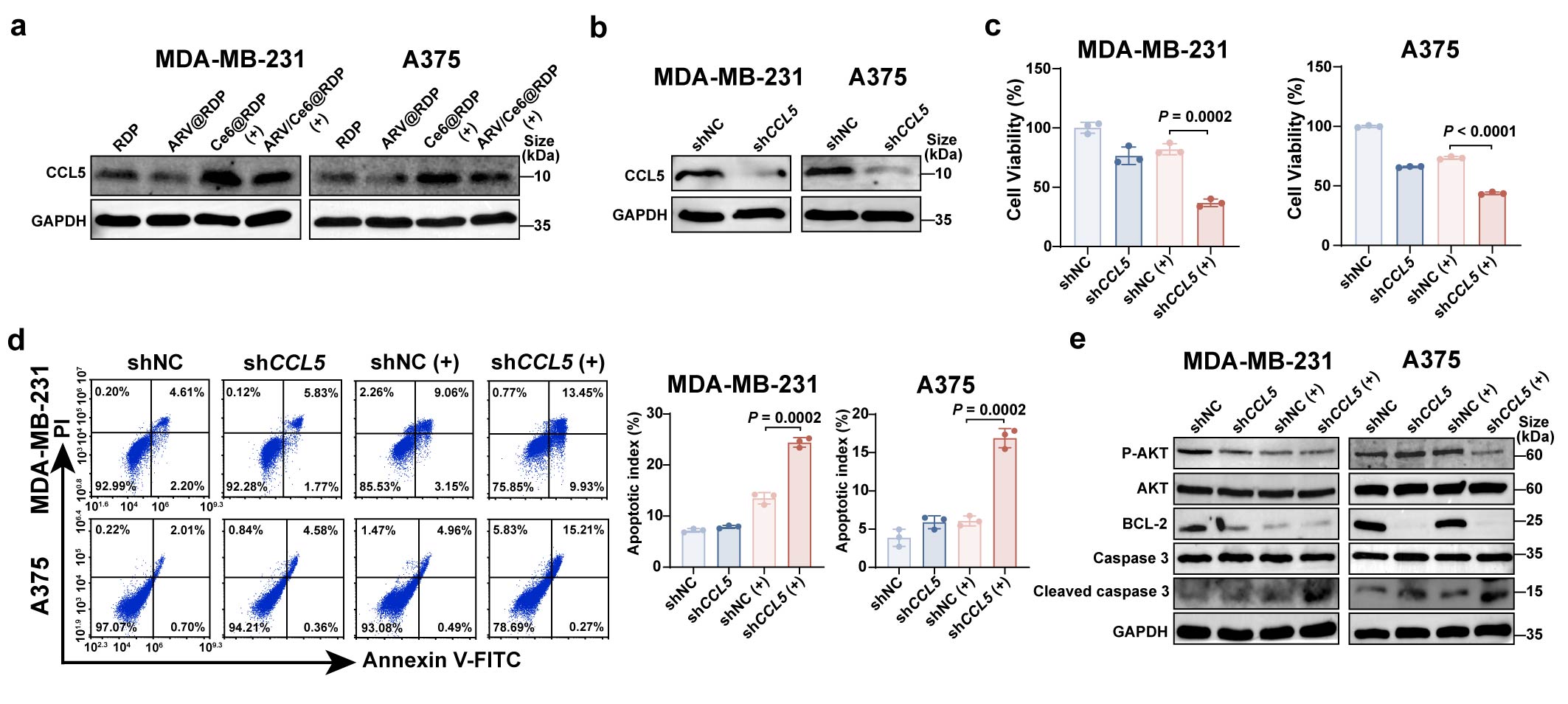


**Figure S50.**

**Effect of *CCL5* knockdown on proliferation and apoptosis of human tumor cells treated with PDT**. (**a**) Changes in CCL5 protein expression of MDA-MB-231 and A375 cells following various treatments. (**b**) Validation of *CCL5* knockdown in MDA-MB-231 and A375 cells detected by western blot. (**c-e**) Cell viability assay (**c**, n = 3 per group, two-tailed unpaired Student’s *t* test), apoptosis analysis (**d**, n = 3 per group, two-tailed unpaired Student’s *t* test) and related proteins expression changes (**e**) for *CCL5*-knockdown MDA-MB-231 and A375 cells and normal control cells, with and without PDT. All data in this figure are shown as mean ± SDs.


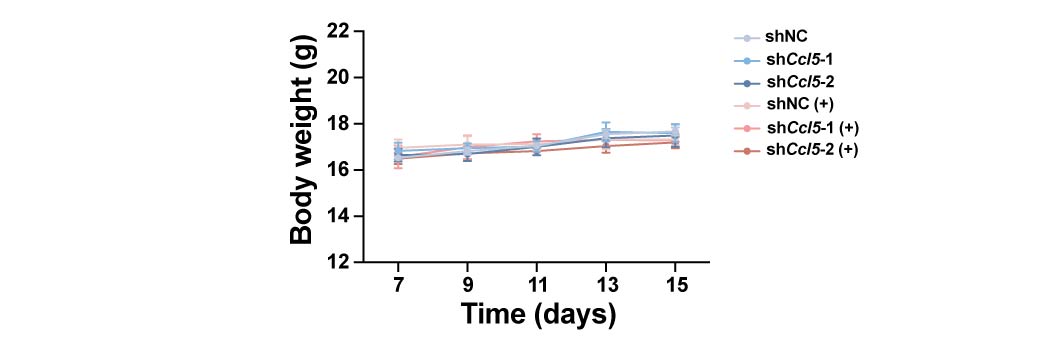


**Figure S51.**

**Body weight fluctuation in *Ccl5*-knockdown 4T1 tumor-bearing mice and control tumor-bearing mice after varied therapy** (n = 6 per group). Data are shown as mean ± SEMs.


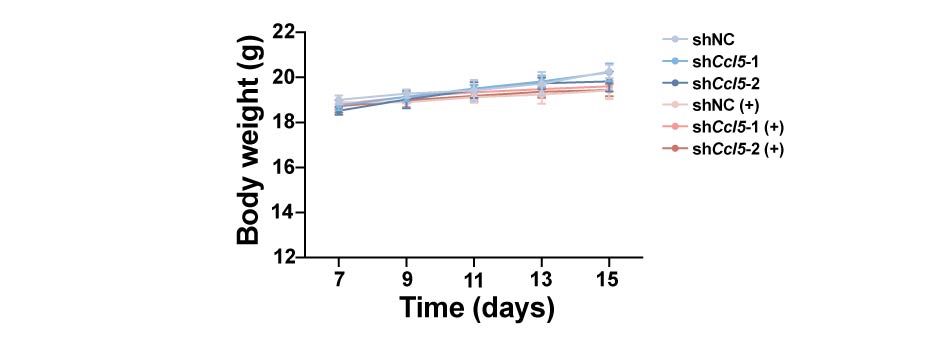


**Figure S52.**

**Body weight curve of *Ccl5*-knockdown B16F10 tumor-bearing mice and control tumor-bearing mice throughout treatments with PDT and without PDT**. (n = 5 per group). Data are shown as mean ± SEMs.


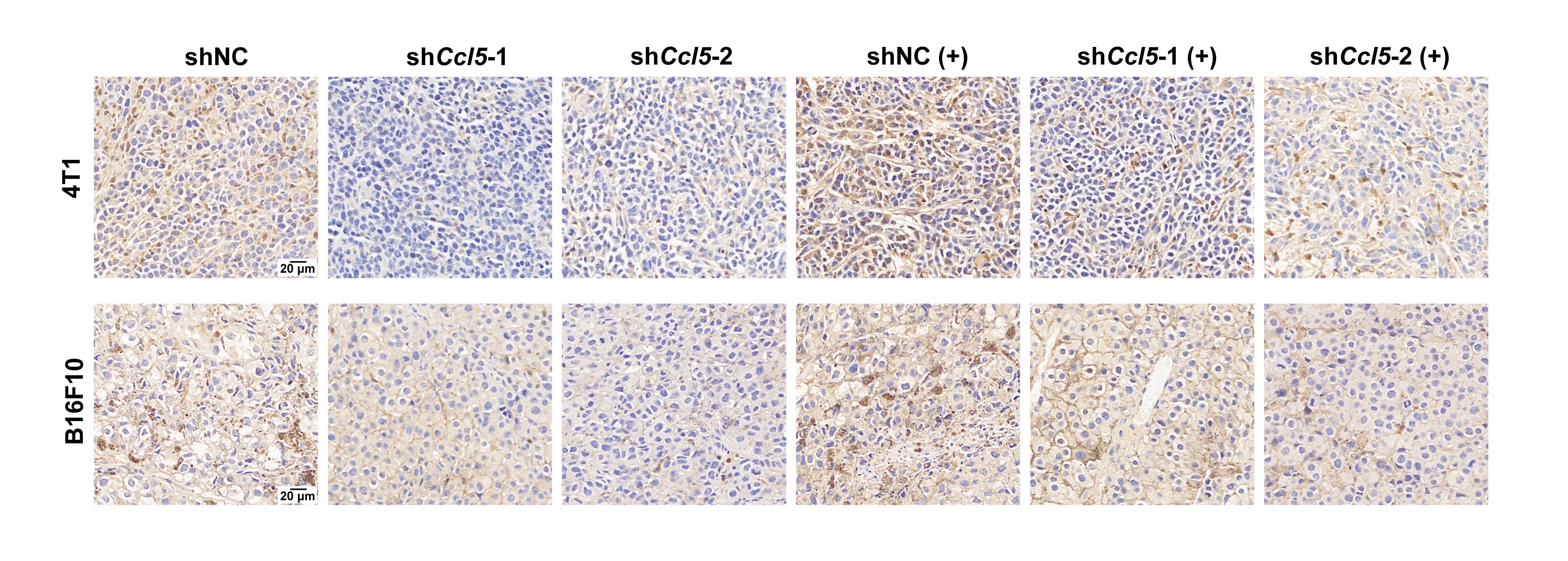


**Figure S53.**

**Representative IHC images of CCL5 protein in tumor tissue from various groups**. Scale bar: 20 µm.


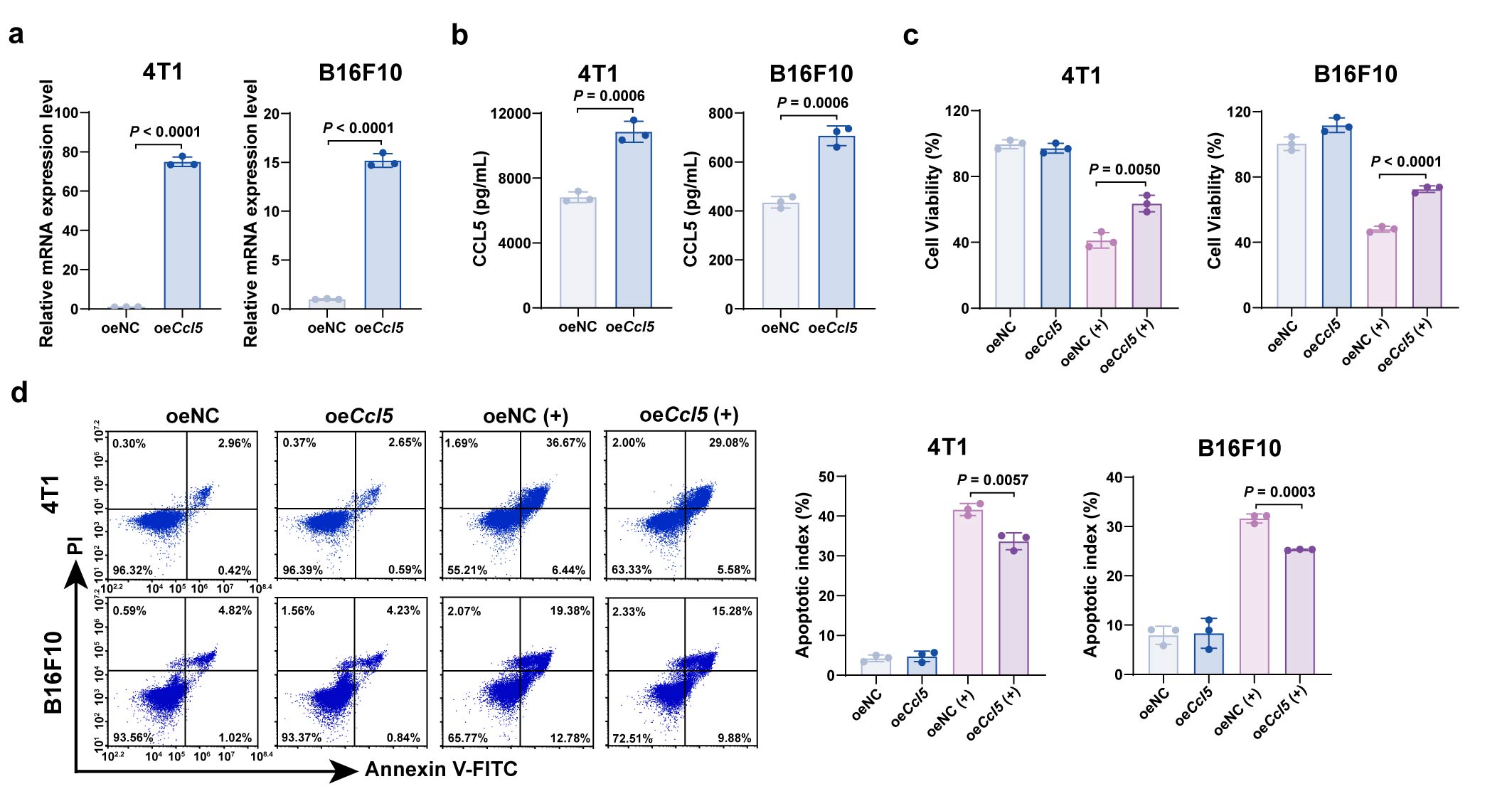


**Figure S54.**

**Effect of *Ccl5* overexpression on proliferation and apoptosis of PDT treated 4T1 and B16F10 cells**. (**a, b**) *Ccl5* mRNA expression and CCL5 protein secretion analyses for constructed *Ccl5*-overexpression tumor cells by RT-qPCR detection (**a**, n = 3 per group, two-tailed unpaired Student’s *t* test) and ELISA assay (**b**, n = 3 per group, two-tailed unpaired Student’s *t* test), respectively. (**c**) Cell viability assay showing the reduced proliferation inhibition of *Ccl5*-overexpression 4T1 and B16F10 cells after PDT (n = 3 per group, two-tailed unpaired Student’s *t* test). (**d**) Flow cytometric analysis of apoptosis for *Ccl5*-overexpression 4T1 and B16F10 cells and normal control cells, with and without PDT (n = 3 per group, two-tailed unpaired Student’s *t* test). All data in this figure are shown as mean ± SDs.


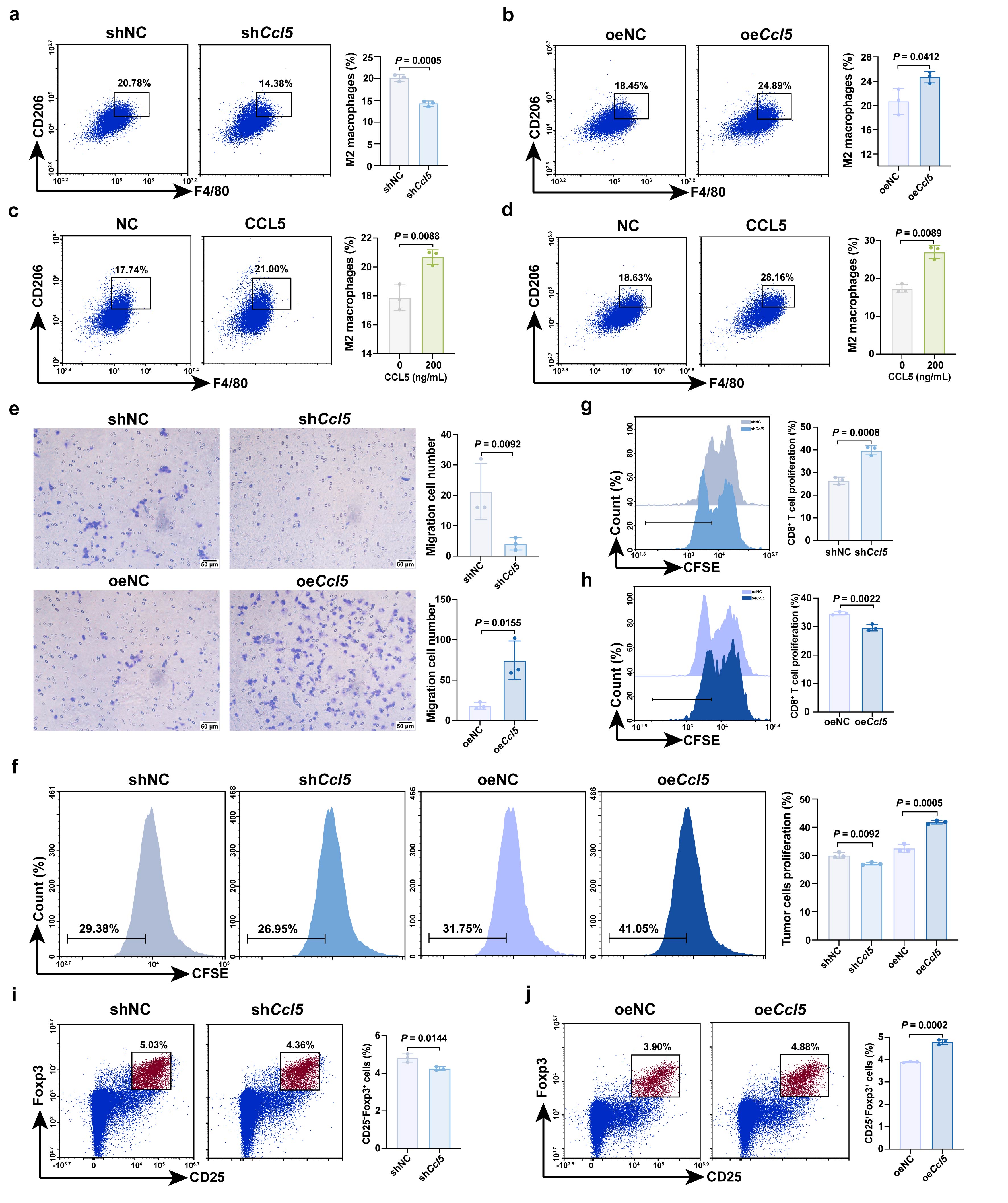


**Figure S55.**

**CCL5 mediated immune regulation.** (**a, b**) Flow cytometric analyses for M2 polarization of BMDMs after treatment with culture medium from *Ccl5*-knockdown (**a**) or *Ccl5*-overexpression (**b**) B16F10 cells and control cells (n = 3 per group, two-tailed unpaired Student’s *t* test). (**c, d**) Flow cytometric analyses for M2 polarization of BMDMs from BALB/c (**c**) and C57BL/6 (**d**) mice after CCL5 treatment (n = 3 per group, two-tailed unpaired Student’s *t* test). (**e**) Recruitment of M2 macrophages by *Ccl5*-knockdown or *Ccl5*-overexpression B16F10 cells (n = 3 per group, two-tailed unpaired Student’s *t* test, scale bar: 50 µm). (**f**) CFSE staining analyses for B16F10 cells proliferation by BMDMs receiving different treatments (n = 3 per group, two-tailed unpaired Student’s *t* test). (**g, h**) CFSE staining analyses for CD8^+^ T cells proliferation by BMDMs treated with different culture medium from *Ccl5*-knockdown (**g**) or *Ccl5*-overexpression (**h**) B16F10 cells (n = 3 per group, two-tailed unpaired Student’s *t* test). (**i, j**) Flow cytometric analyses of Tregs ratio changes caused by *Ccl5*-knockdown (**i**) or *Ccl5*-overexpression (**j**) B16F10 cells (n = 3 per group, two-tailed unpaired Student’s *t* test). All data in this figure are shown as mean ± SDs.


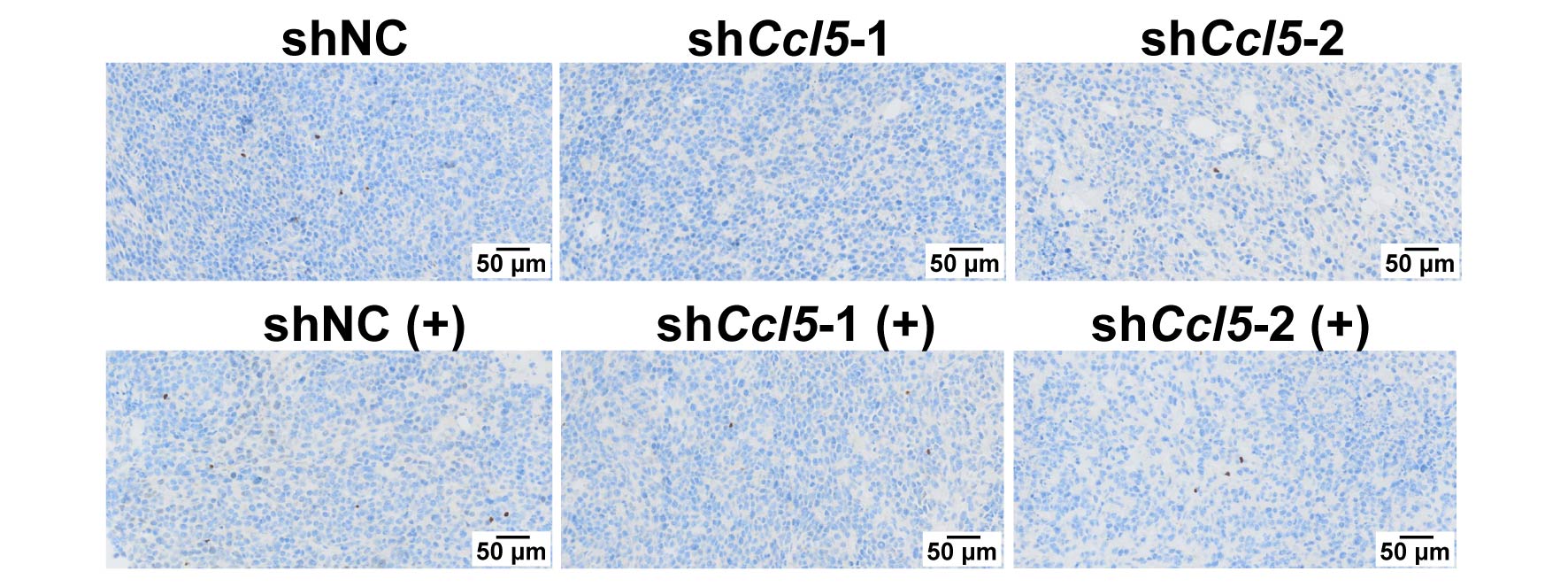


**Figure S56.**

**Representative pictures of Foxp3 expression of 4T1 tumor sections from different treatment groups**. Scale bar: 50 µm.


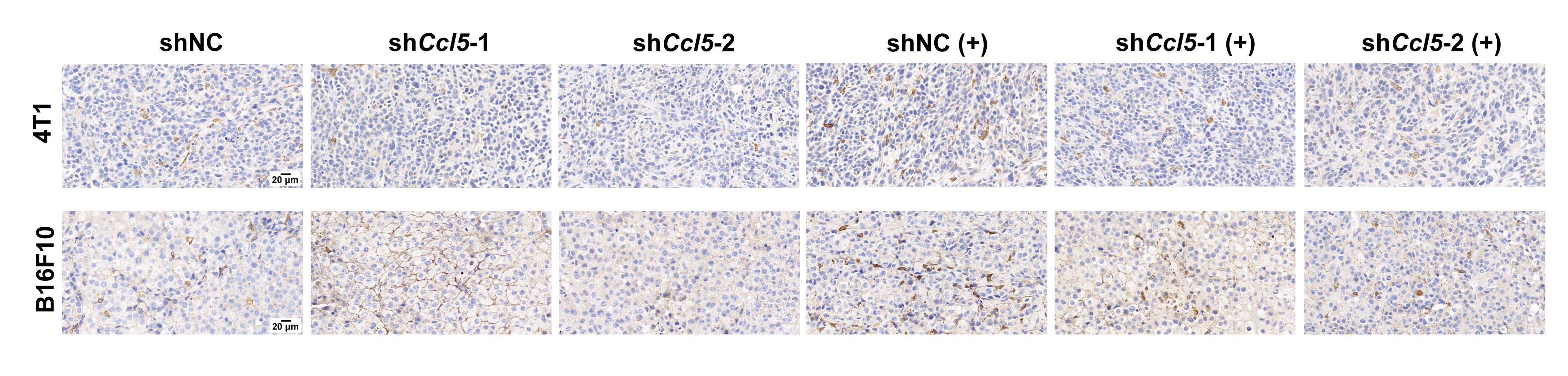


**Figure S57.**

**Representative CD206 IHC images in tumor tissue from various treatment groups**. Scale bar: 20 µm.


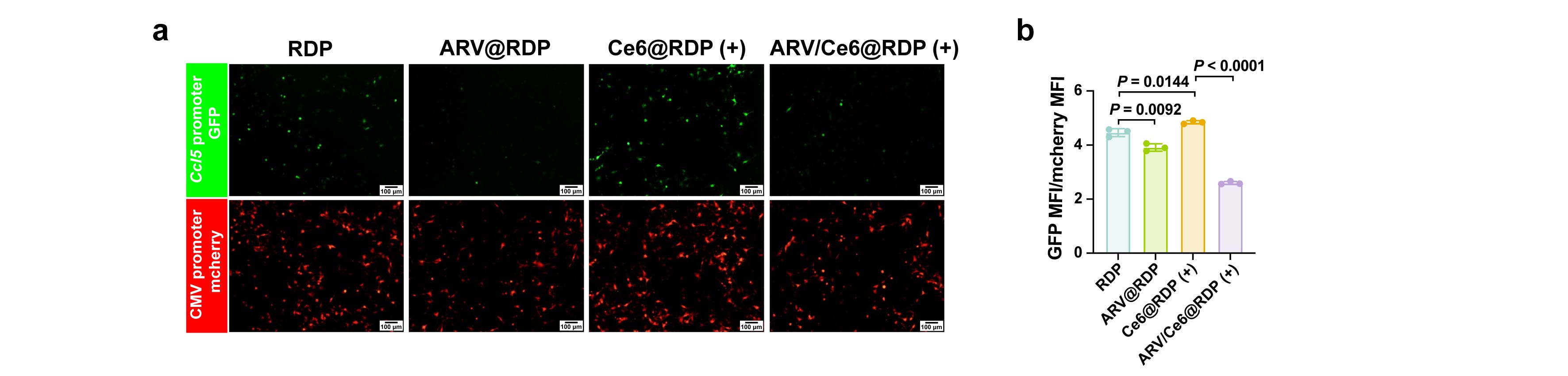


**Figure S58.**

**Dual-fluorescence plasmid system analysis of the transcriptional activity of the *Ccl5* promoter.** (**a**) Representative fluorescent images of B16F10 cells co-transfected with PVAX-mcherry (Red fluorescence) and *Ccl5* promoter-GFP (Green fluorescence) plasmids after various treatments (Scale bar: 100 µm). (**b**) Flow cytometric quantitative analyses for the mean fluorescence intensity ratio of GFP and mcherry in cells from (**a**) (n = 3 per group, two-tailed unpaired Student’s *t* test). Data are shown as mean ± SDs.

**Table S1.** List of antibodies used for Western blotting

| **Name** | **Catalog#** | **Dilution** | **Company** |
| --- | --- | --- | --- |
| BRD4 | ab128874 | 1/1000 | Abcam |
| c-Myc | ab185656 | 1/2000 | Abcam |
| Phospho-Akt | 4060 | 1/1000 | CST |
| Akt | 4691 | 1/1000 | CST |
| Phospho-p44/42 MAPK (Erk1/2) | 4370 | 1/1000 | CST |
| p44/42 MAPK (Erk1/2) | 4695 | 1/1000 | CST |
| Phospho-Stat3 | 9145 | 1/1000 | CST |
| Stat3 | 12640 | 1/1000 | CST |
| Bcl2 | 2870 | 1/1000 | CST |
| Caspase 9 | 9508 | 1/1000 | CST |
| Caspase 3 | 9662 | 1/1000 | CST |
| Cleaved caspase 3 | 9661 | 1/1000 | CST |
| PARP | 9542 | 1/1000 | CST |
| Cyclin D1 | 55506 | 1/1000 | CST |
| CDK4 | ab199728 | 1/2000 | Abcam |
| Cyclin B1 | sc-245 | 1/500 | Santa cruz |
| CDC2 | sc-54 | 1/500 | Santa cruz |
| PDL1 | ab213480 | 1/1000 | Abcam |
| CD47 | CY5251 | 1/1000 | Abways |
| RANTES | sc-365826 | 1/500 | Santa cruz |
| GAPDH | 60004-1-Ig | 1/50000 | Proteintech |
| Goat anti-mouse IgG-HRP | AS003 | 1/10000 | ABclonal |
| Goat anti-rabbit IgG-HRP | AS014 | 1/10000 | ABclonal |

**Table S2.** List of primers used for qPCR

| **Name** | **Forward Sequence (5'-3')** | **Reverse Sequence (5'-3')** |
| --- | --- | --- |
| m-*Itgav* | CCGTGGACTTCTTCGAGCC | CTGTTGAATCAAACTCAATGGGC |
| h-*ITGAV* | GCTGTCGGAGATTTCAATGGT | TCTGCTCGCCAGTAAAATTGT |
| m-*Itgb3* | GGCGTTGTTGTTGGAGAGTC | CTTCAGGTTACATCGGGGTGA |
| h-*ITGB3* | AGTAACCTGCGGATTGGCTTC | GTCACCTGGTCAGTTAGCGT |
| m-*Cd80* | TCATCCTGGGCCTGGTCCTTTC | TGGTGTGGTTGCGAGTCGTATTG |
| m-*Cd86* | CAGAACTTACGGAAGCACCA | ATAAGCTTGCGTCTCCACGG |
| m-*H2Ab1* | TGGCCTTTTCATCCGTCACA | TGGCAGTCAGGAATTCGGAG |
| m-*Tnf* | ATGGCCTCCCTCTCATCAGT | TTTGCTACGACGTGGGCTAC |
| m-*Mrc1* | AAGGCTATCCTGGTGGAAGAA | AGGGAAGGGTCAGTCTGTGTT |
| m-*Arg1* | CCACAGTCTGGCAGTTGGAAG | GGTTGTCAGGGGAGTGTTGATG |
| m-*Ym1* | AGGGCCCTTATTGAGAGGAG | GCACTGTGGAAAAACCGTTGA |
| m-*Irf4* | AATGGGAAACTCCGACAGTG | TAGGAGGATCTGGCTTGTCG |
| m-*Ccl5* | GCTGCTTTGCCTACCTCTCC | TCGAGTGACAAACACGACTGC |
| m-*Ifit1* | CTGAGATGTCACTTCACATGGAA | GTGCATCCCCAATGGGTTCT |
| m-*COX6a2* | CTGCTCCCTTAACTGCTGGAT | GATTGTGGAAAAGCGTGTGGT |
| m-*Apol9a* | CTTCAGTGCTGCCTCTGACA | GGACCTTGCTGGCAATCCTA |
| m-*Apol9b* | GTAAGTTGAGCCTCTTCGCT | CTTGGATACAAACGCCTGCC |
| m-*Atf3* | GAGGATTTTGCTAACCTGACACC | TTGACGGTAACTGACTCCAGC |
| m-*Tcp11I2* | CCTTTCAACGGTGAGAAGCAA | GCCTGGAACACTCATAGTCACT |
| m-*Gapdh* | TGGCCTTCCGTGTTCCTAC | GAGTTGCTGTTGAAGTCGCA |
| h-*GAPDH* | GGAGCGAGATCCCTCCAAAAT | GGCTGTTGTCATACTTCTCATGG |
| m-*β-actin* | GGCTGTATTCCCCTCCATCG | CCAGTTGGTAACAATGCCATGT |

**Table S3.** List of flow antibodies used for flow cytometry assays

| **Name** | **Fluorophore** | **Catalog#** | **Company** |
| --- | --- | --- | --- |
| Anti-PDL1 | PE | 124308 | Biolegend |
| Anti-CD11c | PE | 557401 | BD |
| Anti-CD11c | FITC | 117306 | Biolegend |
| Anti-CD80 | APC | 560016 | BD |
| anti-CD80 | BV421 | 100712 | Biolegend |
| Anti-CD86 | PE-Cy7 | 560582 | BD |
| Anti-CD86 | APC | 105012 | Biolegend |
| Anti-MHCII | FITC | 565254 | BD |
| Anti-MHCII | PE | 107608 | Biolegend |
| Anti-CD3 | APC | 100236 | Biolegend |
| Anti-CD3 | FITC | 100204 | Biolegend |
| Anti-CD3 | PE | 100206 | Biolegend |
| Anti-CD3 | PE-Cy7 | 100220 | Biolegend |
| Anti-CD3 | BV510 | 100234 | Biolegend |
| Anti-CD8a | PE-Cy7 | 100722 | Biolegend |
| Anti-CD8a | APC | 100712 | Biolegend |
| Anti-CD4 | FITC | 100406 | Biolegend |
| anti-IFN-r | PE | 505808 | Biolegend |
| Anti-CD11b | PE | 101208 | Biolegend |
| Anti-CD11b | FITC | 101206 | Biolegend |
| Anti-F4/80 | APC | 123116 | Biolegend |
| Anti-F4/80 | BV421 | 123137 | Biolegend |
| Anti-F4/80 | PE | 565410 | BD |
| Anti-CD206 | PE | 141706 | Biolegend |
| Anti-CD206 | Alexa Fluor® 647 | 565250 | BD |
| Anti-CD45 | PerCP | 103130 | Biolegend |
| Anti-Foxp3 | PE | 126404 | Biolegend |
| Anti-CD49b | FITC | 553857 | BD |
| Anti-CD107a | PE | 558661 | BD |
| Anti-Gr1 | APC | 553129 | BD |

**Table S4.** List of primers used for constructing *Ccl5* gene knockdown and overexpression plasmids

| **Mouse sh*Ccl5*-1** | |
| --- | --- |
| Forward oligo  (5'-3') | CCGGCCAGAGAAGAAGTGGGTTCAACTCGAGTTGAAC  CCACTTCTTCT TGGTTTTTG |
| Reverse oligo  (5'-3') | AATTCAAAAACCAGAGAAGAAGTGGGTTCAACTCGAG  TTGAACCCACTTCTTCTCTGG |
| **Mouse sh*Ccl*5-2** | |
| Forward oligo  (5'-3') | CCGGCGTGTTTGTCACTCGAAGGAACTCGAGTTCCTTC  GAGTGACAAACACGTTTTTG |
| Reverse oligo  (5'-3') | AATTCAAAAACGTGTTTGTCACTCGAAGGAACTCGAGT  TCCTTCGAGTGACAAACACG |
| **Human sh*CCL5*** | |
| Forward oligo  (5'-3') | CCGGGTATTTCTACACCAGTGGCAACTCGAGTTGCCAC  TGGTGTAGAAATACTTTTTG |
| Reverse oligo  (5'-3') | AATTCAAAAAGTATTTCTACACCAGTGGCAACTCGAGT  TGCCACTGGTGTAGAAATAC |
| **Mouse oe*Ccl5*** | |
| Forward oligo  (5'-3') | GATGACAAGTCTAGAGAATTCATGAAGATCTCTGCAGC  TGCCC |
| Reverse oligo  (5'-3') | GTAAAGCTTCCATGGCTCGAGCTAGCTCATCTCCAAATA  GTTGATGTATT |

**Table S5.** List of primers used for Dual-luciferase reporter assay

| ***Ccl5* Promoter (-1953 - +57 bp)** | |
| --- | --- |
| Forward primer  (5'-3') | GCGTGCTAGCCCGGGCTCGAGTACATTTATCCA  CTAACGTCATGGTG |
| Reverse primer  (5'-3') | CAGTACCGGAATGCCAAGCTTAGAGATCTTCAT  GGTACCCGCG |
| ***Ccl5* Promoter (-979 - +8 bp)** | |
| Forward primer  (5'-3') | GCGTGCTAGCCCGGGCTCGAGCCTGTGCCCAC  CATTCACACACTGATT |
| Reverse primer  (5'-3') | CAGTACCGGAATGCCAAGCTTTCTGCAAGGGG  TGCTCTGC |
| ***Ccl5* Promoter (-150 - +65 bp)** | |
| Forward primer  (5'-3') | GCGTGCTAGCCCGGGCTCGAGTTTTCTTTTCCA  TTTTGTGTTTTCA |
| Reverse primer  (5'-3') | CAGTACCGGAATGCCAAGCTTGATCTTCATGGT  ACCCGCGG |

**Table S6.** List of primers used for ChIP-PCR

| **Name** | **Forward Sequence (5'-3')** | **Reverse Sequence (5'-3')** |
| --- | --- | --- |
| Primer 1 | GTCCAGCTGAGATGCACTGT | AGTTCTGAAGGGGTGGGAGT |
| Primer 2 | ACGTCATGGTGATGTCATGGT | GTGCATCTCAGCTGGACACT |
| Primer 3 | CTTGAGATCAGACTGTGCCCA | TGTGCAATGGAGTCTCCCAG |
| Primer 4 | AAGTTGTGCCTTTCCCTGGG | ATGGTTGGTGCCAGACAGAG |
| Primer 5 | ACAGGACAGACTTGGGACCA | TATGGGGTCCAGGTGAGAGT |
| Primer 6 | ACTTGGGACCATATGAGCACA | CTCTTTATGGGGTCCAGGTGAG |
| Primer 7 | CTCCACCAGCCTGTCATCTG | TGCCACAGTGGGAATGGATC |
| Primer 8 | GATCCATTCCCACTGTGGCA | TGGGAGGCTGGGTCAAGATA |
| Primer 9 | CCCTCCACTGCATCCATGTT | AGGTGCCTAGTCCAGAGTCA |
| Primer 10 | GCTGGGACTAGACTGTGACG | GCATTCAATCAGTCTCCGCC |
| Primer 11 | TGTACACACACACATGCA | AGAATCTCTGTGGAAATAAAGA |
| Primer 12 | AGCCCTATCAGATCCCTTGACT | TGGGAACAAAAGAGTTGTGTCC |
| Primer 13 | TCTCAGATCACATGTCACACACT | CCTGATTCCCAGATAACTAGGT |
| Primer 14 | ACCTGGCAAATTCCTTACAACA | AACAGACCACACTTGTGTCA |
| Primer 15 | AGTCTGGGCTACAACTTGGG | GTCAGTTAAGGTCAAGCCATTGG |
| Primer 16 | TTGGCCAGAGAGGGAGTCAT | ACCCTTGGAAGTATGACTCTGC |
| Primer 17 | TGACAGCAACAAGTGTTTGGT | GAGCCAGGGTAGCAGAGGAA |
| Primer 18 | GCTACCCTGGCTCCCTATAAA | GAGATGCATGTGCTGTCTCAG |

**Table S7.** List of primers used for constructing dual fluorescent plasmids

| ***Ccl5* promoter-GFP plasmid** | |
| --- | --- |
| Forward primer  (5'-3') | GGGCCAGATATACGCGTTGACTACATTTATCCACTAACGTCATGGTG |
| Reverse primer  (5'-3') | AGCTTAAGTTTAAACGCTAGCAGAGATCTTCATGGTACCCGCG |
| **CMV promoter-mcherry plasmid** | |
| Forward primer  (5'-3') | CTAGCGTTTAAACTTAAGCTTGCCACCATGGTGAGCAAGG |
| Reverse primer  (5'-3') | TGCTGGATATCTGCAGAATTCTTACTTGTACAGCTCGTCCATGCC |
